# A Covalent Calmodulin Inhibitor as a Tool to Study Cellular Mechanisms of K-Ras-Driven Stemness

**DOI:** 10.3389/fcell.2021.665673

**Published:** 2021-07-08

**Authors:** Sunday Okutachi, Ganesh Babu Manoharan, Alexandros Kiriazis, Christina Laurini, Marie Catillon, Frank McCormick, Jari Yli-Kauhaluoma, Daniel Abankwa

**Affiliations:** ^1^Cancer Cell Biology and Drug Discovery Group, Department of Life Sciences and Medicine, University of Luxembourg, Esch-sur-Alzette, Luxembourg; ^2^Drug Research Program, Division of Pharmaceutical Chemistry and Technology, Faculty of Pharmacy, University of Helsinki, Helsinki, Finland; ^3^Helen Diller Family Comprehensive Cancer Center, University of California, San Francisco, San Francisco, CA, United States; ^4^Frederick National Laboratory for Cancer Research, Cancer Research Technology Program, Leidos Biomedical Research, Inc., National Cancer Institute RAS Initiative, Frederick, MD, United States

**Keywords:** K-Ras, calmodulin, covalent inhibitor, cancer stem cell (CSC), BRET

## Abstract

Recently, the highly mutated oncoprotein K-Ras4B (hereafter K-Ras) was shown to drive cancer cell stemness in conjunction with calmodulin (CaM). We previously showed that the covalent CaM inhibitor ophiobolin A (OphA) can potently inhibit K-Ras stemness activity. However, OphA, a fungus-derived natural product, exhibits an unspecific, broad toxicity across all phyla. Here we identified a less toxic, functional analog of OphA that can efficiently inactivate CaM by covalent inhibition. We analyzed a small series of benzazulenones, which bear some structural similarity to OphA and can be synthesized in only six steps. We identified the formyl aminobenzazulenone **1**, here named Calmirasone1, as a novel and potent covalent CaM inhibitor. Calmirasone1 has a 4-fold increased affinity for CaM as compared to OphA and was active against K-Ras in cells within minutes, as compared to hours required by OphA. Calmirasone1 displayed a 2.5–4.5-fold higher selectivity for KRAS over BRAF mutant 3D spheroid growth than OphA, suggesting improved relative on-target activity. Importantly, Calmirasone1 has a 40–260-fold lower unspecific toxic effect on HRAS mutant cells, while it reaches almost 50% of the activity of novel K-RasG12C specific inhibitors in 3D spheroid assays. Our results suggest that Calmirasone1 can serve as a new tool compound to further investigate the cancer cell biology of the K-Ras and CaM associated stemness activities.

## Introduction

Calmodulin (CaM) is a small (17 kDa) dumbbell-shaped signaling adapter, with hundreds of protein interactions and widespread functions in cellular signaling (Tidow and Nissen, 2013). Its two N- and C-terminal lobes each contain two EF-hands that can coordinate altogether four Ca^2+^ ions. Ca^2+^/CaM classically recognizes with high, nanomolar affinity approximately 20-residue long peptides with bulky hydrophobic and basic residues that become encased in the hydrophobic pocket formed by the two lobes. This leads to a significant conformational change of CaM with loss of the central helical structure (Tidow and Nissen, 2013). Non-canonical CaM binders typically possess a polybasic N- or C-terminus with a single lipid modification, which can bind to either or both of the hydrophobic pockets on the N- and C-lobes (Grant et al., 2020b).

CaM has been pursued as a cancer drug target in the 1980s due to its significant role in activating CDKs in the cell cycle (Hait and Lazo, 1986). CaM levels increase during the cell cycle, peaking at G2/M, with a drop-off thereafter (Berchtold and Villalobo, 2014). In addition, CaM seems to be indirectly important for the activation of CDKs that are active in G1 (Taules et al., 1998). CaM distribution is furthermore tightly associated with cell division, as it co-distributes with major structures of the mitotic machinery, such as the central spindle, centrosomes, and the cleavage furrow (Li et al., 1999; Yu et al., 2004). In line with this, CaM inhibitors have been demonstrated to block tumor growth, such as, for example, in multiple myeloma cell line xenografts (Yokokura et al., 2014).

Several non-covalent CaM inhibitors have been developed including the frequently used calmidazolium (Sunagawa et al., 1999) and the highly water-soluble and cell-penetrating naphthalenesulfonamides, such as W-7 (Hidaka et al., 1981; Sengupta et al., 2007). However, the latter can also inhibit CaM targets, such as Ca^2+^/CaM-dependent cyclic nucleotide phosphodiesterase at concentrations > 100 μM (Itoh and Hidaka, 1984; Zimmer and Hofmann, 1984).

Ophiobolin A (OphA) is a potent, covalent CaM inhibitor (Leung et al., 1984). It is a naturally occurring fungal 5-8-5 tricyclic sesterterpene metabolite with broad toxicity against plants, microbes, and cancer cells (Au et al., 2000). It forms an irreversible covalent adduct via C5, C21-dicarbonyl functionalities after intermediate Schiff base formation with Lys 75 or Lys 77 and Lys 148 of CaM (Supplementary Scheme 1). Thus, OphA can react with CaM at a 2:1 ratio, similar to covalent phenothiazine derivatives, which also react with the same lysines (Faust et al., 1987). Despite its potency against CaM, OphA appears to have several other targets, such as phosphatidylethanolamine (Chidley et al., 2016). Together with its broad toxicity across most phyla, this suggests a problematic toxicity spectrum of OphA.

We previously identified OphA as a K-Ras4B (hereafter K-Ras) but not an H-Ras selective inhibitor (Najumudeen et al., 2016). OphA disrupts membrane organization of K-Ras in a CaM-dependent manner and blocked the growth of cancer stem cell enriched spheroids derived from breast cancer cell lines. Up to two K-Ras proteins can directly bind to the two lobes of Ca^2+^/CaM (Agamasu et al., 2019; Grant et al., 2020a). Interestingly, K-Ras has a higher affinity to the C-terminal lobe (*K*_D_ = 0.5 μM) than to the N-terminal lobe (*K*_D_ = 4 μM). Complementary to this, the C-terminal lobe of CaM binds Ca^2+^ with higher affinity compared to the N-terminal lobe (Teleman et al., 1986). This affinity constellation may underpin a Ca^2+^-mediated K-Ras release mechanism. Binding of K-Ras is nucleotide-independent but dependent on the farnesylated C-terminus, while also geranylgeranylation mediates binding albeit with an almost 10-fold lower affinity (Wu et al., 2011; Agamasu et al., 2019; Grant et al., 2020a). In addition, basic residues of the hypervariable region of K-Ras may contribute to the interaction; however, interaction with the prenyl moiety provides the core affinity (Jang et al., 2019; Grant et al., 2020a). In contrast to these more recently established binding determinants, a preference of CaM binding to GTP-K-Ras was previously observed (Villalonga et al., 2001; Abraham et al., 2009).

Experimental data show that palmitoylated Ras isoforms do not interact with CaM (Villalonga et al., 2001) probably because the palmitoyl-moiety would hinder binding to CaM sterically. Thus, its client selectivity could resemble that of PDE6D (PDEδ), a trafficking chaperone that is important for K-Ras plasma membrane localization (Chandra et al., 2011). Indeed, evidence suggests that Ca^2+^/CaM can act as a trafficking chaperone for K-Ras (Fivaz and Meyer, 2005; Grant et al., 2020a), which at high concentration could sequester K-Ras from the membrane as it binds with a lower affinity (*K*_D_ = 4 μM) to nanodiscs than to Ca^2+^/CaM (Gillette et al., 2015). Given that Ca^2+^/CaM has a different K-Ras affinity, release mechanism, cellular distribution, and probably client spectrum than PDE6D, it can be expected that these proteins have overlapping, yet non-redundant chaperone functions. The interaction of CaM with K-Ras is inhibited by the phosphorylation of Ser181 in the C-terminus of K-Ras, while vice versa CaM binding prevents phosphorylation (Alvarez-Moya et al., 2010). Intriguingly, the phosphomimetic S181D has a reduced stemness potential (Wang et al., 2015b). Consistently, the atypical PKC agonist prostratin reduced the growth in several murine tumor models, including pancreatic cancer cell line derived xenografts (Wang et al., 2015b).

Thus, a novel rationale for the development of CaM inhibitors has emerged, which is tied to the K-Ras-dependent induction of cancer cell stemness. While this K-Ras and cancer stemness association may rekindle CaM inhibitor drug development, further dissection of the molecular mechanism is hampered by the fact that three transcribed copies of CaM genes exist (*CALM1-3*) in the human genome (Toutenhoofd and Strehler, 2000). CaM cell biology is therefore difficult to dissect genetically.

Here we describe the identification of the formyl aminobenzazulenone **1**, later named Calmirasone1, as a novel, covalent CaM inhibitor. The compound is synthetically readily accessible in a six-step synthesis from commercially available guaiazulene. Its higher CaM affinity, fast K-Ras directed cellular activity, and > 40-fold reduced unspecific cell toxicity as compared to OphA allow the utilization of Calmirasone1 in acute cell biological experiments.

## Results

### Phenotypic Assessment of Amino Benzazulenones vs. Ophiobolin A

OphA is a potent CaM inhibitor that covalently inactivates its target. We previously showed that it selectively inhibits the functional membrane organization of oncogenic K-Ras. This enabled the inhibition of cancer stem cell features by an as yet not fully defined cellular mechanism (Najumudeen et al., 2016). However, the broad toxicity of OphA limits its application (Chidley et al., 2016).

**SCHEME 1:**
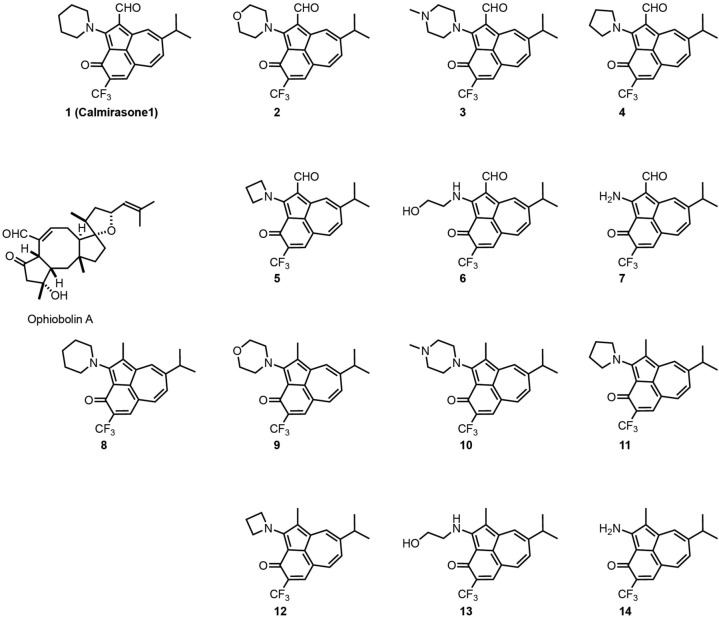
Structures of OphA and the synthetic formyl aminobenzazulenones **(1–7)** and matching aminobenzazulenones **(8–14)**.

In order to identify a less toxic functional analog of OphA for application in cell biological studies, we chose the azulene-derived aromatic benzazulen-3-one scaffold, which is distantly related to the non-aromatic 5-8-5 tricyclic ring framework of OphA. This choice was based only on the chemical similarity, and no additional compound-design or -screening efforts were made. We prepared two series of synthetically easily accessible compounds, formylated and matching non-formylated aminobenzazulenones, containing two or one electrophilic functionality for covalent binding (Scheme 1). The *ortho*-quinone methide electrophile is part of the ring structure and was shown to react readily with primary amines in a nucleophilic aromatic substitution reaction (Kiriazis et al., 2017; Supplementary Data 1); however, other nucleophiles could also react with it. In addition, formyl aminobenzazulenones can undergo a typical Schiff base reaction with amines via their C1-formyl, similar to OphA.

Given that toxicity was the major obstacle to overcome, we first characterized the effects of the compounds in phenotypic assays. Clonogenic growth of breast cancer cell derived 3D tumor spheroids under low adherent conditions is a well-established assay for cancer stem cell properties (Dontu et al., 2003). We were interested in compounds with high K-Ras selectivity in 3D spheroid assays, but low general toxicity in 2D proliferation assays. Consistent with their Ras mutation status, MDA-MB-231 (K-RasG13D) and Hs 578T (H-RasG12D) spheroids were selectively sensitive to KRAS and HRAS knockdown, respectively (Supplementary Figures 1A–D), as shown previously (Siddiqui et al., 2020).

**FIGURE 1:**
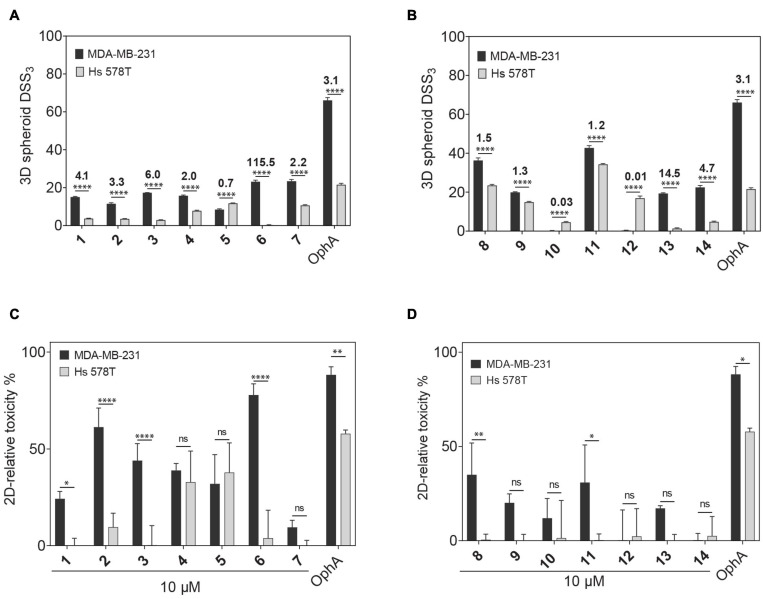
Phenotypic assessment of anticlonogenic and cytotoxic activities of compounds. **(A,B)** A higher DSS_3_ reflects a more potent effect of formyl aminobenzazulenones **(A)** and aminobenzazulenones **(B)** tested at a concentration range of 0.6–40 μM on KRAS-mutant MDA-MB-231 and HRAS-mutant Hs 578T 3D spheroid formation in low attachment condition without serum. Data represent mean values ± SD, *n* ≥ 3. Numbers above the bars indicate the KRAS/HRAS mutant cell line DSS_3_ ratios. **(C,D)** The relative toxicity of formyl aminobenzazulenones **(C)** and aminobenzazulenones **(D)** was assessed in the CellTox Green assay. Cells were grown as 2D adherent monolayers overnight and then treated for 72 h with 1 μM OphA or 10 μM of the indicated benzazulenones. Data represent mean values ± SD, *n* ≥ 2. The statistical significance levels are annotated as *p < 0.05; **p < 0.01; ****p < 0.0001, or ns, not significant.

Compounds showed varying potencies in 3D spheroids with IC_50_ values between 12 and > 40 μM in MDA-MB-231, and 5.2 and > 40 μM in Hs 578T, as compared to 0.3 and 1.8 μM, respectively, for OphA (Table 1 and Supplementary Figures 1E–H). In order to have a more robust descriptor of the compound effect on the clonogenic sphere forming ability of these cells, we used the drug sensitivity score, DSS_3_, which is a normalized area under the dose-response curve value with superior accuracy over IC_50_ determination (Figures 1A,B; Yadav et al., 2014). Thus, it became clear that some compounds had a selectivity for the KRAS-dependent MDA-MB-231 spheroids that was similar to or better than that of OphA.

**TABLE 1:** IC_50_ values of benzazulenones tested on 3D tumorosphere assay.

	MDA-MB-231		Hs 578T	
Compound	IC_50_/μM	logIC_50_ ± SD	IC_50_/μM	logIC_50_ ± SD
1	12	−4.92 ± 0.03	22.5	−4.65 ± 0.04
2	22.8	−4.64 ± 0.06	24.9	−4.61 ± 0.05
3	35	−4.46 ± 0.05	25.8	−4.6 ± 0.1
4	>40	Inconclusive	>40	Inconclusive
5	34.5	−4.46 ± 0.05	13.2	−4.88 ± 0.04
6	>40	Inconclusive	>40	Inconclusive
7	>40	Inconclusive	>40	Inconclusive
8	32.4	−4.5 ± 0.5	10.6	−4.98 ± 0.03
9	19.6	−4.71 ± 0.03	17.4	−4.76 ± 0.01
10	>40	Inconclusive	23.1	−4.64 ± 0.04
11	15.4	−4.81 ± 0.05	5.2	−5.23 ± 0.04
12	>40	Inconclusive	8.5	−5.1 ± 0.1
13	>40	Inconclusive	>40	Inconclusive
14	>40	Inconclusive	>40	Inconclusive
OphA	0.3	−6.54 ± 0.02	1.8	−5.75 ± 0.02

*Data represent mean of three biological repeats (Supplementary Figures 1E–H).*

Next we compared the general cytotoxicity (Figures 1C,D) and antiproliferative activity in cells grown in 2D at 10 μM compound concentration (Supplementary Figures 1I,J). Higher toxicities and antiproliferative effects with selectivity for MDA-MB-231 were generally observed for the formyl aminobenzazulenones. However, none of the compounds tested at 10 μM was as non-specifically toxic as OphA at only 1 μM against HRAS-dependent Hs 578T cells.

### Several Benzazulenones Have a Higher Affinity to CaM Than OphA

High affinity to the target typically reduces off-target toxicities (Bedard et al., 2020). We therefore next determined the *in vitro* affinity of the 14 compounds to the intended target CaM using a fluorescence polarization assay previously developed by us (Manoharan et al., 2019). This assay measures the displacement of a fluorescein-labeled CaM-binding peptide, here derived from plasma membrane calcium-ATPase (PMCA), from purchased CaM by the inhibitors (Table 2 and Supplementary Figures 2A,B).

**TABLE 2:** CaM-binding affinity of compounds after 24-h incubation.

Formyl aminobenzazulenones		Aminobenzazulenones	
Compound	K_d_ ± SD/μM	Compound	K_d_ ± SD/μM
1	0.870.02	8	3.10.3
2	0.230.01	9	1.440.03
3	0.250.02	10	Inconclusive
4	3912	11	0.810.03
5	297	12	6.10.3
6	3110	13	6226
7	454	14	21.40.6

*A fluorescence polarization assay with the fluorescently labeled PMCA peptide as probe was performed. For comparison K_d_(OphA) = 3.5 ± 0.2 μM. While some compounds showed faint autofluorescence under the polarization assay conditions, their emission was too weak as compared to that of fluorescein to interfere with the measurements (Supplementary Figures 2C,D).*

Compounds **2** and **3** showed the highest affinity (15-fold higher affinity than OphA) and **1** being third best (fourfold higher affinity) after 24-h incubation. The fact that OphA had a significantly higher cytotoxic and antiproliferative activity (Figures 1C,D and Supplementary Figures 1I,J), despite lower affinity than six of the compounds, confirms its problematic off-target toxicity (Chidley et al., 2016).

Based on these *in vitro* and the phenotypic data, we calculated a customized *composite drug activity score* to select compounds with most favorable properties in each series, i.e., high overall activity in the 3D spheroid assay, high MDA-MB-231 KRAS-mutant cell line selectivity in 3D spheroid assays, low relative 2D growth toxicity against Hs 578T cells relatively to MDA-MB-231, and high affinity (Supplementary Figures 2E,F). Thus, we selected **1, 2, 3, 8, 9**, and **11** for further analysis.

**FIGURE 2:**
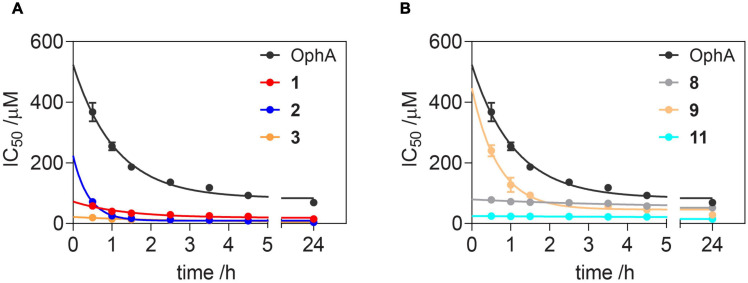
Benzazulenones have higher IC_50_ with less change over time as compared to OphA. Change of effective CaM-binding affinity over time of OphA and formyl aminobenzazulenones **(A)** and aminobenzazulenones **(B)** as measured in the fluorescence polarization assay using F-PMCA peptide as the fluorescent probe. Data represent mean values ± SD, *n* = 2. Binding curves are plotted in Supplementary Figures 2A,B. Derived rate analysis plots are in Supplementary Figures 2G,H.

Of note, the binding affinity of OphA increased over several hours, consistent with the slow covalent Schiff base bond formation and the additional pyrrole adduct formation (Figures 2A,B and Supplementary Scheme 1). By contrast, most benzazulenones immediately showed high IC_50_ ranging from submicromolar to tens of micromolar.

The potency and selectivity of covalent inhibitors are governed by two parameters, namely *K*_i_, the dissociation constant of the initial non-covalent complex, and *k*_2_, the rate of subsequent covalent bond formation (Singh et al., 2011). The latter cannot be too high to avoid non-specific reactivity. Analysis of the reactivity of the top six compounds revealed that formyl aminobenzazulenonens had lower *K*_i_ as compared to non-formylated compounds, suggesting that the formyl moiety increases the non-covalent affinity component (Table 3 and Supplementary Figures 2G,H). This is inconsistent with the hydrophobic binding sites on CaM. However, *k*_2_ increased for **1** and **2**, as well as **8** and **9**, with both **2** and **9** having a covalent bond rate constant as high as that of OphA, which also showed an intermediate *K*_i_ value.

**TABLE 3:** Analysis of *K*_i_ and *k*_2_ and the second-order rate constant *k*_2_*/K*_i_ from data plotted in Figure 2 and processed as described.

Compound	k_2_ ± SD/h^–1^	K_i_ ± SD/μM	k_2_/K_i_/M^–1^ h^–1^
OphA	1.090.04	798	14 × 10^3^
1	0.510.09	5229	10 × 10^3^
2	1.180.09	134	93 × 10^3^
3	0.450.07	116	42 × 10^3^
8	0.350.03	3910	9 × 10^3^
9	1.30.2	22967	6 × 10^3^
11	0.290.05	7834	4 × 10^3^

### Cellular BRET Experiments Confirm K-Ras Selectivity of Top Compound 1

Ras proteins are tightly packed into proteo-lipid membrane signaling complexes called nanoclusters (Abankwa et al., 2007). Fluorescent tagging of Ras proteins with a pair of FRET-enabling fluorophores thus leads to the emergence of nanoclustering-dependent FRET. Loss of this FRET signal reports, however, not only on the loss of nanoclustering but also on any upstream processes, i.e., proper Ras plasma membrane trafficking or lipid modifications (Kohnke et al., 2012).

**FIGURE 3:**
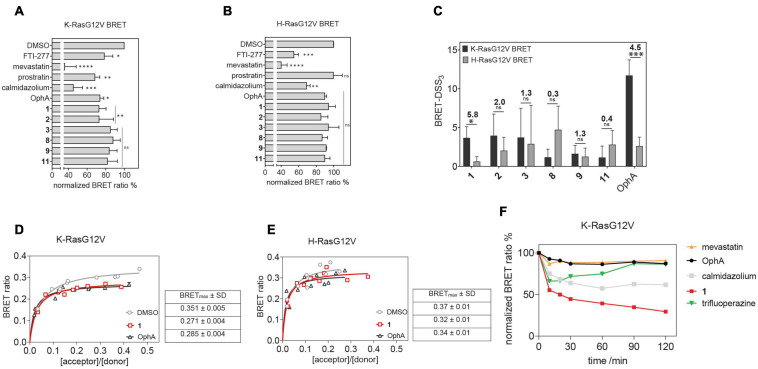
Nanoclustering-BRET assays confirm K-Ras selectivity and fast intracellular activity of compound 1 in cells. **(A,B)** Testing of top six benzazulenones at 20 μM and 24-h exposure in K-RasG12V **(A)** and H-RasG12V **(B)** nanoclustering-BRET assays. Controls are FTI-277 (1 μM), OphA (2.5 μM), mevastatin (10 μM), calmidazolium (20 μM), and prostratin (10 μM). The acceptor/donor (A/D) plasmid ratio of GFP2- and Rluc8-tagged RasG12V was 4/1. Data represent mean values ± SD, *n* = 3. **(C)** BRET-DSS_3_ values for selected six benzazulenones and OphA, derived from dose response analysis of benzazulenones (0.1–80 μM) and OphA (0.3–20 μM) on K-RasG12V and H-RasG12V nanoclustering-BRET data (Supplementary Figures 3A,B). Numbers above the bars indicate the K-RasG12V/H-RasG12V BRET-DSS_3_ ratios. The A/D plasmid ratio was 4/1. Data represent mean values ± SD, *n* ≥ 3. **(D)** K-RasG12V and **(E)** H-RasG12V nanoclustering-BRET donor saturation titration curves showing the effect of OphA (2.5 μM), **1** (20 μM), and vehicle control. Data represent mean values ± SD, *n* = 2. Note that error bars are very small and may not be recognizable. BRET_max_ data represent mean values ± SD, *n* = 2. **(F)** Time-dependent change of K-RasG12V nanoclustering-BRET signal after treatment with **1** (50 μM), OphA (10 μM), mevastatin (10 μM), trifluoperazine (20 μM), and calmidazolium (20 μM). The A/D plasmid ratio was 4/1. Data represent mean values ± SD, *n* ≥ 2. The statistical significance levels are annotated as *p < 0.05; **p < 0.01; ***p < 0.001; ****p < 0.0001, or ns, not significant.

Here we established an analogous nanoclustering-BRET assay by tagging RasG12V proteins with Rluc8, enabling donor emission, and GFP2 as an acceptor. As expected, treatment with mevastatin, which blocks prenyl synthesis, reduced nanoclustering-BRET of both Ras isoforms fairly indiscriminately, while treatment with a farnesyl transferase inhibitor (FTI-277) selectively (1.4-fold) decreased H-Ras nanoclustering-BRET (Figures 3A,B), due to the alternative prenylation of K-Ras, as described before (Kohnke et al., 2012).

The inhibition of the trafficking chaperone PDE6D, which facilitates plasma membrane trafficking, in particular of K-Ras, decreases selectively K-Ras nanoclustering-FRET (Siddiqui et al., 2020). In agreement with CaM acting as a trafficking chaperone that can likewise promote forward trafficking to the plasma membrane, we observed a K-Ras selective reduction of nanoclustering-BRET after CaM inhibition with calmidazolium (1.5-fold) and OphA (1.2-fold). The atypical PKC agonist prostratin, which would stimulate K-Ras-Ser181 phosphorylation and thus block CaM binding, had a similar selectivity (1.5-fold) as the CaM inhibitors.

We then tested the top six compounds in this assay in order to directly assess their in cellulo K-Ras selectivity. While most compounds appeared to show some level of K-Ras selectivity (all < 1.3-fold) when compared at 20 μM and 24-h exposure (Figures 3A,B), testing over a wider concentration range revealed distinct potencies and selectivities (Supplementary Figures 3A,B). We employed the DSS analysis adapted to BRET-data (BRET-DSS_3_) to quantify these activities (Figure 3C). While, again, overall BRET-activity was highest for OphA, K-Ras selectivity was highest for **1**. All other compounds had lower and non-significant selectivities. By doing a BRET donor saturation titration analysis, we further confirmed that **1** has a similar K-Ras vs. H-Ras selectivity as OphA (Figures 3D,E and Supplementary Figures 3C–F).

Compound **1** affinity to CaM changes less over time than that of OphA, suggesting that it assumes its full activity faster (Figure 2), which could be advantageous if true also in cellular applications. We therefore tested this property in cells using the K-Ras BRET biosensor. In order to see clear effects at short exposure times, all compound concentrations were increased. OphA showed no significant BRET change during the 2-h treatment timeframe, consistent with the significant time it requires for high affinity binding (Figure 2). Likewise, mevastatin did not cause any reduction in the BRET signal, as it has to block metabolic pathways for farnesyl- and geranylgeranyl-pyrophosphate synthesis and therefore acts slowly after protein turnover. In agreement with the *in vitro* data, **1** showed a 38% reduction in the BRET signal within 10 min of treatment (Figure 3F). It was therefore even more active acutely in cells than the non-covalent CaM inhibitor trifluoperazine (*K*_d_ = 1.35 μM) or calmidazolium (*K*_d_ = 13.5 nM) (Manoharan et al., 2019).

### BRET Experiments Confirm K-Ras/CaM Disrupting on-Target Activity in Cells

**FIGURE 4:**
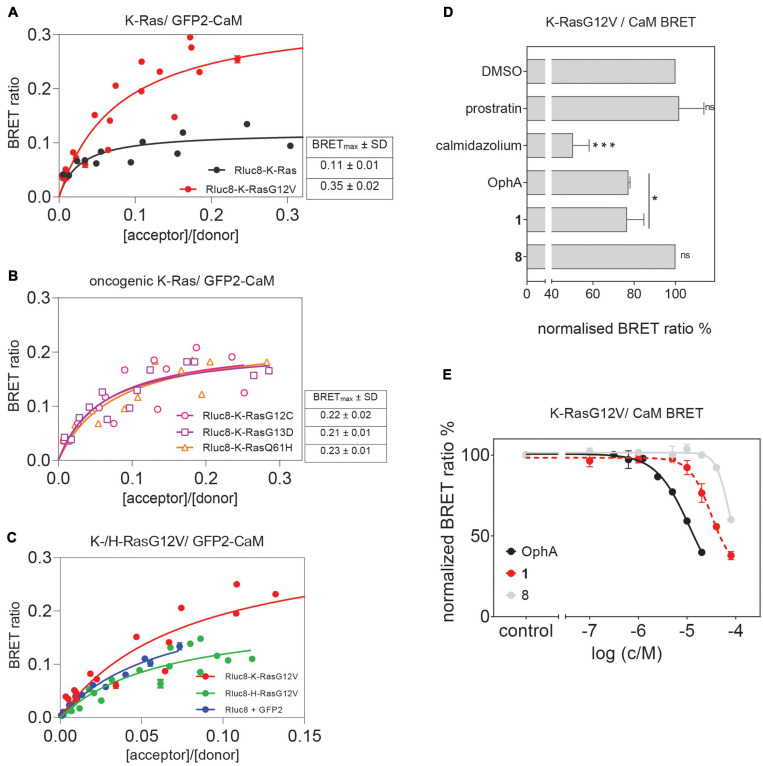
Cellular K-RasG12V/CaM interaction BRET confirms on-target activity of compound 1 in cells. **(A)** BRET donor saturation titration curves between Rluc8-K-Ras or Rluc8-K-RasG12V and N-terminally GFP2-tagged CaM. **(B)** BRET donor saturation titration curves between the Rluc8-tagged K-Ras oncogenic mutants (K-RasG12C, K-RasG13D, and K-RasQ61H) with GFP2-CaM. The BRET_max_ data represent mean values ± SD, *n* ≥ 2. **(C)** BRET donor saturation titration curves between Rluc8-K-RasG12V or Rluc8-H-RasG12V and GFP2-CaM. Plasmids expressing Rluc8 and GFP2 proteins alone were used as controls for non-specific interaction. **(D)** Compounds calmidazolium (20 μM), prostratin (20 μM), or OphA (5 μM), as well as formyl aminobenzazulenone **1** (20 μM) or non-formylated counterpart aminobenzazulenone **8** (20 μM) were tested using the Rluc8-K-RasG12V/GFP2-CaM BRET reporter. The A/D plasmid ratio was 9/1. Data represent mean values ± SD, *n* ≥ 2. **(E)** Dose-response analysis of compound **1** and its non-formylated derivative **8** as compared to OphA using Rluc8-K-RasG12V/GFP2-CaM BRET signal. The A/D plasmid ratio was 9/1. Data represent mean values ± SD, *n* ≥ 2. The statistical significance levels are annotated as *p < 0.05; ***p < 0.001, or ns, not significant.

Previously, a preference of CaM binding to active GTP-K-Ras was observed (Villalonga et al., 2001; Abraham et al., 2009). In agreement with these data, we observed in cells a higher BRET of N-terminally Rluc8-tagged K-RasG12V with GFP2-CaM than that of non-oncogenic K-Ras (Figure 4A and Supplementary Figure 4). Likewise, higher BRET levels were confirmed with three additional oncogenic mutants of K-Ras (Figure 4B and Supplementary Figures 4A,B). Furthermore, in line with previous reports (Villalonga et al., 2001), K-RasG12V (BRET_max_ = 0.35 ± 0.02) displayed a significantly (*p* = 0.001, unpaired *t*-test) higher cellular BRET ratio with GFP2-CaM than H-RasG12V did (BRET_max_ = 0.20 ± 0.02), which remained at or below control levels (Figure 4C and Supplementary Figure 4). This could explain the preferential effect on K-Ras nanoclustering-BRET by CaM inhibitors (Figure 3).

In order to have a high dynamic range of the BRET signal, we used the Rluc8-K-RasG12V/GFP2-CaM BRET pair to directly assess the effect of modulators of the K-Ras/CaM interaction. Both CaM inhibitor calmidazolium and OphA significantly reduced the BRET signal. Surprisingly, prostratin did not have an effect at the tested concentration (Figure 4D).

To further delineate the structural requirements for the on-target, K-RasG12V/CaM disrupting activity, we tested formyl aminobenzazulenone **1** in comparison to the closely related, but less active aminobenzazulenone derivative **8**, which lacks the C1-formyl group. Compound **1** (IC_50_ = 31 ± 2 μM) was significantly more active than **8** (IC_50_ = 70 ± 11 μM; p = 0.03), also when tested over a wider concentration range (Figure 4E). Yet, OphA remained the most effective compound in this cellular assay after a 24-h-long exposure (IC_50_ = 12 ± 2 μM).

### Dependence of the Activity of top Compound 1 on Lysines 75, 77, and 148 of CaM

**FIGURE 5:**
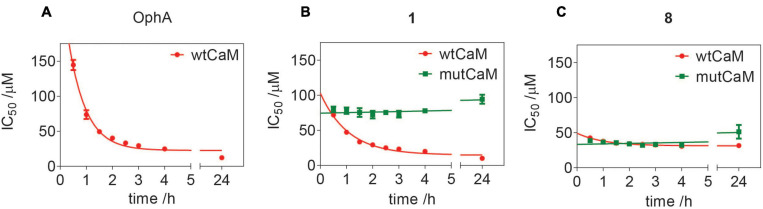
Assessment of lysine-dependent CaM-binding activity of OphA, formyl aminobenzazulenone **1**, and aminobenzazulenone **8**. Time course of lysine-dependent CaM-binding activity of OphA **(A)**, compound **1 (B)**, and compound **8 (C)** as measured in the fluorescence polarization assay using F-CaMKII peptide as the fluorescent probe. OphA displayed negligible binding with mutCaM compared to wtCaM; hence, no IC_50_ values could be derived (Supplementary Figures 5A,B).

We previously showed that the K-Ras directed effect of OphA is abolished if a lysine mutant of CaM is expressed to rescue the knockdown of endogenous CaM (Najumudeen et al., 2016). In this mutant CaM (mutCaM), lysines 75, 77, and 148 were replaced by glutamine, i.e., those residues that were reported to be modified by OphA (Kong Au and Chow Leung, 1998). To assess the dependence of compound **1** binding to CaM on these lysine residues, we again employed a fluorescence polarization assay using in-house purified, His-tagged CaM or mutCaM. Both variants bound to the fluorescein-labeled peptide of Ca^2+^/calmodulin-dependent kinase II (CaMKII) (Manoharan et al., 2019). As observed before (Figure 2), the affinity of OphA to wild-type (wt) CaM increased over several hours, while no binding was observed to mutCaM (Figure 5A and Supplementary Figures 5A,B), as reported previously (Najumudeen et al., 2016; Manoharan et al., 2019). By contrast, compound **1** also displayed binding to mutCaM; however, as compared to wt CaM, the affinity did not increase over time (Figure 5B and Supplementary Figures 5E,F). This was different for the non-formylated counterpart **8**, which showed the same binding affinity for wt CaM and mutCaM over time (Figure 5C and Supplementary Figures 5E,F). The comparison of the activities of all three compounds suggests that the K75Q, K77Q, and K148Q mutations in the mutCaM have rendered CaM partially insensitive to **1** and **8** binding. It furthermore shows that the lysine-dependent increase in affinity over time of compound **1** depends on the C1-formyl, which could form a Schiff base bond in a slow reaction.

### Activity in Cell Proliferation Assays Correlates With the K-Ras Dependence of Cancer Cell Lines

**FIGURE 6:**
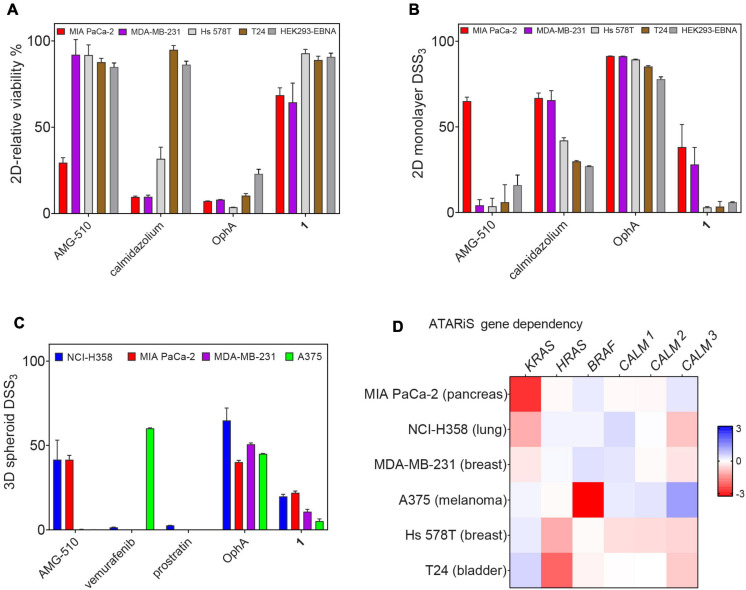
Benchmarking of top compound 1 in several cancer cell lines. **(A)** The relative 2D viability of various cell lines following single dose treatment with AMG-510 (1 μM), calmidazolium (2.5 μM), OphA (1 μM), and **1** (10 μM) was assessed using the alamarBlue assay. Cells were grown as 2D adherent monolayers overnight and then treated for 72 h with indicated compounds. Data represent mean values ± SD, *n* ≥ 3. **(B)** DSS_3_ measuring the effects of AMG-510 (0.003–40 μM), calmidazolium (0.3–40 μM), OphA (0.3–40 μM), and **1** (0.6–80 μM). Cells were grown as 2D adherent monolayers overnight and then treated for 72 h. Results represent mean values ± SD, *n* = 3. **(C)** DSS_3_ measuring the effects of AMG-510 (0.6–40 μM), vemurafenib (0.3–20 μM), prostratin (0.6–80 μM), OphA (0.3–20 μM), and **1** (1.3–80 μM). Cells were grown as 3D spheroids for 72 h then treated with compounds for another 72 h before alamarBlue viability measurements. Data represent mean values ± SD, *n* ≥ 2. **(D)** Heatmap of ATARiS gene sensitivity scores obtained from the project DRIVE database for KRAS-dependent cell lines (MIA PaCa-2, NCI H358, and MDA-MB-231) and HRAS-dependent cell lines (Hs 578T and T24). Negative values (red) indicate sensitivity of the cell line proliferation to the knockdown of shown genes, while positive (blue) indicates the opposite.

Unspecific, broad toxicity against KRAS (MDA-MB-231, MIA PaCa-2) and HRAS mutant (Hs 578T, T24) cancer cell lines, as well as HEK293-EBNA cells, is a major issue of OphA (Figure 6A). This broad toxicity appears to greatly contribute to the high “anti-cancer cell activity” that is observed with this compound and clearly contrasts to the KRAS mutant cancer cell line selectivity seen with calmidazolium and **1** (Figure 6B and Supplementary Figures 6A–E). Of note, the latter has a background activity against HRAS mutant cancer cells that was as low as that of the covalent K-RasG12C inhibitor AMG-510.

When compounds were compared in 3D spheroid growth assays, the significant potency difference between clinical compounds and **1** became, however, more obvious than in 2D assays. Both AMG-510 and vemurafenib selectively and potently abolished the growth of the K-RasG12C- and BRAF-V600E-mutant cancer cell 3D spheroids, respectively, with basically no activity against other cancer cell spheroids (Figure 6C and Supplementary Figures 6F–I). Compound **1** had a visibly lower activity, yet the activity profile seemed to correlate with the KRAS dependence of the cancer cell lines (Figures 6C,D). Again, OphA appeared highly potent, yet clearly at the cost of its broad toxicity (Figures 6A,C). These data are in line with a much improved on-target activity of **1** as compared to OphA.

### The Best Tool Compound 1 Can Be Utilized in Cell Biological Experiments

Given the significantly reduced unspecific toxicity of **1** as compared to OphA, we tested its application in cell biological experiments. CaM dynamically localizes to centrosomes, spindle, and other structures during mitosis, and its inhibition is known to affect proper cleavage furrow formation, which can lead to multipolarity (Yu et al., 2004; Wu et al., 2010).

**FIGURE 7:**
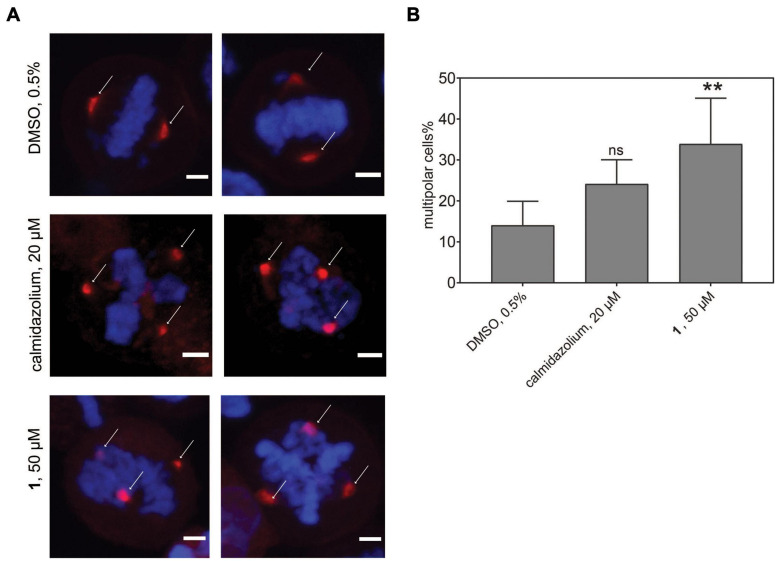
Phenotypic effects of CaM inhibitors on centrosome numbers. **(A)** Representative images for bipolar normal (DMSO 0.5%, top) and multipolar centrosomes in HeLa cells after acute treatment for 2 h with calmidazolium (20 μM, middle) **1** (50 μM, bottom). Hela cells expressing mCherry-wtCaM (red) cells were synchronized with nocodazole to the G2/M phase for 16 h. Then cells were treated with compounds and simultaneously with the protease inhibitor MG132 (10 μM). Arrows indicate predominant localization of mCherry-wtCaM on the centrosomes during mitosis. DNA was stained with DAPI (blue). Scale bar, 5 μm. **(B)** The multipolar phenotype was quantified for each treatment from images containing 35 to 70 cells per condition. Data represent mean values ± SD, *n* = 3. Statistical significance was evaluated with Fisher’s exact test. The statistical significance levels are annotated as **p < 0.01, or ns, not significant.

In order to track this phenotype and the CaM distribution, we transfected HeLa cells with a mCherry-CaM construct, which primarily localized to centrosomes in mitotic cells (Figure 7A). When these cells were synchronized and treated with the potent, non-covalent CaM inhibitor calmidazolium, an increased fraction of multipolar cells with multiple mCherry-CaM-positive centrosomes was observed. As expected from the faster in-cell activity observed in BRET experiments (Figure 3F), this phenotype was significantly pronounced with **1** (Figure 7B), confirming its utility in cell biological experiments. Finally, we named compound **1**, the best performing tool compound, **Calmirasone1**.

## Discussion

We have here identified compound **1**, which we named **Calmirasone1,** a synthetically well-accessible, high affinity covalent CaM inhibitor with fast cellular K-Ras selectivity and significantly lower toxicity than the natural product counterpart OphA. While the current potency and properties of **Calmirasone1** do not fit for a compound with future medical applications, our data support its intended utility as a tool compound in cell biological applications to study CaM-dependent cellular processes. Such tool compounds are important also for drug development, as they can foreshadow some on-target issues and reveal crucial mechanistic features of actual drug candidates.

Several of our compounds bound to CaM with submicromolar affinity, with **Calmirasone1** binding four times better than OphA. Comparison of **Calmirasone1** and **8** affinities with purified wt and mutant CaM suggests that the affinity binding component that remained constant over time was independent of the C1-formyl (Figure 5). This immediate high affinity could have been of non-covalent or actually also of covalent nature. Given that a second reactive group with covalent binding potential (*ortho* quinone-methide, *o*-QM) is present in both **Calmirasone1** and **8**, it is possible that this electrophile mediates additional covalent binding to lysine residues other than those three mutated lysines in mutCaM (Supplementary Data 1), or alternatively cysteines. However, nucleophilic cysteines are not present in the studied CaM variants. Based on previous synthetic studies, the *o*-QM reactivity toward nucleophiles (amino or thiol) can be very fast (within minutes) and proceeds via a nucleophilic aromatic substitution (S_N_Ar-type) reaction (Kiriazis et al., 2017). We currently lack evidence whether this second electrophile is also engaged covalently.

Our rate analysis (Table 3) shows that the compound with the highest second order rate constant was **2**, followed by **3**. However, as our cellular BRET-data in Figure 3C indicate, this increased reactivity appears to come at the cost of selectivity. We see a maximal selectivity for K-RasG12V vs. H-RasG12V for **1**, which has intermediate parameters, in agreement with a balance between sufficient affinity and a moderate reactivity. In agreement with the slow Schiff-bond formation, we find rate constants that are several thousand-fold lower than those of Lys-reactive compounds with a vinyl sulfone as warhead, such as the CDK2-inhibitor NU600 (k_2_/K_i_ = 5.0 × 10^3^ M^–1^ s^–1^) (Anscombe et al., 2015). However, the α-hemoglobin targeting compound GBT440 (Voxelotor) with a formyl warhead similar to our compounds has a second-order rate constant comparable to what we found for our benzazulenones (k_2_/K_i_ = 15 × 10^3^ M^–1^ h^–1^) (Center for Drug Evaluation and Research, 2017; Metcalf et al., 2017).

We speculate that the formyl-independent binding component significantly improves the unspecific toxicity of compounds **Calmirasone1** and **8** (Figures 1C,D). However, the major, slower affinity increase stems from the C1-formyl and depends on mutated lysines 75, 77, and 148. This is consistent with the formyl as a hard electrophile reacting with lysine as a hard nucleophile. The typically slow Schiff base formation may, therefore, explain the slow increase in the effective affinity (Figure 5B). The formyl substituent is furthermore beneficial, as it lowers the relatively high clogP, thus potentially increasing water solubility of these not very drug-like molecules. However, drug entry into cells can be an active process that depends on transporters from the solute carrier protein (SLC) family (Girardi et al., 2020). In addition, passive entry is typically characterized by the compound specific partitioning coefficients. Both passive and active entry may explain why we observed distinct time courses for the inhibitors to become active in cells against K-RasG12V membrane anchorage (Figure 3F).

Currently, the structural basis for CaM inhibition by OphA is not known. However, similar to other non-covalent inhibitors, such as trifluoperazine, the conformational dynamics of CaM may change dramatically upon inhibitor binding, collapsing the original dumbbell-shaped molecule into a compact globular structure (Vandonselaar et al., 1994). We speculate that covalent inhibitors such as OphA and the here tested compounds would have a similar effect on the conformation and therefore activity of CaM to bind its canonical and non-canonical clients, such as K-Ras.

The Ras nanoclustering-dependent BRET assay that we used before successfully in the FRET format to assess the Ras selectivity close to the mechanistic target K-Ras (Najumudeen et al., 2016; Posada et al., 2016) is sensitive to the disruption of Ras membrane anchorage and correct plasma membrane trafficking. CaM was recently established as a K-Ras trafficking chaperone, which can essentially act as a solubilizing factor to shield the farnesyl tail from the aqueous environment of the cytoplasm (Grant et al., 2020a). Therefore, the drop in K-Ras nanoclustering-BRET with CaM inhibitors is consistent with CaM being a trafficking chaperone for K-Ras (Grant et al., 2020b).

We have previously demonstrated similar changes in membrane anchorage of K-Ras with the inhibition of PDE6D, another prominent trafficking chaperone of K-Ras (Siddiqui et al., 2020). For PDE6D, clients such as H-Ras that are in addition palmitoylated cannot bind as long as they are palmitoylated (Chandra et al., 2011; Dharmaiah et al., 2016). This establishes an effective K-Ras over H-Ras selectivity for PDE6D inhibition-induced cell growth effects (Siddiqui et al., 2020). Grant et al. (2020a) recently derived singly lipidated polybasic termini of proteins as non-canonical CaM interaction sequences. Consistently, K-Ras but not H-Ras or N-Ras bind to CaM (Villalonga et al., 2001). It can be speculated that any additional palmitoylation would sterically hinder access to CaM, making palmitoylated Ras isoform clients only if they are in their non-palmitoylated state (Agamasu et al., 2019). This would explain why the potent CaM inhibitor calmidazolium decreased the BRET signal of H-Ras, albeit to a lesser extent than that of K-Ras (Figures 3A,B).

The highly potent calmidazolium, as well as the covalent inhibitors OphA and **Calmirasone1**, significantly disrupted K-Ras/CaM-BRET in cells. By contrast, the PKC agonist prostratin had no effect on K-Ras/CaM-BRET, but on K-RasG12V nanoclustering BRET. It may therefore be that prostratin exerts its K-Ras selectivity by a different mechanistic route than the inhibition of K-Ras/CaM interaction. Interestingly, prostratin had almost no effect on cell growth in 3D spheroid assays (Figure 6C).

Clonogenic 3D spheroid growth depends on stemness associated asymmetric and symmetric division processes of cancer cells with stemness traits (Cicalese et al., 2009). Accordingly, **Calmirasone1** demonstrates an efficacy against 3D spheroid growth that correlates with the KRAS dependence of the tested cell lines. In this regard, it is noteworthy that the DSS_3_ potency of **Calmirasone1** reaches already approximately 50% of AMG-510, the K-RasG12C inhibitor that is currently being evaluated in the clinic (Hong et al., 2020). However, a much larger number of cell lines would have to be tested to demonstrate a correlation between compound activity and anticipated K-Ras/CaM targeting mechanism. For instance, both cell lines that were employed here also carry mutations in BRAF (MDA-MB-231) or in TP53 (both MDA-MB-231 and Hs578T). For both B-Raf and p53, connections to CaM signaling have been reported (Ren et al., 2008; Taylor et al., 2020); hence, the cell killing activity may relate to multiple pathways that are affected downstream of CaM.

In addition, we could demonstrate the benefits of using **Calmirasone1** as a tool compound in cell biological experiments, which are not possible with OphA due to its high toxicity. We observed the induction of multipolar cells by CaM inhibitor treatment. Inhibition of CaM affects multiple processes during cell division, notably cleavage furrow formation (Yu et al., 2004). While failure of cytokinesis can lead to chromosomal instability and therefore a hallmark of cancer cells, the exact nature of the multipolar phenotype and additional effects could also play a role in the ultimately cell growth inhibiting effect of CaM inhibition (Wu et al., 2010). Interestingly, a different compound that induces multipolar acentrosomal spindles was found to selectively kill tumor cells (Wang et al., 2015a). In our cell biological experiments, **Calmirasone1** (*K*_d_ = 0.87 ± 0.02 μM) can be considered more effective than non-covalent calmidazolium (*K*_d_ = 13.5 nM) (Figures 3F, 7B). While **Calmirasone1** was used at 2.5-fold higher concentration, the 64-fold affinity difference between these two compounds suggests a > 25-fold higher effectivity of **Calmirasone1**. Therefore, **Calmirasone1** can be used to acutely (within 30–60 min) perform a chemical knockdown of CaM in cells in a more efficient manner than with the most potent non-covalent inhibitor calmidazolium.

Covalent inhibitors have experienced a renaissance in the past few years (Singh et al., 2011). Our novel covalent CaM inhibitor **Calmirasone1** will add to the arsenal of covalent tool compounds to study in particular the cell biology of K-Ras/CaM-driven stemness processes.

## Materials and Methods

### Compound Synthesis

Synthesis of chemical compounds and their analytical information are given in Supplementary Data 1.

### Expression Constructs and siRNA

Most expression constructs described in the study were produced by multisite gateway cloning as described (Wall et al., 2014; Supplementary Table 1). For plasmids used in the BRET assay, three entry clones, with compatible LR recombination sites, encoding the CMV promoter, Rluc8, or GFP2 tag, and the gene of interest were recombined with a destination vector, pDest-305 or pDest-312, using the Gateway LR Clonase II enzyme mix (cat. no. 11791020, Thermo Fisher Scientific). The reaction mix was transformed into the ccdB-sensitive *E. coli* strain DH10B (cat. no. EC0113, Thermo Fisher Scientific), and positive clones were selected using ampicillin. pDest527-His-wtCaM and pDest527-His-mutCaM were produced from the LR reaction between the pDest-527 vector with either entry clone pDONR221-wtCaM or pDONR221-mutCaM. The N-terminally GFP2-tagged CaM plasmid pDest-CMV-GFP2-CaM was cloned at Genecust (France) and amplified in the *E. coli* CopyCutter EPI-400 strain (cat. no. C400CH10, Lucigen) according to the instruction of the manufacturer. All the plasmids were verified by sequencing. Expression and localization of the Ras and CaM fusion proteins were confirmed by confocal microscopy (Supplementary Figure 7). Protein sequences of all expression constructs are given in the Supplementary Material section. pmCherry-wtCaM was previously described (Manoharan et al., 2019).

Knockdown of CALM1 was done using a master mix of multiple siRNA against the CALM1 transcript [QIAGEN Hs_CALM1, siRNAs: SI00092925 (CALM1_4), SI02224215 (CALM1_5), SI02224222 (CALM1_6), and SI03649268 (CALM1_8)]. For the knockdown of specific Ras isoforms, we used KRAS (K-Ras4A + K-Rras4B- L-005069-00) and HRAS (L-004142-00) Dharmacon On-Target plus siRNA SMARTpools. Scrambled siRNA control was from QIAGEN (cat. no. 102276).

### Commercial Chemical Inhibitors

Fluorescein-labeled CaMKII and PMCA peptide were from Pepmic, China, and Genscript, United States, respectively (Manoharan et al., 2019). DMSO was from PanReac-AppliChem (cat. no. A3672, ITW Reagents). Sources of the inhibitors used in the study are listed below.

**Table:** 

Compound	Source	Catalog number
Ophiobolin A	Santa Cruz	sc-202266
Mevastatin	Alfa Aesar	J61357
FTI-277	BioVision	2874
Prostratin	Sigma-Aldrich	P0077
Calmidazolium	Santa Cruz	sc-201494
AMG-510	MedChem Express	HY-114277
Vemurafenib	Selleckchem	S1267
Trifluoperazine	Cayman	15068
Benzethonium chloride	Sigma-Aldrich	53751

### RT-qPCR Analysis of Gene Transcript Knockdown

MDA-MB-231 and Hs 578T cells were seeded in 12-well plates and transfected with indicated amounts of siRNAs. Where required, siRNA was transfected into cells using a Lipofectamine RNAiMAX (cat. no. 13778075, Thermo Fisher Scientific) reagent according to the instruction of the manufacturer. After 24 h of transfection, total RNA was isolated using NucleoZol (cat. no. 7040404, Macherey-Nagel) according to the manufacturer protocol. Reverse transcription was performed with 1 μg of total RNA using SuperScript III Reverse Transcriptase (cat. no. 18080093, Thermo Fisher Scientific). The knockdowns of KRAS, HRAS, and CALM1 gene transcripts were analyzed by real-time qPCR using SsoAdvanced Universal SYBR Green Supermix (cat. no. 1725274, BIO-RAD) on the CFX-connect real-time PCR instrument (BIO-RAD). The transcripts were selectively amplified using specific primers producing amplicons for total KRAS (both KRAS4A and KRAS4B), HRAS, and CALM1. The gene transcript ACTB encoding for β-actin was used as reference. The following primers were used (Tsai et al., 2015): for total KRAS, forward 5′-tacagtgcaatgagggacca-3′, reverse 5′-tcctgagcctgttttgtgtct-3′ (amplicon 206 bp); for HRAS, forward 5′-ctgaccatccagctgatcca-3′, reverse 5′-tggcaaacacacacaggaag-3′ (amplicon 196 bp); for ACTB, forward 5′-ggggtgttgaaggtctcaaa-3′; reverse 5′- ggcatcctcaccctgaagta-3′ (amplicon 203 bp); for CALM1, forward 5′-gctcgcaccatggctgat-3′, reverse 5′- tgttggg ttctgacccagtg-3′ (amplicon 144 bp).

### 3D Spheroid Assays

3D spheroid formation assays were performed in 96-well low-attachment, suspension culture plates (cat. no. 655185, Cellstar, Greiner Bio-One) under serum-free condition. About 1,000 (MDA-MB-231, NCI-H358, and MIA PaCa-2) or 2,500 (Hs 578T) cells per well were seeded in 50 μl of either an RPMI medium (cat. no. 52400-025, Gibco, Thermo Fisher Scientific) (MDA-MB-231, A375, and NCI-H358) or DMEM (cat. no. 41965-039, Gibco, Thermo Fisher Scientific) (Hs 578T and MIA PaCa-2), containing 0.5% MethoCult (cat. no. SF H4636, Stemcell technologies), 1x B27 (cat. no. 17504044, Gibco, Thermo Fisher Scientific), 25 ng/ml EGF (cat. no. E9644, Sigma-Aldrich), and 25 ng/ml FGF (cat. no. RP-8628, Thermo Fisher Scientific). Cells were cultured for 3 days and then treated with compounds or vehicle control (DMSO 0.1% v/v in growth medium) for another 3 days. The cells were supplemented with a fresh growth medium on the third day together with the drug treatment. For knockdown experiments, cells were seeded in 12-well plates and treated with either 50 nM scrambled siRNA (cat. no. 1022076, QIAGEN) or indicated concentrations of siRNAs. Next day, cells were collected by trypsinization and re-plated into 96-well plates for 3D spheroid suspension culture.

Spheroid formation efficiency was analyzed by an alamarBlue assay reagent (cat. no. DAL1100, Thermo Fisher Scientific). A 10% final volume of the alamarBlue reagent was added to each well of the plate and incubated for 4 h at 37°C. Then the fluorescence intensity was measured using the FLUOstar OPTIMA plate reader (BMG Labtech, Germany) with an excitation wavelength of 560 ± 5 nm and an emission wavelength of 590 ± 5 nm. The obtained fluorescence intensity data were normalized to vehicle control corresponding to 100% sphere formation and the signal after 100 μM benzethonium chloride treatment, which killed all cells (i.e., maximum inhibition of sphere formation).

### Drug Sensitivity Score (DSS) Analysis

To quantitatively profile the drug sensitivity with a more robust parameter than the IC_50_ or EC_50_ values, the drug sensitivity score (DSS) analysis was employed. DSS values are essentially normalized area under the curve (AUC) measures of dose-response inhibition data (Yadav et al., 2014). Drug response data files (in Excel) ready for online analysis were prepared according to the example file obtained from the DSS pipeline website, called Breeze^1^ (Potdar et al., 2020). Either raw fluorescence intensity measurements or normalized % inhibition data (for BRET assay analysis) were uploaded.

The output file provides several drug sensitivity measures including EC_50_ and AUC. We plotted the DSS_3_ value (Yadav et al., 2014), which was calculated as

$$\begin{array}{c} DSS_{3}=DSS_{2}\frac{x_{2}-x_{1}}{C_{max}-C_{min}} \end{array}$$

where DSS_2_ is given by the equation $DSS_{2}=\frac{DSS_{1}}{\log a}$

and DSS_1_ is given by the equation $DSS_{1}=\frac{AUC-t\left(x_{2}-x_{1}\right)}{\left(100-t\right)\left(C_{max}-C_{min}\right)}$

DSS_3_ was employed to emphasize drugs that obtain their response area over a relatively wide dose window, as compared to drugs that show increased response only at the higher end of the concentration range. After logistic fitting of the dose-response inhibition data, the area under the curve (AUC) was determined as the exact solution. A 10% minimal activity threshold (t) was set. The maximum (C_max_) and minimum (C_min_) concentrations were used for screening of the inhibitors, with C_max_ = x_2_ and x_1_ concentration with minimal activity t. The parameter a is the value of the top asymptote, which can be different from 100% inhibition as obtained from 100 μM benzethonium chloride treatment.

### 2D Cell Toxicity and Viability Assays

Hs 578T and MDA-MB-23 cells cultured in complete DMEM and RPMI medium [i.e., supplemented with 10% FBS (cat. no. 10270–098, Gibco, Thermo Fisher Scientific), 2 mM L-glutamine (cat. no. 25030-024, Thermo Fisher Scientific)], respectively, were plated onto 96-well F-bottom cell culture plates (cat. no. 655180, Cellstar, Greiner Bio-One) at a density of 1,000 cells (MDA-MB-231, MIA-PaCa-2, T24, and HEK293-EBNA) and 2,500 cells (Hs 578T) per well grown for 24 h. Freshly thawed aliquots of test compounds were then added at indicated concentrations. DMSO 0.2% v/v in a growth medium was used as the vehicle control. Plates were further incubated for 72 h. The cell viability and cell toxicity effects were analyzed by alamarBlue and CellTox Green (cat. no. G8743, Promega) assays, respectively. A 10% final volume of the alamarBlue reagent was added to each well of the plate and incubated for 4 h at 37°C. Then the fluorescence intensity was measured using the FLUOstar OPTIMA plate reader (BMG Labtech) with an excitation wavelength of 560 ± 5 nm and an emission wavelength of 590 ± 5 nm. The obtained fluorescence intensity data were normalized to vehicle control (100% viability).

For the CellTox Green assay, 100 μl of the 2× CellTox Green reagent was added to each well of a 96-well plate containing 100 μl of the medium. The plate was protected from light and incubated for 15 min at 37°C, then orbitally shaken for 1 min at 700–900 rpm. The fluorescence intensity was measured using the Clariostar plate reader (BMG Labtech) with an excitation wavelength of 485 ± 4 nm and an emission wavelength of 530 ± 4 nm. The obtained fluorescence intensity data were normalized to vehicle control (0% toxicity).

### Protein Purification

Our numbering of CaM follows (Kong Au and Chow Leung, 1998) with Ala being the first amino acid in human CaM, as the N-terminal methionine of CaM is removed in most organisms (Halling et al., 2016). His-wtCaM and His-mutCaM were expressed in *E. coli* BL21 Star (DE3)pLysS (cat. no. C602003, Thermo Fisher Scientific). pDest527-His-wtCaM and pDest527-His-mutCaM plasmids encoding wild-type human CaM and CaM with K75Q, K77Q, and K148Q mutations, respectively, were transformed into *E. coli* BL21 Star (DE3)pLysS and grown in a Luria Broth medium supplemented with ampicillin (100 μg/ml). At A_600_ of 0.6–0.8, the culture was induced with 0.5 mM of isopropyl β-D-thiogalactopyranoside and expressed for 16 h at 25°C. Cells were collected by centrifugation and incubated on ice for 30 min. The cell suspension was sonicated in a lysis buffer (20 mM HEPES, pH 7.6, 150 mM NaCl, 5 mM MgCl_2_, 0.5 mg/ml lysozyme, and DNase I). The lysates were clarified by centrifugation at 18,000 *g* for 30 min at 4°C. The soluble fractions were subjected to protein purification.

The His-tagged proteins were purified on HisTrap^TM^ HP Prepacked Columns (GE Healthcare) using the chromatography system ÄKTAprime plus (GE Healthcare). The columns were equilibrated in a buffer composed of 50 mM Tris HCl, pH 7.5, 150 mM NaCl, and 35 mM imidazole, and the His-tagged proteins were eluted in an elution buffer containing 250 mM of imidazole. The eluted fractions were dialyzed for 16 h at 4 °C in a buffer composed of 50 mM Tris HCl, pH 7.5, 150 mM NaCl, and 2 mM CaCl_2_. Protein concentration was measured using a NanoDrop 2000c Spectrophotometer (Thermo Fisher Scientific), and purified proteins were analyzed on a 4–12% NuPAGE gel (cat. no. NP0321, Thermo Fisher Scientific) (Supplementary Figure 8).

### Fluorescence Polarization Assay

Fluorescence polarization (FP) assays were performed as described (Manoharan et al., 2019). The IC_50_ of compounds were determined in a binding/displacement assay using fluorescein-labeled PMCA peptide (derived from plasma membrane Ca^2+^ transporting ATPase, a CaM binding protein) as the probe and recombinant bovine calmodulin (cat. no. 208690, Merck), which has an amino acid sequence identical to the human isoform. The F-CaMKII peptide was used at 5 nM concentration with 50 nM of His-tagged wt and mutCaM. FP assays were carried out in a black low volume round bottom 384-well plate (cat. no. 4514, Corning) with a reaction volume of 20 μl. Compounds were threefold-diluted in an assay buffer (20 mM Tris Cl pH 7.5, 50 mM NaCl, 1 mM CaCl_2_, and 0.005% Tween 20), and a complex of 100 nM CaM and 10 nM F-PMCA peptide was added. The FP signals were recorded on the Clariostar (BMG labtech) plate reader with excitation at 482 ± 8 nm and emission at 530 ± 20 nm at 25°C, after 30–60-min interval for up to 5 h. Then the plate was incubated overnight at 4°C, and the next day, final readings were taken after a total of 24 h incubation. The fluorescence anisotropy was calculated and plotted against the logarithm of the compound concentration and fit to the log inhibitor vs. response–variable slope (four parameters) equation in Prism (GraphPad). The IC_50_ of the inhibitor was converted into *K*_d_ as described in Sinijarv et al. (2017) using the equation

$$\begin{array}{c} K_{d}=\frac{\text{[}I]{}_{50}}{1+\frac{\text{[}P]{}_{50}}{K_{D,probe}}+\frac{\text{[}E]{}_{0}}{K_{D,probe}}} \end{array}$$

where [I]_50_ is the concentration of the free inhibitor at 50% displacement, given as [*I*]_50_ = *IC*_50_−[*EI*]_50_, where [EI]_50_ is the concentration of the CaM:inhibitor complex in case of 50% displacement; [P]_50_ is the concentration of the free probe at 50% displacement; [E]_0_ is the concentration of free CaM at 0% displacement; and *K*_D__,probe_ is the dissociation constant of the complex of the probe and CaM. The *K*_D_ of the probe, F-PMCA to CaM, is 6 nM (Manoharan et al., 2019).

The potency of the irreversible covalent inhibitors was assessed as described in Singh et al. (2011). The potency and selectivity of a covalent inhibitor are governed by two parameters, namely, *K*_i_, the dissociation constant of the initial non-covalent complex, and *k*_2_, the rate of the subsequent covalent bond-forming reaction as given in the chemical equation

$$\begin{array}{c} E+I\begin{array}{c} K_{i}\\ ⇌ \end{array}E\cdot I\begin{array}{c} k_{2}\\ ⇌\\ k_{-2} \end{array}E-I \end{array}$$

E and I denote a protein target and its covalent inhibitor, respectively. E ⋅ I is the initial non-covalent complex, and E – I is the final covalent complex. To obtain the *K*_i_ and *k*_2_ rates, the fluorescence polarization signal after inhibitor treatment was plotted against the incubation time and fit using a one-phase decay function to obtain the observed rate constant, *k*_obs_. This was repeated for several inhibitor concentrations. Then, *k*_obs_ was plotted against the concentration of the inhibitor, and the data were fit to a hyperbolic equation $k_{obs}=\frac{k_{2}\times \left[I\right]}{K_{i}+\left[I\right]}$to obtain *K*_i_ and *k*_2_. The ratio of *k*_2_/*K*_i_ represents the second-order rate constant of the reaction of the covalent inhibitor with the target.

### Composite Drug Activity Score

The composite drug activity score was obtained by computing the activity of the compounds across various assays performed. The desired properties taken into consideration are a high activity in the spheroid assay, a higher selectivity for MDA-MB-231 over Hs 578T in the spheroid assay, a lower toxicity in the 2D assay against Hs 578T as compared to MDA-MB-231 cells, and a higher affinity to CaM. The final score is obtained using the equation below:

compositedrugactiviyscore=DSS(MDA-MB-231)2DSS(Hs 578T)×2Dtoxicity(Hs 578T)2Dtoxicity(MDA-MB-231)×1Kd

### BRET Assays

BRET assays were essentially performed as described by others (Lavoie et al., 2013; Bery et al., 2018). About 100,00–150,000 HEK293-EBNA (Meissner et al., 2001) cells were seeded per well of a 12-well plate in 1 ml of DMEM containing 10% FBS and 2 mM L-glutamine and were grown for 24 h. Next day, Rluc8-tagged donor and GFP2-tagged acceptor constructs were transfected into cells using a jetPRIME transfection reagent (cat. no. 114-75, Polyplus). Each well was transfected with about 1 μg of plasmid DNA using 3 μl of the jetPRIME reagent. For BRET donor saturation titration experiments, the concentration of donor plasmid (25 ng) was kept constant, and the concentration of acceptor plasmid was increased from 0 to 500 ng for RasG12V BRET pairs and 0–1,000 ng for K-Ras/CaM BRET pairs. The empty pcDNA3.1(-) plasmid was used to top-up the total DNA load per transfection. After 24 h of transfection, cells were treated with compounds or vehicle control (DMSO 0.2% v/v in a growth medium) at the specified concentration for 24 h or the stipulated time period in case of the time-course experiments. The cells from one well of a 12-well plate were collected, washed, and re-plated in PBS (cat. no. 14190-094, Gibco, Thermo Fisher Scientific) on flat bottom, white 96-well plates (cat. no. 236108, Nunc, Thermo Fisher Scientific) as four technical replicates containing 90 μl of cell suspension per well. Then fluorescence intensity followed by BRET readings were carried out on a Clariostar (BMG Labtech) plate reader at 25°C. The fluorescence intensity (RFU) of GFP2 was measured with excitation at 405 ± 10 nm and emission 515 ± 10 nm; it is proportional to the acceptor concentration [acceptor]. BRET readings were taken well by well by adding 10 μl of 100 μM coelenterazine 400a (cat. no. C-320, GoldBio), the Rluc8 substrate to each well (final concentration of 10 μM) using the injector present in the plate reader. Luminescence emission intensities were simultaneously recorded at 410 ± 40 nm (RLU, proportional to [donor]) and 515 ± 15 nm (BRET signal).

The raw BRET ratio was calculated as the BRET signal measured at 515 nm divided by the emission signal measured at 410 nm (RLU). The BRET ratio was obtained by subtracting the raw BRET ratio by a background BRET signal measured for cells expressing only the donor (Bacart et al., 2008) as indicated in the formula below:

BRETratio=λem 515nm(donor+acceptor)λem 410nm(donor+acceptor)-λem 515nm(donoronly)λem 410nm(donoronly)

with *donor+acceptor* denoting cells transfected with the BRET pair and *donor only* being cells expressing only the donor.

The expression of the acceptor relative to the donor ([acceptor]/[donor]) was determined as $relativeexpression=\frac{RFU}{RLU}$.

For BRET donor saturation titration experiments, the BRET ratio was plotted against the [acceptor]/[donor] ratio. Technical repeat data points were averaged, and data points from all biological repeats were collected into one graph for subsequent fitting. The BRET ratio vs. relative expression data were fitted using a binding saturation equation in the Prism (GraphPad) software to obtain BRET_max_ and BRET_50_ using the equation $y=\frac{BRET_{max}\times x}{BRET_{50}+x}$, where x is the relative expression and y is the BRET ratio. BRET_max_ represents the maximum saturation BRET signal and depends on the structural parameters (distance, orientation) of the BRET complex. BRET_50_ corresponds to the ratio of the acceptor construct over the donor construct required to attain 50% of the maximum BRET signal and is a measure of the effective relative affinity between the interacting BRET pair (Marullo and Bouvier, 2007).

When applying the DSS analysis to nanoclustering-BRET data, we used mevastatin (10 μM) to obtain the asymptote parameter (a) for the maximal inhibition effect, as it prevents the prenylation of Ras proteins, their plasma membrane trafficking, and therefore nanoclustering. Otherwise, normalized BRET ratio data were converted to % inhibition and then subsequently uploaded onto the Breeze site (see text footnote 1).

Using BRET donor saturation data, the A/D plasmid ratio at which the BRET ratio changes most linearly with the relative expression was determined for each BRET sensor and then used for testing compound treatments.

### ATARiS Gene Dependence Score

To generate the ATARiS sensitivity plots, Excel files corresponding to the normalized viability data for the siRNA knockdown of each gene of interest were downloaded from the publicly available database of the project DRIVE^2^ (McDonald et al., 2017). The Project DRIVE study is a large-scale RNAi screen in which 2D viability effects of mRNA knockdown were assessed (McDonald et al., 2017). The ATARiS algorithm was used in this study to aggregate consistent shRNA activity to gene level activity (Shao et al., 2013). From the Excel files of each gene of interest, the sensitivity score data were extracted, and a double gradient heatmap plot was generated using Prism (GraphPad). Higher gene dependence (of 2D viability) is indicated by a negative score, while scores zero or above represent no or neutral effects.

### Confocal Imaging

The localization of Ras and CaM fusion proteins was visualized by confocal microscopy. For imaging, MDCK cells were cultured in DMEM supplemented with 10% FBS and 2 mM L-glutamine at 37 °C with 5% CO_2_. Cells were seeded on glass coverslips 1.5H (cat. no. LH22.1, Carl Roth) in 6-well plates (cat. no. 657160, Cellstar, Greiner Bio-One), and plasmids were transiently transfected with jetPRIME. Cells were fixed 48 h after the transfection with 4% paraformaldehyde (cat. no. 43368, Alfa Aesar) in PBS for 10 min at ambient temperature. After washing with PBS-Tween 0.05% (cat. no. 9127.1, Carl Roth), DNA was stained with a 1 μg/ml solution of DAPI (cat. no. D1306, Thermo Fisher Scientific) diluted in PBS for 10 min. The coverslips were mounted onto glass slides using Vectashield (cat. no. H-1000, Vector Laboratories). Images were captured on a spinning disk confocal microscope (Andor, Oxford Instruments), fitted with a Zyla 5.5 sCMOS camera (Andor, Oxford Instruments), using a plan APO 60 × /1.40 Ph3 DM oil immersion objective (Nikon) and NIS-Elements Imaging Software (Nikon).

To evaluate the effect of compounds on centrosome numbers during mitosis, HeLa cells were seeded in 6-well plates onto sterile coverslips and cotransfected with 0.5 μg of pmCherry-CaM and 1.5 μg pEGFP-Centrin1 plasmids using 4 μl of jetPRIME. Twenty-four hours after the transfection, cells were synchronized with 60 ng/ml of nocodazole for 16 h. After the removal of nocodazole, the cells were treated with the protease inhibitor MG132 (10 μM) to block the cells in metaphase and either calmidazolium (20 μM), **1** (50 μM), or DMSO (0.5%) for 2 h. Cells were then fixed with 4% paraformaldehyde in PBS for 10 min at ambient temperature. After washing with PBS-Tween 0.05%, DNA was stained with a 1 μg/ml solution of DAPI diluted in PBS for 10 min. Coverslips were mounted on glass slides using Vectashield, and images were captured on a spinning disk confocal microscope. Images were analyzed with the ImageJ software, and the number of transfected mitotic cells with multipolar and normal bipolar phenotypes was counted (between 35 and 70 cells per test condition). The percentage multipolar vs. bipolar cells was computed to generate the plot using the Prism software.

### Data Analysis

All data analysis was performed using Prism (GraphPad) version 9 unless otherwise indicated. The number of independent biological repeats, n, for each data set is provided in the relevant figure legend. Unless otherwise stated, statistical significance was evaluated using one-way ANOVA. A *p*-value < 0.05 is considered statistically significant, and the statistical significance levels are annotated as follows: ^∗^*p* < 0.05; ^∗∗^*p* < 0.01; ^∗∗∗^*p* < 0.001; ^****^p < 0.0001, or ns = not significant.

## Data Availability Statement

The original contributions presented in the study are included in the article/Supplementary Material, further inquiries can be directed to the corresponding author/s.

## Author Contributions

SO designed and performed the 3D spheroid assay, 2D cell viability and toxicity studies, BRET assays, and DSS analysis, and performed the RT-qPCR experiments and cloning together with MC. GM developed the BRET assay, designed and performed FP assays, and performed cloning. AK synthesized the compounds and curated the analytical data. CL performed microscopy. MC together with GM implemented the gateway cloning system and performed cloning, protein purification, and RT-qPCR. FM contributed reagents and funding support. JY-K collaboratively designed the compounds with AK. SO and GM analyzed all the data. DA conceived the study, designed the experiments, interpreted the results, and wrote the manuscript together with SO, GM, AK, and JY-K. All authors commented on the manuscript.

## Conflict of Interest

FM was employed by company Leidos Biomedical Research, Inc. The remaining authors declare that the research was conducted in the absence of any commercial or financial relationships that could be construed as a potential conflict of interest.

## Footnotes

1 https://breeze.fimm.fi/

2 https://oncologynibr.shinyapps.io/drive/
