# Supplementary material for: A Covalent Calmodulin Inhibitor as a Tool to Study Cellular Mechanisms of K-Ras-Driven Stemness
Source: Front Cell Dev Biol. 2021 Jul 8;9:665673. doi: 10.3389/fcell.2021.665673 (PMC8296985; doi:10.3389/fcell.2021.665673)
Supplement: Supplementary file 1 [file Data_Sheet_1.PDF]

Supplementary Material

Data S1
Chemical Synthesis and Characterization

Table of Contents

1. Potential of covalent adduct formation with CaM p. 2
2. General information p. 5
3. Experimental procedures and characterization data p. 6
4. References p. 11
5. ^{1}H NMR and ^{13}C NMR Spectra p. 12-23

1. Potential for covalent adduct formation with CaM

The here described azulene-based inhibitors, such as compound **1** contain a built-in electrophilic *ortho*-quinone methide (*o*-QM) substructure (**Scheme S1, Top, in blue**). It can therefore be hypothesized that the *ε*-amino group of Lys 75 and/ or 77 and/ or 148 of CaM could attack the *o*-QM substructure of **1** in a nucleophilic aromatic substitution reaction (S_NAr). The occupied piperidinyl substituent (or another secondary amino functionality-containing substituent) is expelled as a nucleofugal leaving group and the covalent **CaM inhibitor (CaMi)-adduct 1** is formed (**Scheme S1, Top, dotted circle**), which in turn could cause irreversible inactivation of CaM.

This possibility is supported by a previously reported *model reaction*, where 2-pyrrolidinylbenzazulen-3-one **11** undergoes a rapid exchange reaction in the presence of another primary amine, such as *n*-$BuNH_2$ (**Scheme S1, Middle**). 2-*n*-Butylaminobenzazulenone **15** is formed rapidly even when the reaction is run at 0 °C, and the reaction proceeds in high yield without the need of any added catalyst or oxidant (conversion 91% in 60 min). We believe that a nucleophilic aromatic substitution reaction (S_NAr) could take place with Lys residues of CaM in a similar way.

Furthermore, adding the C1 formyl group to the benzazulene skeleton may contribute to increased affinity towards CaM as observed for e.g. **1**. In fact, this compound could show increased S_NAr reactivity due to its electron withdrawing formyl group, which is likely to make the C2 carbon more reactive. Moreover, the C1 formyl group could participate alone in the formation of a Schiff base with *ε*-amino group of Lys 75 and/ or 77 and/ or 148 leading to covalent **CaMi-adduct 2 (Scheme S1, Bottom)**. In this regard, the here described formyl benzazulenones could form an irreversible adduct, similar to what has been reported for Oph A (**Scheme S2**).

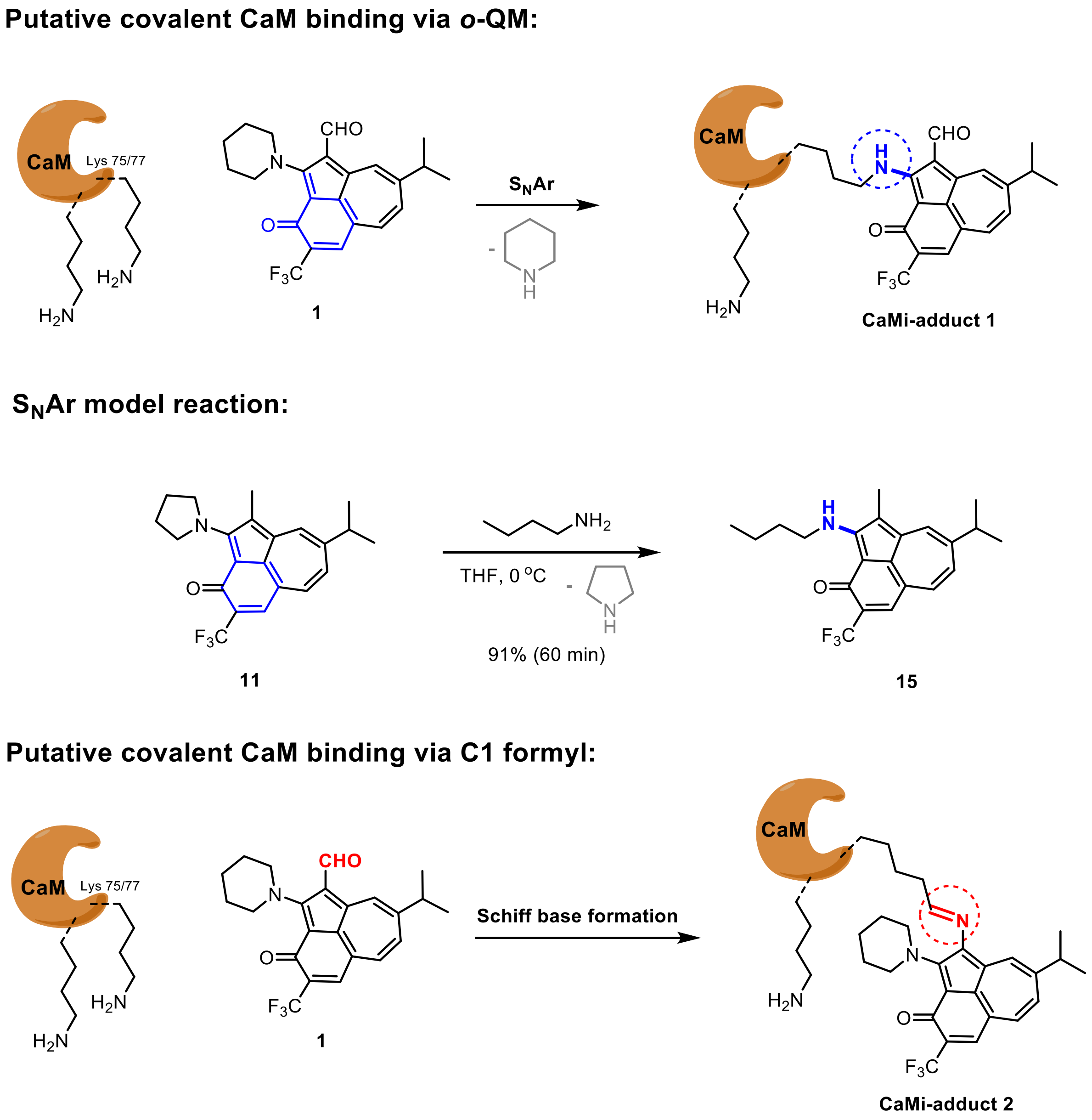

Scheme S1 – Putative covalent adduct formation of aminobenzazulenones with CaM. *Top*: Azulene-based CaMi **1** with an electrophilic *o*-QM (highlighted in blue) could form the covalent CaMi-adduct **1** via a nucleophilic aromatic substitution reaction (S$_N$Ar). *Middle*: Evidence for the S$_N$Ar mechanism was provided by the model reaction between **11** and *n*-Bu-NH$_2$ in a previous synthetic study.[s1] *Bottom*: Covalent Schiff-base formation by C1 formyl group of **1** with CaM leading to CaMi-adduct **2**.

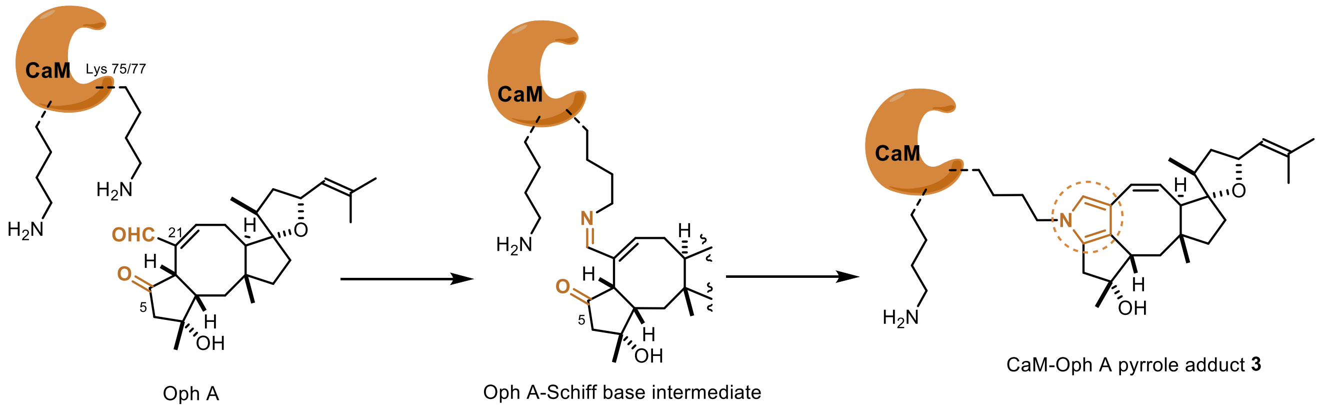

Scheme S2. **Oph A irreversibly inhibits CaM**. Schematic drawing of covalent reaction of Oph A with lysine residues of CaM. The irreversible pyrrole adduct **3** (dotted circle) is formed from the Schiff base intermediate via the Paal-Knorr reaction.

Synthesis of azulene-based CaMi

We used the previously reported 8-isopropyl-1-methyl-4-(trifluoromethyl)-*3H*-benzo[*cd*]azulen-3-one **16** as a starting compound for introducing the 2-amino substituents to the benzazulen-3-one scaffold.[s1] Due to its *o*-quinone methide (*o*-QM) moiety **16** undergoes a rapid initial Michael reaction (1,4-conjugate addition reaction) with the amino nucleophile to form a phenolic intermediate, which is not isolable, but is oxidized *in situ* restoring the aromatic azulene moiety. We then used a set of primary and secondary amines together with a gentle oxidant 1,4-benzoquinone under mild reaction conditions to synthesize a set of 2-aminobenzazulen-3-ones **8**–**14** in high yields (**Scheme S3**).

Given that ophiobolin A alkylates its macromolecular target via the formyl substituent on C7 (**Scheme S2**), we synthesized a set of additional formyl derivatives from the aminobenzazulen-3-ones by regioselective oxidation of the 3-methyl group to generate **1**–**7** (**Scheme S3**). Hence, the formyl derivatives have potentially two alkylating warheads. In summary, a total of fourteen 2-aminobenzazulenone derivatives were synthesized.

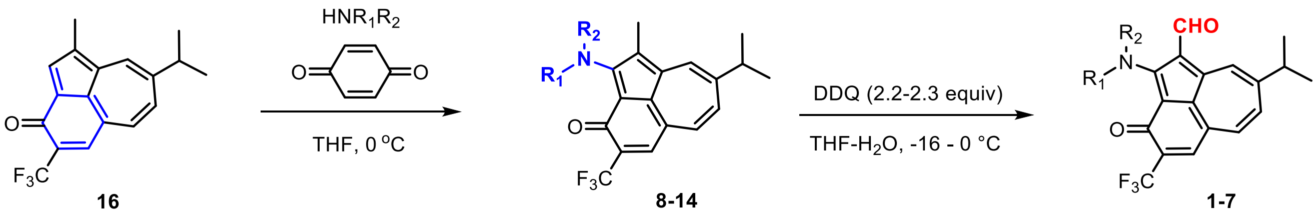

Scheme S3. Synthesis of 2-aminobenzazulenones from 16 and subsequent oxidation.

2. General information

All reactions were carried out using commercially available starting materials (Sigma-Aldrich, Schnelldorf, Germany; Fluka, Buchs, Switzerland and Alfa Aesar, Ward Hill, Massachusetts, USA) and solvents without further purification. Anhydrous solvents were purchased from Sigma-Aldrich. Column chromatography was performed with Merck 230−400 mesh silica gel or with an automated Biotage high performance flash chromatography Isolera One (Uppsala, Sweden) using a 0.1-mm path length flow cell UV-detector/recorder module (fixed wavelength: 254 nm). Analytical thin layer chromatography (TLC) was carried out using 0.2-mm silica gel plates (silica gel 60, F_{254}, Merck KGaA, Darmstadt, Germany). Nuclear magnetic resonance spectra (^1H NMR and ^{13}C NMR) were recorded on Bruker Ascent 400 (Bruker Corporation, Billerica, Massachusetts, USA). ^1H NMR at 400 MHz and ^{13}C NMR at 101 MHz. For $CDCl_3$ the chemical shifts are reported in parts per million (ppm) and on the δ scale using tetramethylsilane (TMS) as an internal reference. The coupling constants J are quoted in hertz (Hz). Data for ^1H NMR spectra are reported as follows: chemical shift (multiplicity, integration, coupling constant(s)). The multiplicity was abbreviated as follows: br = broad signal, s = singlet, d = doublet, t = triplet, q = quartet, quin = quintet, sep = septet, dd = doublet of doublets, dt = doublet of triplets, dq = doublet of quartets, m = multiplet, m_c = centered multiplet. High resolution mass spectra (HRMS) were measured on a Waters Synapt G2 (Waters Corporation, Milford, Massachusetts, USA) and reported for the molecular ions $[M+H]^+$ or $[M–H]^−$. LC-MS purity analyses were executed with Waters Acquity® UPLC system (Waters, Milford MA, USA) attached to Acquity PDA detector and Waters Synapt G2 HDMS mass spectrometer via an ESI ion source.

3. Experimental procedures and compound characterization data

8-Isopropyl-3-oxo-2-(piperidin-1-yl)-4-(trifluoromethyl)-3*H*-benzo[*cd*]azulene-1-carbaldehyde, 1.

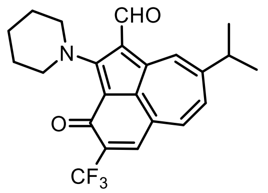

8-Isopropyl-1-methyl-2-(piperidin-1-yl)-4-(trifluoromethyl)-3*H*-benzo[*cd*]azulen-3-one **8** (39 mg, 0.10 mmol) was dissolved in a mixture of THF (5 mL) and H_2O (0.25 mL) and cooled to -16 °C (acetone-ice bath). Then, DDQ (52 mg, 0.23 mmol) in THF (2-3 mL) was added, and a rapid color change from dark red-gray to red-pink took place. The oxidation was finished in 30 min (monitored by tlc) and the reaction mixture was diluted with EtOAc (50 mL) and washed with a 1 M solution of NaOH in H_2O (2 × 50 mL). Organic layer is dried over anhydrous Na_2SO_4, filtered and evaporated, and the crude product was purified by automated chromatography (eluent: gradient of EtOAc–*n*-heptane) to give **1** as a magenta-colored solid (21 mg, 52%). ^{1}H NMR (400 MHz, C*D*Cl_3): δ 10.41 (s, 1H), 9.45 (d, J = 1.6 Hz, 1H), 8.06 (s, 1H), 7.88 (d, 1H, J = 10.8 Hz), 7.54 (dd, 1H, J = 10.8 Hz, 1.6 Hz), 3.90 (4H, m_c), 3.25 (sep, 1H, J = 6.8 Hz), 2.00–1.94 (4H, m), 1.87–1.80 (2H, m), 1.43 (d, 6H, ^3J = 6.8 Hz); ^{13}C NMR (101 MHz, C*D*Cl_3): δ 185.7, 172.9, 166.4, 163.7, 149.7, 141.7, 139.8 (q, J = 5 Hz), 139.5, 134.1 (q, J = 27 Hz), 131.1, 131.0, 128.1, 123.1 (q, J = 271 Hz), 117.1, 114.9, 56.8 (2), 40.4, 27.4 (2), 24.4 (2), 24.0; LC-MS: $[M+H]^+$, *m/z* 402 (t_r = 5.47 min); ≥95% (purity by ^{1}H-NMR). HRMS-ESI *m/z*: calc. for $C_{23}H_{23}F_3NO_2$ $[M+H]^+$: 402.1681, found 402.1684.

8-Isopropyl-2-(morpholin-1-yl)-3-oxo-4-(trifluoromethyl)-3*H*-benzo[*cd*]azulene-1-carbaldehyde, 2.

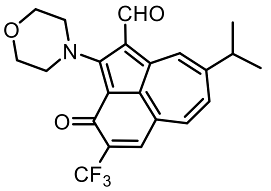

8-Isopropyl-1-methyl-2-(morpholin-1-yl)-4-(trifluoromethyl)-3*H*-benzo[*cd*]azulen-3-one **9** (0.11 g, 0.28 mmol) was dissolved in a mixture of THF (8 mL) and H_2O (0.41 mL) and cooled to -16 °C (acetone-ice bath). Then, DDQ (0.15 g, 0.65 mmol, 2.3 equiv) in THF (4 mL) was added, and a rapid color change from dark red-gray to red-pink took place. The oxidation was finished in 30 min (monitored by tlc) and the reaction mixture was diluted with EtOAc (100 mL) and washed with a 1 M solution of NaOH in H_2O (2 × 50 mL). Organic layer is dried over anhydrous Na_2SO_4, filtered and evaporated, and the crude product was purified by automated chromatography (eluent: gradient of EtOAc–*n*-heptane) to give **2** as a magenta-colored solid (61 mg, 54%). ^{1}H NMR (400 MHz, C*D*Cl_3): δ 10.46 (s, 1H), 9.40 (d, J = 1.2 Hz, 1H), 8.10 (s, 1H), 7.9 (d, 1H, J = 10.4 Hz), 7.62 (dd, 1H, J = 10.4 Hz, 1.2 Hz), 4.08–4.06 (4H, m), 3.98–3.96 (4H, m), 3.28 (sep, 1H, J = 6.8 Hz), 1.45 (d, 6H, ^3J = 6.8 Hz); ^{13}C NMR (101 MHz, C*D*Cl_3): δ 185.0, 173.0, 165.4, 163.8, 150.0, 141.4, 141.1 (q, J = 6 Hz), 139.7, 134.3 (q, J = 28 Hz), 131.8, 131.3, 128.6, 123.0 (q, J = 272 Hz), 116.9, 114.5, 68.0(2), 55.5 (2), 40.5, 24.4 (2); LC-MS:

$[M+H]^+$, *m/z* 426; ≥95% (purity by ^{1}H-NMR). HRMS-ESI *m/z*: calc. for $C_{22}H_{290}F_3NO_3$ $[M+H]^+$: 426.1293, found 426.1293.

8-Isopropyl-2-(4-methylpiperazin-1-yl)-3-oxo-4-(trifluoromethyl)-3*H*-benzo[*cd*]-azulene-1-carbaldehyde, 3.

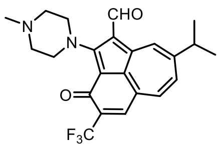

8-Isopropyl-1-methyl-2-(4-methyl-1-piperazin-1-yl)-4-(trifluoromethyl)-3*H*-benzo[*cd*]azulen-3-one **10** (39 mg, 0.10 mmol) was dissolved in a mixture of THF (5 mL) and H_2O (0.25 mL) and cooled to -16 °C (acetone-ice bath). Then, DDQ (52 mg, 0.23 mmol) in THF (2-3 mL) was added, and a rapid color change from dark red-gray to red-pink took place. The oxidation was finished in 30 min (monitored by tlc) and the reaction mixture was diluted with EtOAc (50 mL) and washed with a 1 M solution of NaOH in H_2O (2 × 50 mL). Organic layer was dried over anhydrous Na_2SO_4, filtered and evaporated, and the crude product was purified by automated chromatography (eluent: gradient of EtOAc–*n*-heptane) to give **3** as a magenta-colored solid (21 mg, 52%). ^{1}H NMR (400 MHz, C*D*Cl_3): δ 10.41 (s, 1H), 9.45 (d, J = 1.6 Hz, 1H), 8.06 (s, 1H), 7.88 (d, 1H, J = 10.8 Hz), 7.54 (dd, 1H, J = 10.8 Hz, 1.6 Hz), 3.90 (4H, m_c), 3.25 (sep, 1H, J = 6.8 Hz), 2.00–1.94 (4H, m), 1.87–1.80 (2H, m), 1.43 (d, 6H, ^3J = 6.8 Hz); ^{13}C NMR (75 MHz, C*D*Cl_3): δ 185.7, 172.9, 166.4, 163.7, 149.7, 141.7, 139.8 (q, J = 5 Hz), 139.5, 134.1 (q, J = 27 Hz), 131.1, 131.0, 128.1, 123.1 (q, J = 271 Hz), 117.10, 114.9, 56.8 (2), 40.4, 27.4 (2), 24.4 (2), 24.0; LC-MS: $[M+H]^+$, *m/z* 402 (t_r = 5.47 min) ≥95%; HRMS-ESI *m/z*: calc. for $C_{23}H_{23}F_3NO_2$ $[M+H]^+$: 402.1681, found 402.1684.

8-Isopropyl-3-oxo-2-(pyrrolidin-1-yl)-4-(trifluoromethyl)-3*H*-benzo[*cd*]azulene-1-carbaldehyde, 4 was prepared as described previously.[S3]

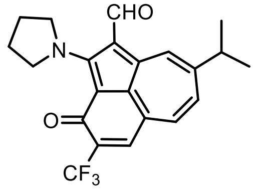

2-(Azetidin-1-yl)-8-isopropyl-3-oxo-4-(trifluoromethyl)-3*H*-benzo[*cd*]azulene-1-carbaldehyde, 5.

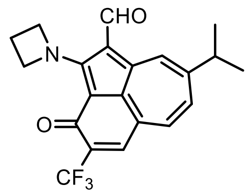

2-(Azetidin-1-yl)-8-isopropyl-1-methyl-4-(trifluoromethyl)-3*H*-benzo[*cd*]azulen-3-one **12** (23 mg, 0.064 mmol) was dissolved in a mixture of THF (6 mL) and H_2O (0.30 mL) and cooled to 0 °C (ice-water bath). Then, DDQ (32 mg, 0.14 mmol, 2.2 equiv) in THF (1 mL) was added, and a rapid color change from dark red-gray to bright red-pink took place. The oxidation was finished in 30 min (monitored by tlc) and the reaction mixture

was diluted with EtOAc (50 mL) and washed with a 1 M solution of NaOH in H_2O (3 × 30 mL). Organic layer was dried over anhydrous Na_2SO_4, filtered and evaporated, and the crude product was purified by automated chromatography (eluent: gradient of EtOAc–*n*-heptane) to give **5** as a magenta-red solid (20 mg, 84%). ^{1}H NMR (400 MHz, C*D*Cl$_3$): δ 10.15 (s, 1H), 9.40 (d, *J* = 1.6 Hz, 1H), 7.98 (s, 1H), 7.80 (d, 1H, *J* = 10.8 Hz), 7.44 (dd, 1H, *J* = 10.8 Hz, 1.6 Hz), 5.04 (2H, br s), 4.79 (2H, br s), 3.21 (sep, 1H, *J* = 6.8 Hz), 2.50 (2H, m_c), 1.41 (d, 6H, ^3J = 6.8 Hz); ^{13}C NMR (101 MHz, C*D*Cl$_3$): δ 183.5, 173.0, 164.4, 150.2, 141.5, 140.2 (q, *J* = 5 Hz), 139.8, 133.1 (q, *J* = 28 Hz), 130.2, 130.0, 127.1, 122.9 (q, *J* = 272 Hz), 116.1, 112.5, 61.2, 59.7, 40.4, 24.2 (2), 16.6; LC-MS: $[M+H]^+$, *m/z* 374 (t_r = 4.98 min) ≥95%; HRMS-ESI *m/z*: calc. for $C_{21}H_{19}F_3NO_2$ $[M+H]^+$: 374.1368, found 374.1368

2-[(2-Hydroxyethyl)amino]-8-isopropyl-3-oxo-4-(trifluoromethyl)-3*H*-benzo[*cd*]azulene-1-carbaldehyde, 6 was prepared as described previously.[S2]

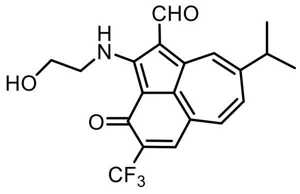

2-Amino-8-isopropyl-3-oxo-1-4-(trifluoromethyl)-3*H*-benzo[*cd*]azulene-1-carbaldehyde, **7** was prepared as described previously.[S2]

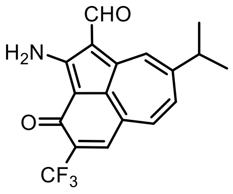

8-Isopropyl-1-methyl-2-(piperidin-1-yl)-4-(trifluoromethyl)-3*H*-benzo[*cd*]azulen-3-one, 8 was prepared as described previously.[S1]

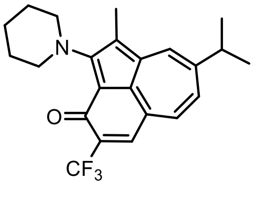

8-Isopropyl-1-methyl-2-(morpholin-1-yl)-4-(trifluoromethyl)-3*H*-benzo[*cd*]azulen-3-one, 9 was prepared as described previously.[S1]

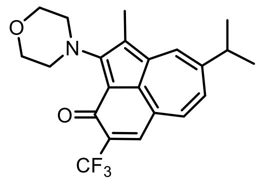

8-Isopropyl-1-methyl-2-(4-methylpiperazin-1-yl)-4-(trifluoromethyl)-3*H*-benzo[*cd*]azulen-3-one, 10.

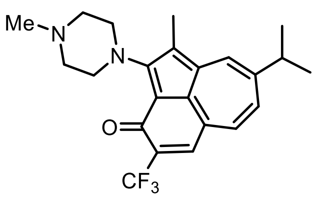

8-Isopropyl-1-methyl-4-(trifluoromethyl)-3*H*-benzo[*cd*]azulen-3-one **16** (0.10 g, 0.33 mmol) was dissolved in THF (6 mL) in a round-bottomed flask and cooled to 0 °C, followed by addition of *N*-methylpiperazine (0.111 mL, 1.00 mmol, 3.0 equiv) and 1,4-benzoquinone (36 mg, 0.33 mmol) in THF (1 mL). The reaction mixture was stirred at 0 °C and monitored by tlc until all **16** was consumed (3 h). Then the reaction mixture was diluted with EtOAc (80 mL) and washed with a 1 M solution of NaOH in H_2O (2 × 50 mL). Organic layer was dried over anhydrous Na_2SO_4, filtered and evaporated, and the crude product was purified by automated chromatography using a gradient of EtOAc-*n*-heptane to give **10** as a dark solid (84 mg, 63%). ^{1}H NMR (400 MHz, $CDCl_3$): δ 7.87 (s, 1H), 7.75 (d, 1H, J = 1.6 Hz), 7.47 (d, 1H, J = 11.2 Hz), 7.23 (dd, 1H, J = 1.6 Hz, J = 11.2 Hz), 3.91 (m_c, 4H), 3.11 (sep, 1H, J = 6.9 Hz), 2.79 (4H, m_c), 2.47 (s, 3H), 2.42 (3H, s), 1.38 (d, 6H, J = 6.9 Hz) ppm; ^{13}C NMR (101 MHz, $CDCl_3$): δ 172.4, 164.5, 154.7, 146.7, 140.1 (q, J = 5 Hz), 138.7, 134.8, 132.2 (q, J = 28 Hz), 128.8, 126.0, 124.2, 123.5 (q, J = 271 Hz), 115.4, 114.0, 56.1 (2), 52.4 (2), 46.2, 40.1, 24.2 (2), 12.8 ppm; LC-MS: $[M+H]^+$, m/z 403 (t_r = 3.46 min) ≥95%; HRMS-ESI m/z: calc. for $C_{23}H_{26}F_3N_2O$ $[M+H]^+$: 403.1997, found 403.1998.

8-Isopropyl-1-methyl-2-(pyrrolidin-1-yl)-4-(trifluoromethyl)-3*H*-benzo[*cd*]azulen-3-one, 11 was prepared as described previously.[S1]

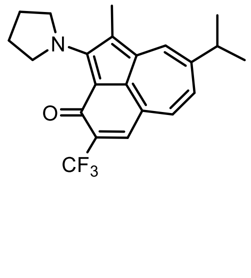

2-(Azetidin-1-yl)-8-isopropyl-1-methyl-4-(trifluoromethyl)-3*H*-benzo[*cd*]azulen-3-one, 12.

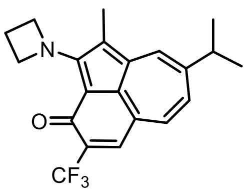

8-Isopropyl-1-methyl-4-(trifluoromethyl)-3*H*-benzo[*cd*]azulen-3-one **16** (0.060 g, 0.20 mmol) and 1,4-benzoquinone (0.20 mmol, 22 mg) were dissolved in THF (6 mL) in a round-bottomed flask and cooled to 0 °C. Then, a solution of azetidine hydrochloride (55 mg, 0.60 mmol, 3.0 equiv) in a mixture of H_2O-DMF (1:2, 3 mL) and triethylamine (0.14 mL, 1.0 mmol, 5 equiv) was added to the reaction mixture. The reaction mixture was stirred at 0 °C and after a 2-h reaction it was monitored by tlc showing a formation of product and unreacted starting material. Therefore, more azetidine hydrochloride (55 mg, 0.60 mmol, 3.0 equiv) in a mixture of H_2O-DMF (1:2, 3 mL) and triethylamine (0.14 mL, 1.0 mmol, 5 equiv) was added and the reaction was continued for additional 2 h. Then the reaction mixture was diluted with EtOAc (80 mL) and washed with a 1 M solution of NaOH in H_2O (2 × 50 mL). Organic layer was dried over anhydrous Na_2SO_4, filtered and evaporated, and the crude product was purified by automated chromatography using a gradient of EtOAc-*n*-heptane to give **12** as a dark-green solid (37 mg, 52%). ^{1}H NMR (400 MHz, C*D*Cl_3): δ 7.69 (s, 1H), 7.36 (d, 1H, J = 1.6 Hz), 7.17 (d, 1H, J = 11.2 Hz), 6.89 (dd, 1H, J = 1.6 Hz, J = 11.2 Hz), 5.02 (br s, 4 H), 2.97 (sep, 1H, J = 6.9 Hz), 2.40 (2H, m_c), 2.33 (3H, s), 1.32 (d, 6H, J = 6.9 Hz) ppm; ^{13}C NMR (101 MHz, C*D*Cl_3): δ 172.9, 163.3, 153.4, 145.5, 140.8 (q, J = 5 Hz), 140.0, 134.1, 131.1 (q, J = 28 Hz), 126.9, 125.1, 123.7 (q, J = 271 Hz), 113.9, 112.0, 56.1 (2), 58.6 (2), 39.9, 24.1 (2), 16.6, 10.9 ppm; LC-MS: $[M+H]^+$, *m/z* 360 (t_r = 6.05 min); ≥95% (purity by ^{1}H-NMR; HRMS-ESI *m/z*: calc. for $C_{21}H_{21}F_3NO$ $[M+H]^+$: 360.1575, found 360.1576.

2-[(2-Hydroxyethyl)amino]-8-isopropyl-1-methyl-4-(trifluoromethyl)-3*H*-benzo[*cd*]azulen-3-one, 13 was prepared as described previously.[S2]

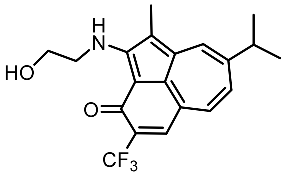

2-Amino-8-isopropyl-1-methyl-4-(trifluoromethyl)-3*H*-benzo[*cd*]azulen-3-one, 14 was prepared as described previously.[S1]

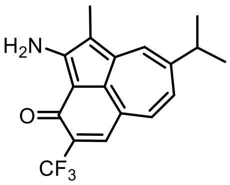

8-Isopropyl-1-methyl-4-(trifluoromethyl)-3*H*-benzo[*cd*]azulen-3-one, 16 was prepared as described previously.[S1]

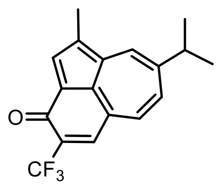

4. References

S1 Kiriazis, A.; Aumüller, I. B.; Arnaudova, R.; Brito, V.; Rüffer, T.; Lang, H.; Silvestre, S. M.; Koskinen, P.J.; Yli-Kauhaluoma, J. Nucleophilic Substitution of Hydrogen Facilitated by Quinone Methide Moieties in Benzo[*cd*]azulen-3-ones. *Org. Lett.* **2017**, *19*, 2030−2033.

S2 Kiriazis, A.; Johansson, N. G.; Vidilaseris, K.; Dreano, L.; Turku, A.; Khattab, A.; Leino, T. O.; Arnaudova, R.; Meri, S.; Goldman, A.; Yli-Kauhaluoma, J.; Xhaard, H. Discovery of Azulene Scaffold as Novel Inhibitor of Membrane-bound Pyrophosphatases of *Thermotoga maritima* (TmPPase). Manuscript.

S3 Cruz, C. D.; Arnaudova, R.; Aumüller, I. B.; Mäkkylä, H.; Yli-Kauhaluoma, J.; Kiriazis, A.; Tammela, P. Synthesis and Biological Evaluation of Tricyclic Azulene Derivatives as Potential Antibacterial Agents. Manuscript (submitted).

5. ^{1}H NMR and ^{13}C NMR Spectra

The following pages show NMR Spectra of indicated compounds.

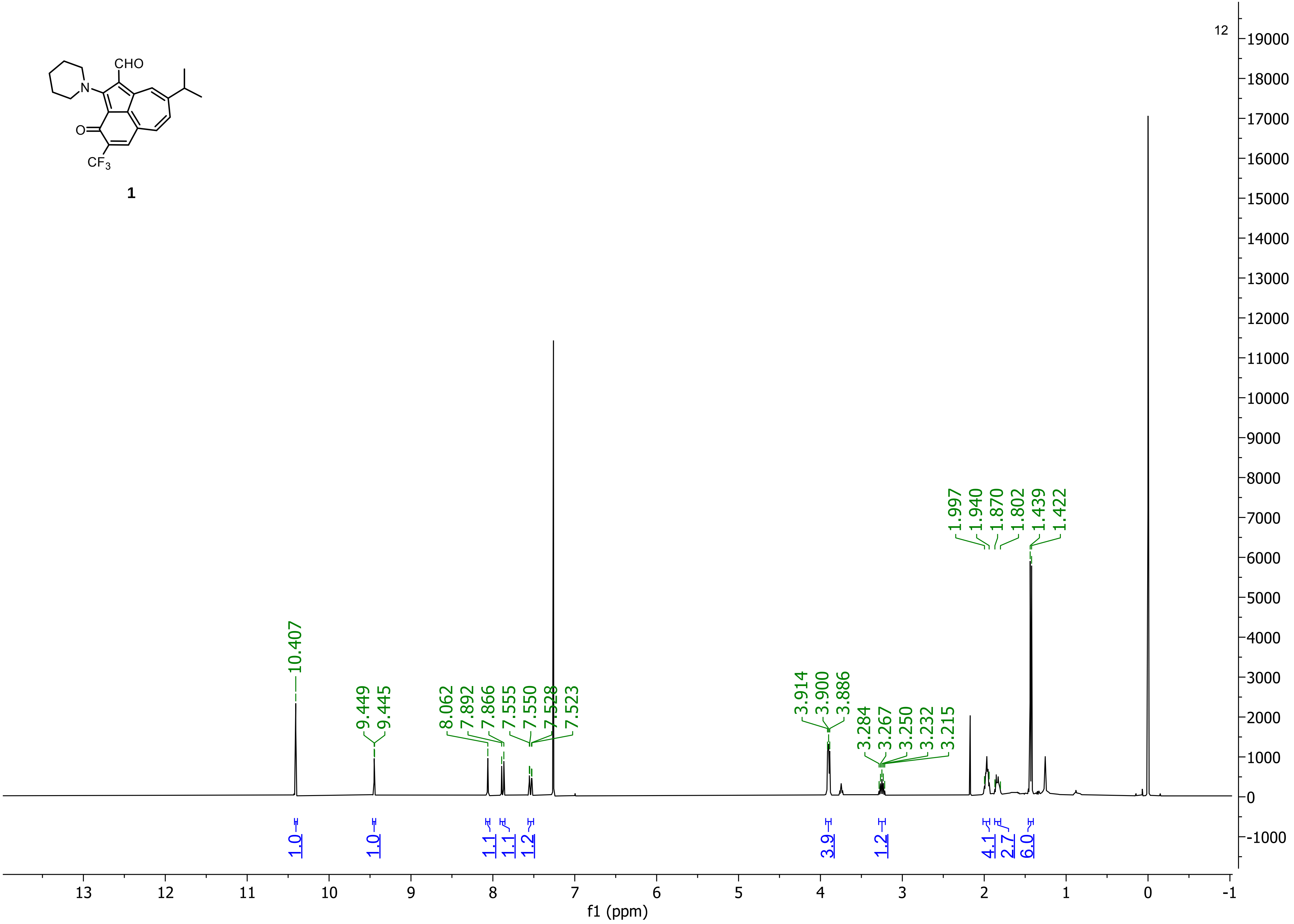
CHO
N
O
CF3
1
10.407
9.449
9.445
8.062
7.892
7.866
7.555
7.550
7.528
7.523
3.914
3.900
3.886
3.284
3.267
3.250
3.232
3.215
1.997
1.940
1.870
1.802
1.439
1.422
1.0
1.0
1.1
1.1
1.2
3.9
1.2
4.1
2.7
6.0
f1 (ppm)

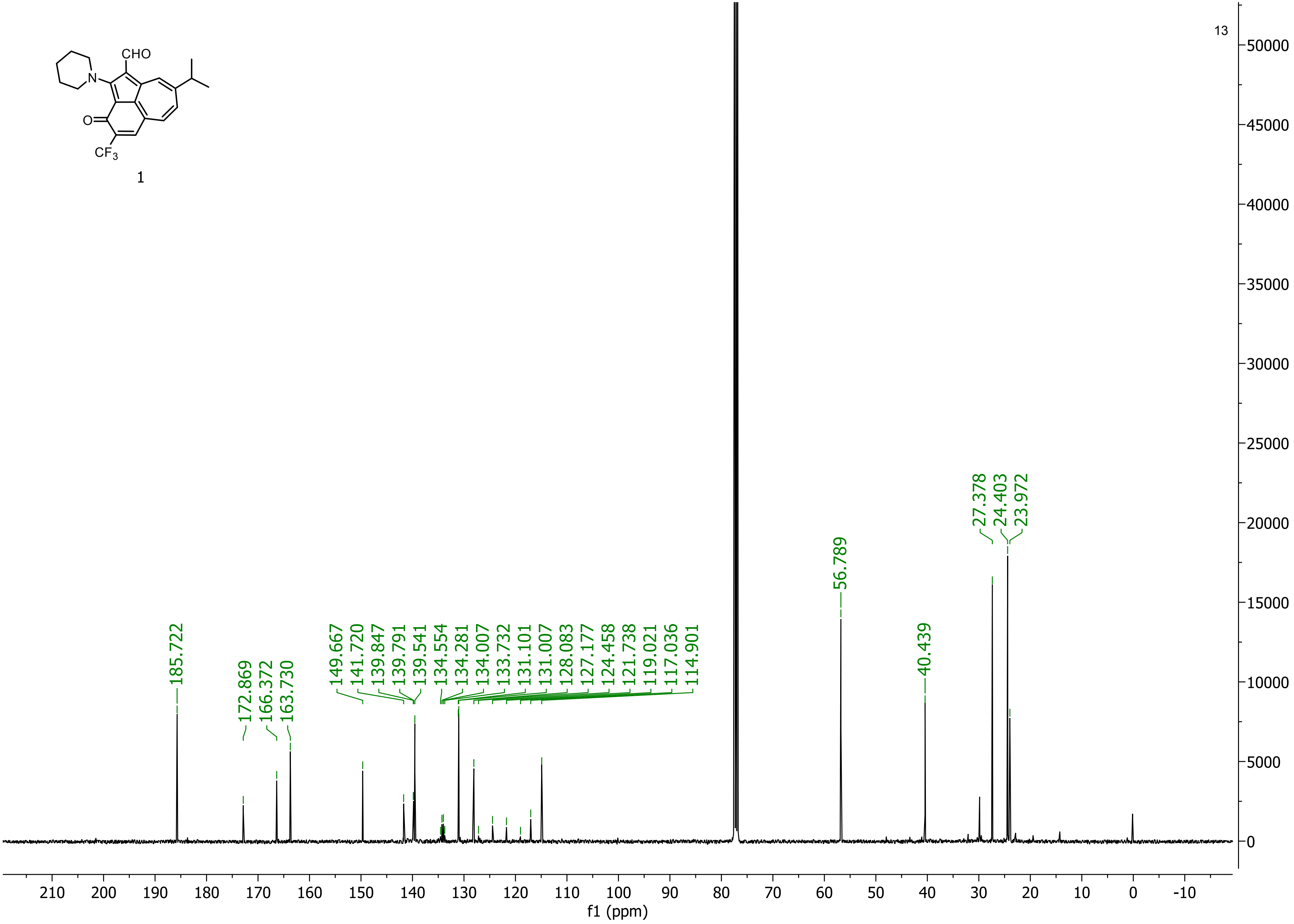
CHO
N
O
CF3
1
185.722
172.869
166.372
163.730
149.667
141.720
139.847
139.791
139.541
134.554
134.281
134.007
133.732
131.101
131.007
128.083
127.177
124.458
121.738
119.021
117.036
114.901
56.789
40.439
27.378
24.403
23.972
50000
45000
40000
35000
30000
25000
20000
15000
10000
5000
0
210 200 190 180 170 160 150 140 130 120 110 100 90 80 70 60 50 40 30 20 10 0 -10
f1 (ppm)

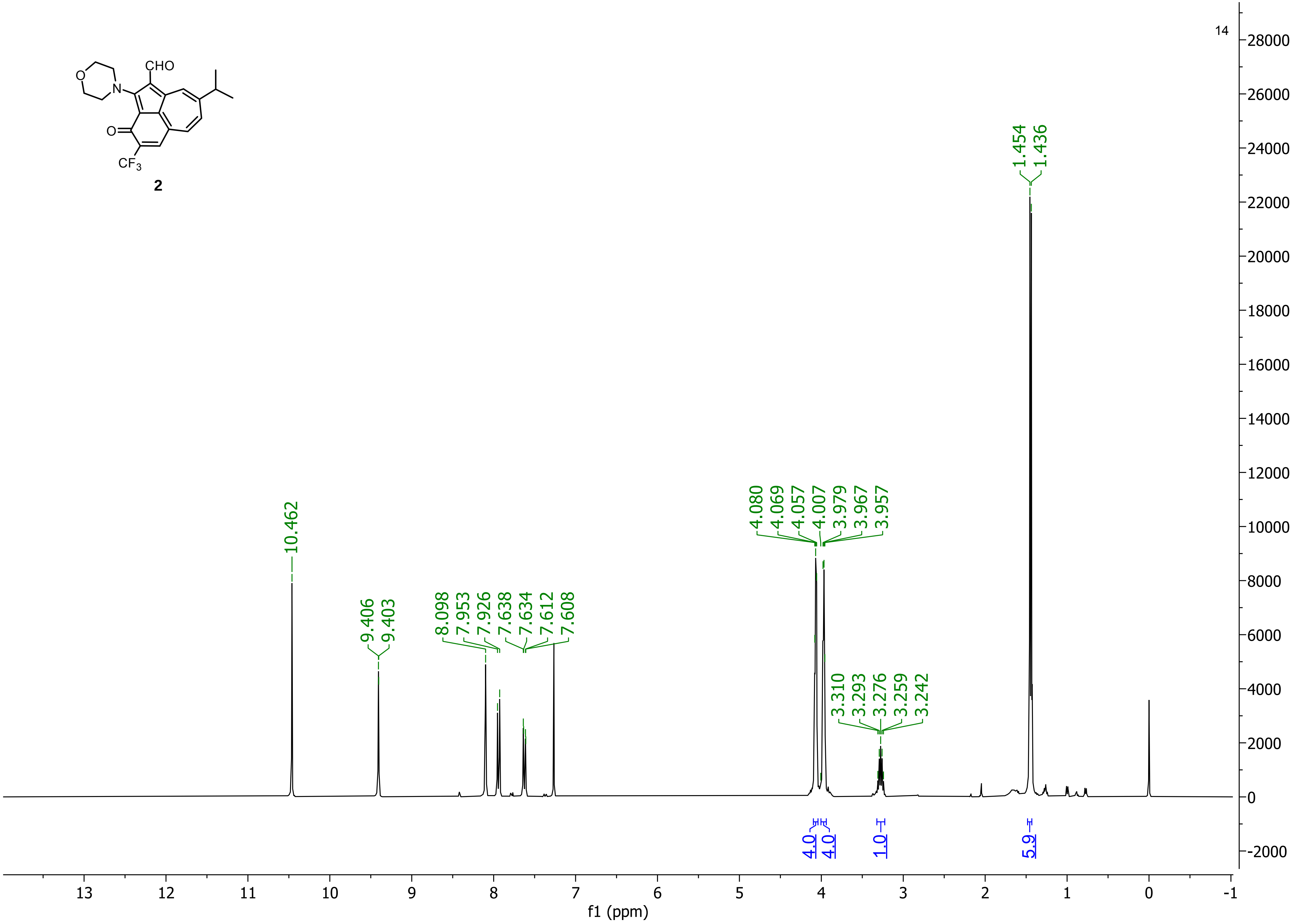
CHO
O
N
O
CF3
2
10.462
9.406
9.403
8.098
7.953
7.926
7.638
7.634
7.612
7.608
4.080
4.069
4.057
4.007
3.979
3.967
3.957
3.310
3.293
3.276
3.259
3.242
1.454
1.436
4.0
4.0
1.0
5.9
f1 (ppm)

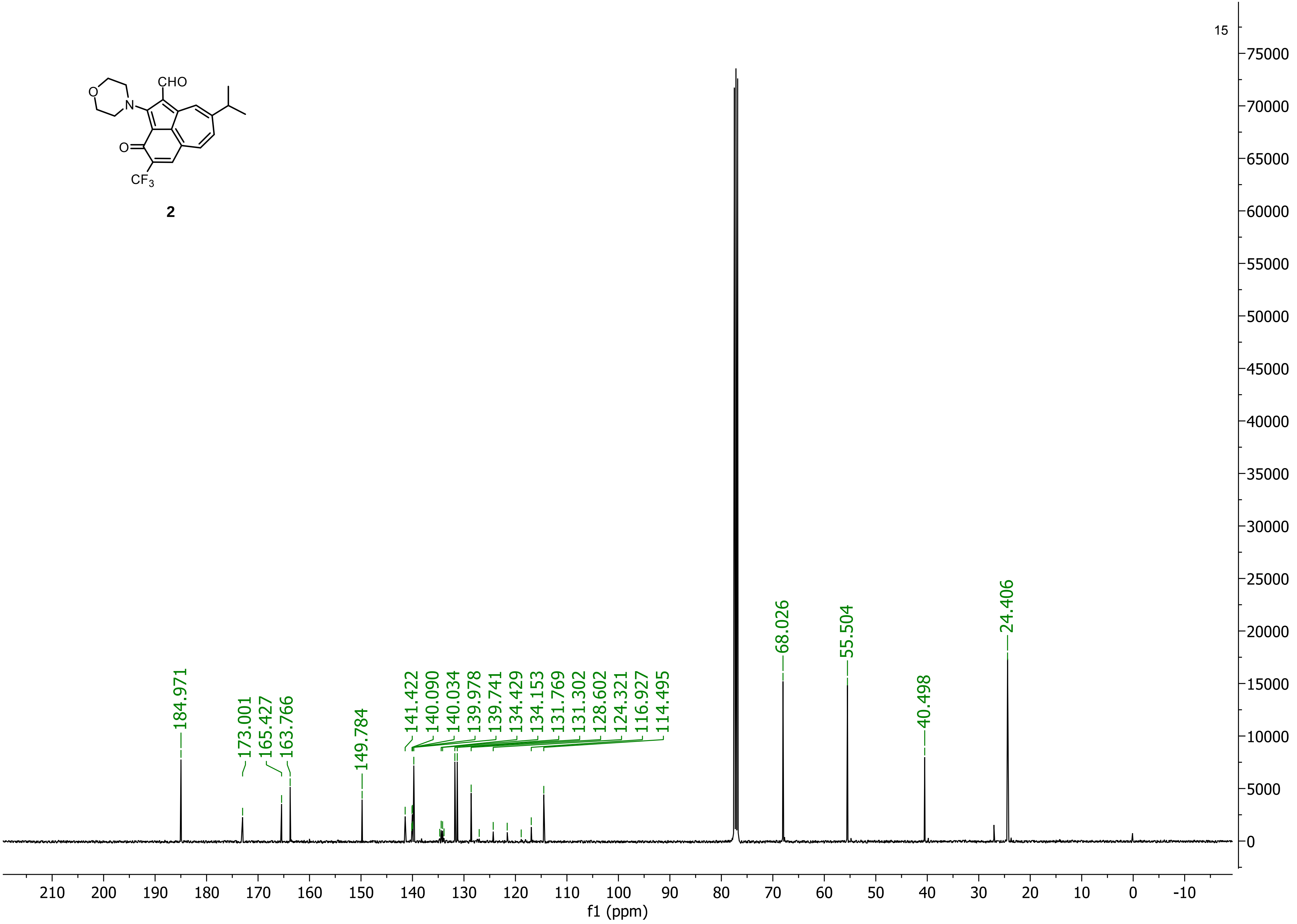
CHO
O
N
O
CF_3
2
184.971
173.001
165.427
163.766
149.784
141.422
140.090
140.034
139.978
139.741
134.429
134.153
131.769
131.302
128.602
124.321
116.927
114.495
68.026
55.504
40.498
24.406
75000
70000
65000
60000
55000
50000
45000
40000
35000
30000
25000
20000
15000
10000
5000
0
210 200 190 180 170 160 150 140 130 120 110 100 90 80 70 60 50 40 30 20 10 0 -10
f1 (ppm)

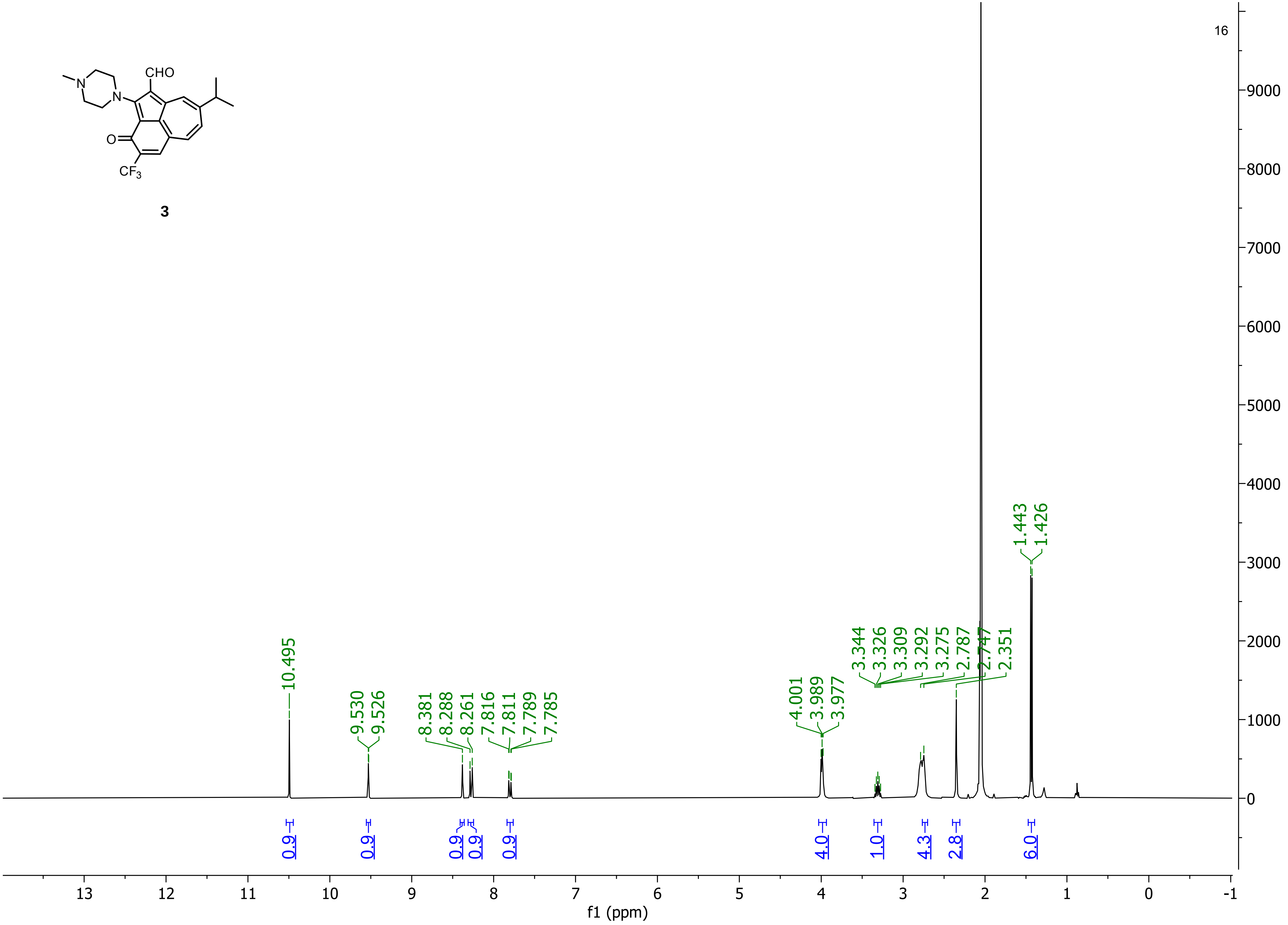
CHO
N
N
O
CF3
3
10.495
9.530
9.526
8.381
8.288
8.261
7.816
7.811
7.789
7.785
4.001
3.989
3.977
3.344
3.326
3.309
3.292
3.275
2.787
2.747
2.351
1.443
1.426
0.9
0.9
0.9
0.9
0.9
4.0
1.0
4.3
2.8
6.0
f1 (ppm)

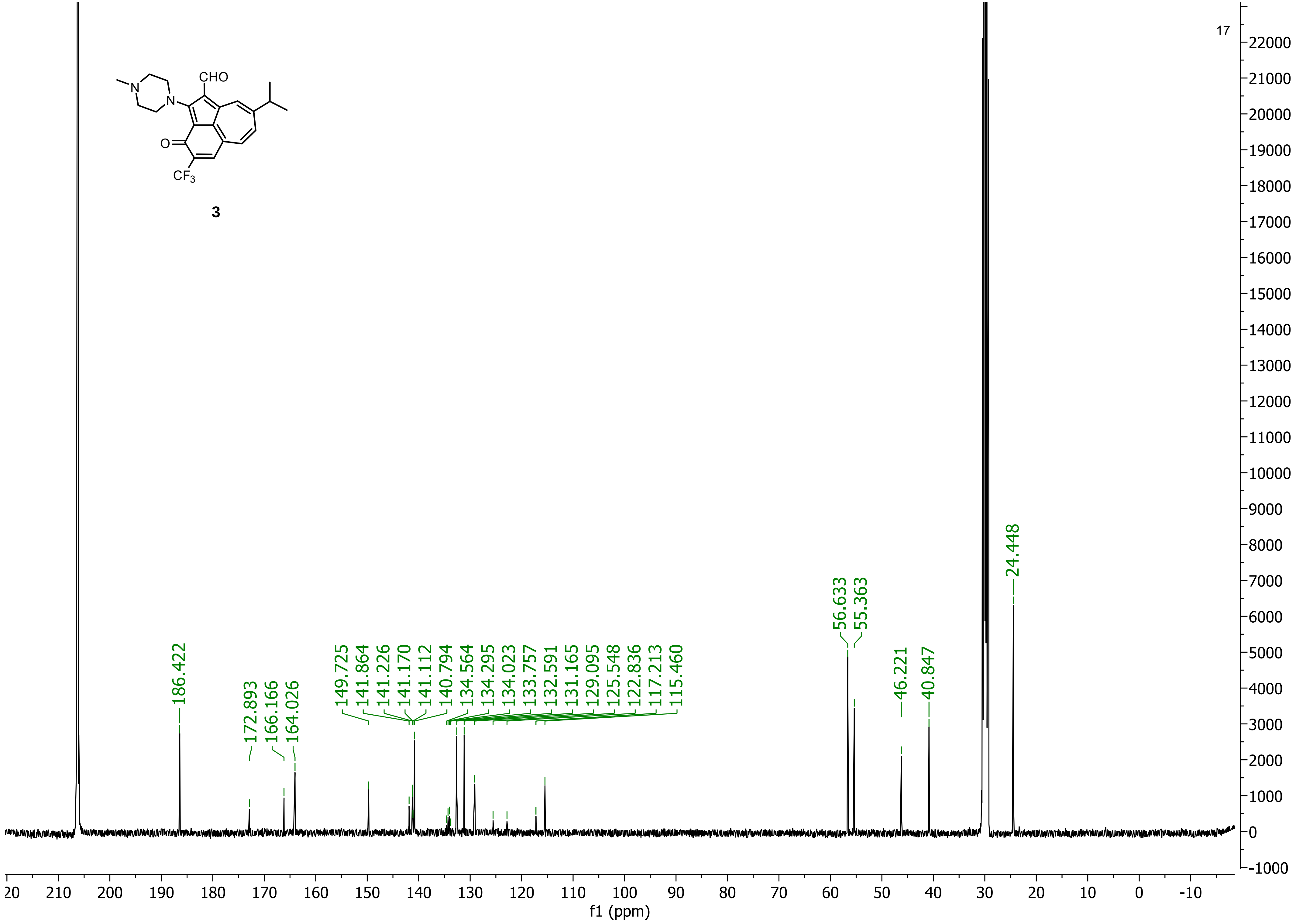
CHO
N
N
O
CF3
3
186.422
172.893
166.166
164.026
149.725
141.864
141.226
141.170
141.112
140.794
134.564
134.295
134.023
133.757
132.591
131.165
129.095
125.548
122.836
117.213
115.460
56.633
55.363
46.221
40.847
24.448
f1 (ppm)

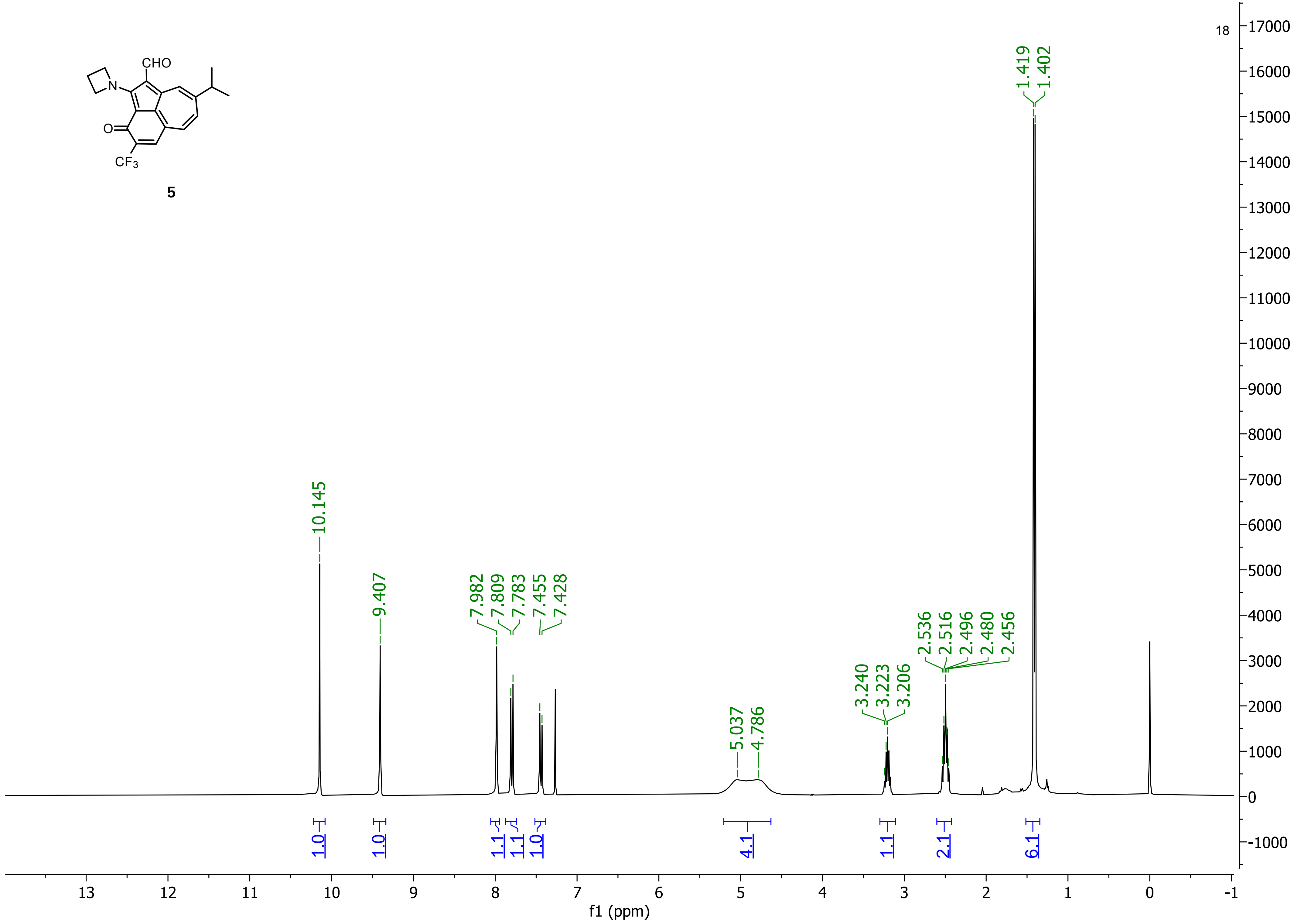
CHO
N
O
CF3
5
10.145
9.407
7.982
7.809
7.783
7.455
7.428
5.037
4.786
3.240
3.223
3.206
2.536
2.516
2.496
2.480
2.456
1.419
1.402
1.0
1.0
1.1
1.1
1.0
4.1
1.1
2.1
6.1
17000
16000
15000
14000
13000
12000
11000
10000
9000
8000
7000
6000
5000
4000
3000
2000
1000
0
-1000
13 12 11 10 9 8 7 6 5 4 3 2 1 0 -1
f1 (ppm)

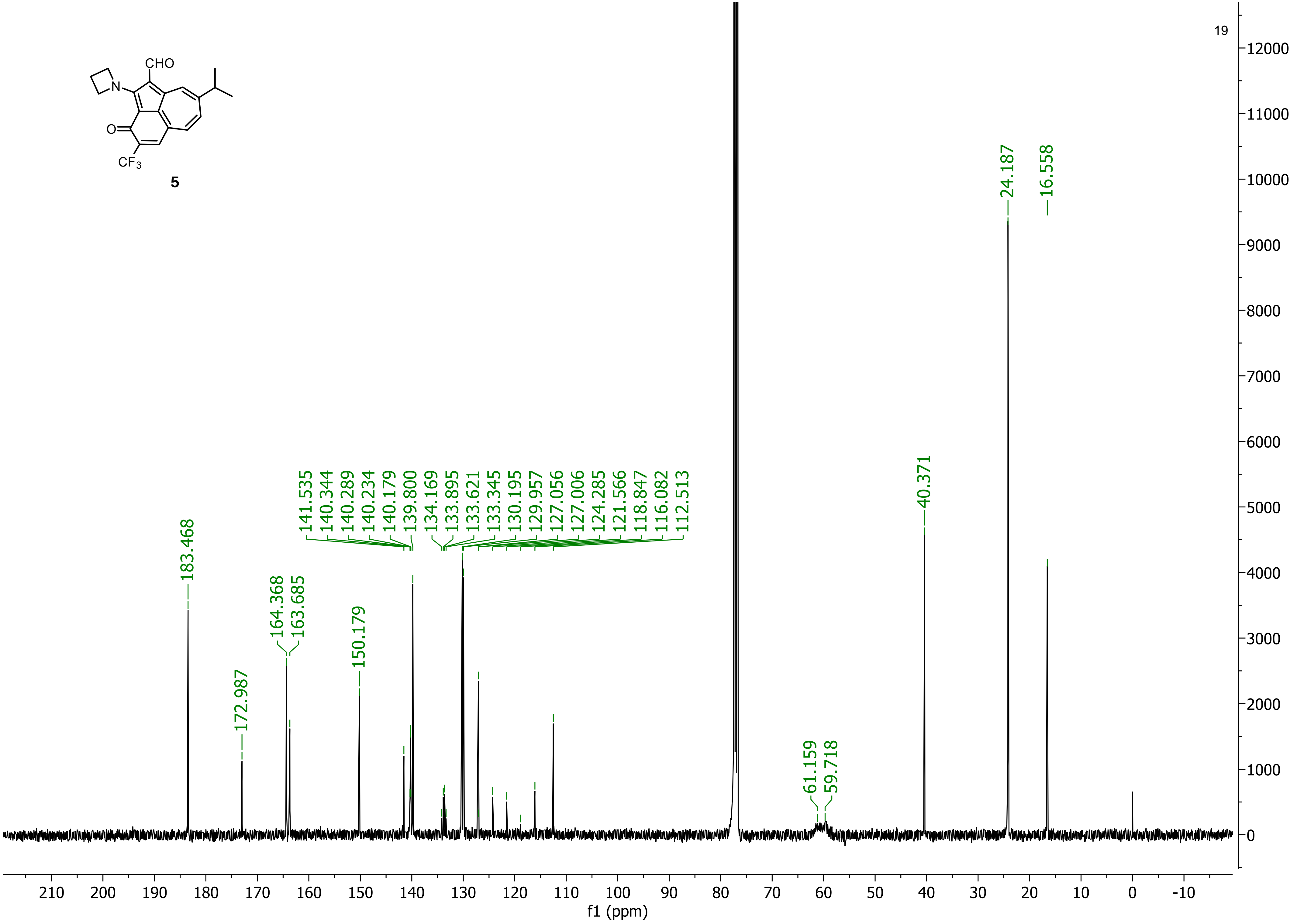
CHO
N
O
CF3
5
183.468
172.987
164.368
163.685
150.179
141.535
140.344
140.289
140.234
140.179
139.800
134.169
133.895
133.621
133.345
130.195
129.957
127.056
127.006
124.285
121.566
118.847
116.082
112.513
61.159
59.718
40.371
24.187
16.558
f1 (ppm)

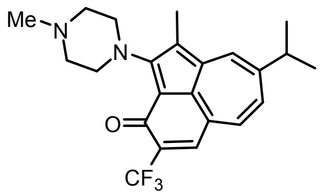
Me
N
N
O
CF3

10

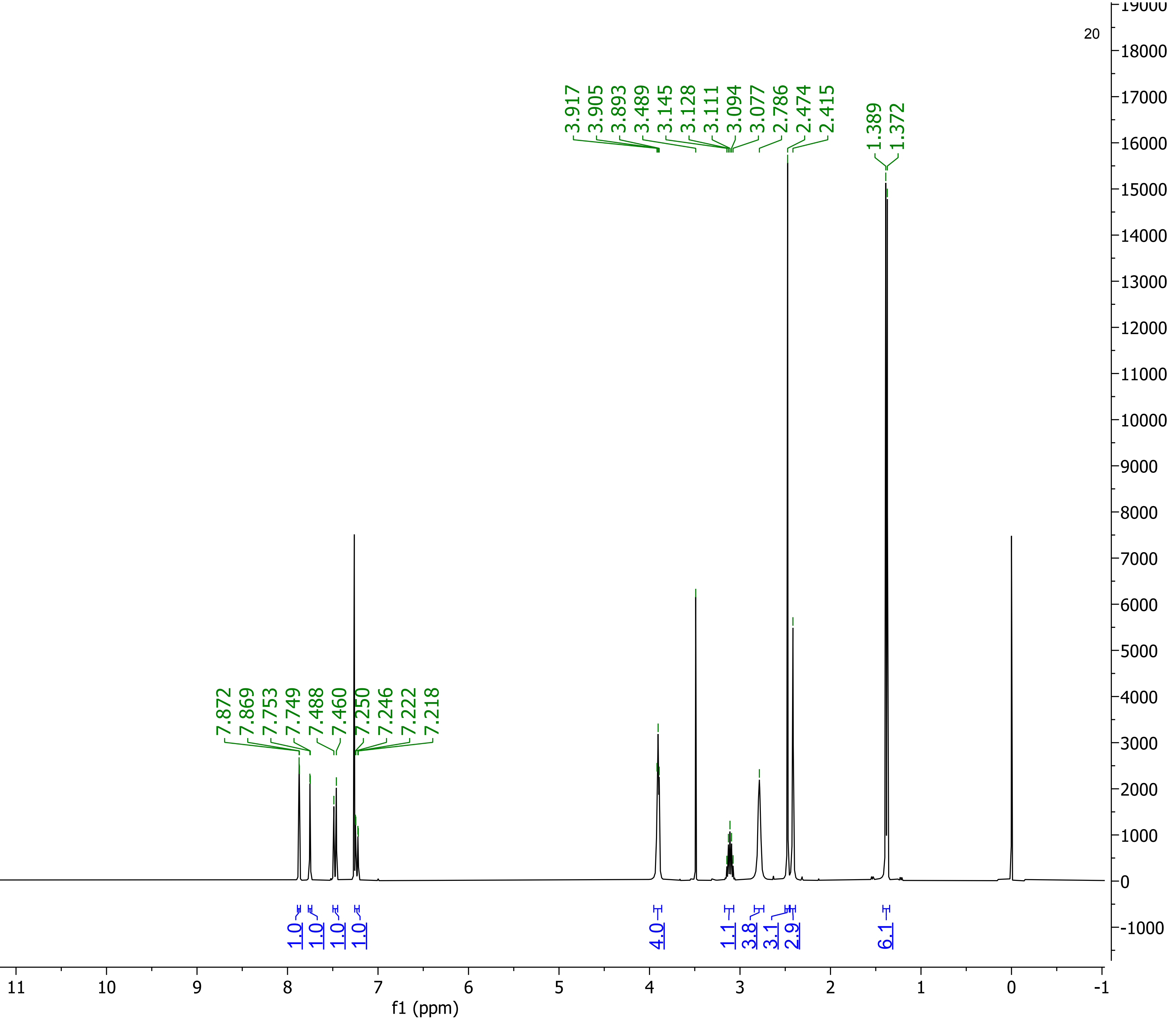
3.917
3.905
3.893
3.489
3.145
3.128
3.111
3.094
3.077
2.786
2.474
2.415
1.389
1.372
7.872
7.869
7.753
7.749
7.488
7.460
7.250
7.246
7.222
7.218
1.0
1.0
1.0
1.0
4.0
1.1
3.8
3.1
2.9
6.1
13
12
11
10
9
8
7
6
5
4
3
2
1
0
-1
f1 (ppm)
19000
18000
17000
16000
15000
14000
13000
12000
11000
10000
9000
8000
7000
6000
5000
4000
3000
2000
1000
0
-1000

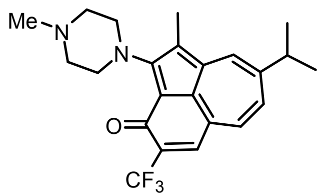

Me
N
N
O
CF3

10

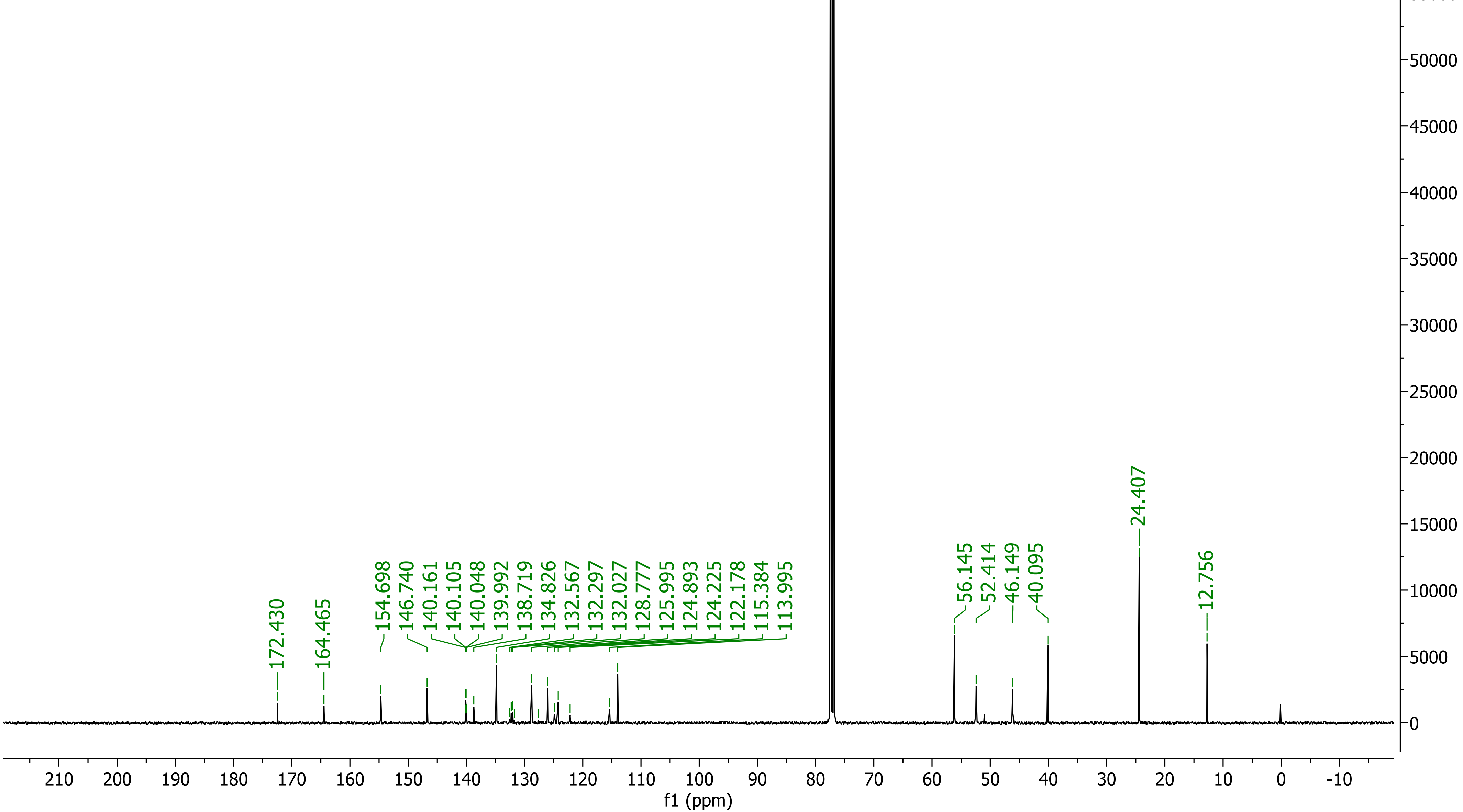

172.430
164.465
154.698
146.740
140.161
140.105
140.048
139.992
138.719
134.826
132.567
132.297
132.027
128.777
125.995
124.893
124.225
122.178
115.384
113.995
56.145
52.414
46.149
40.095
24.407
12.756
f1 (ppm)

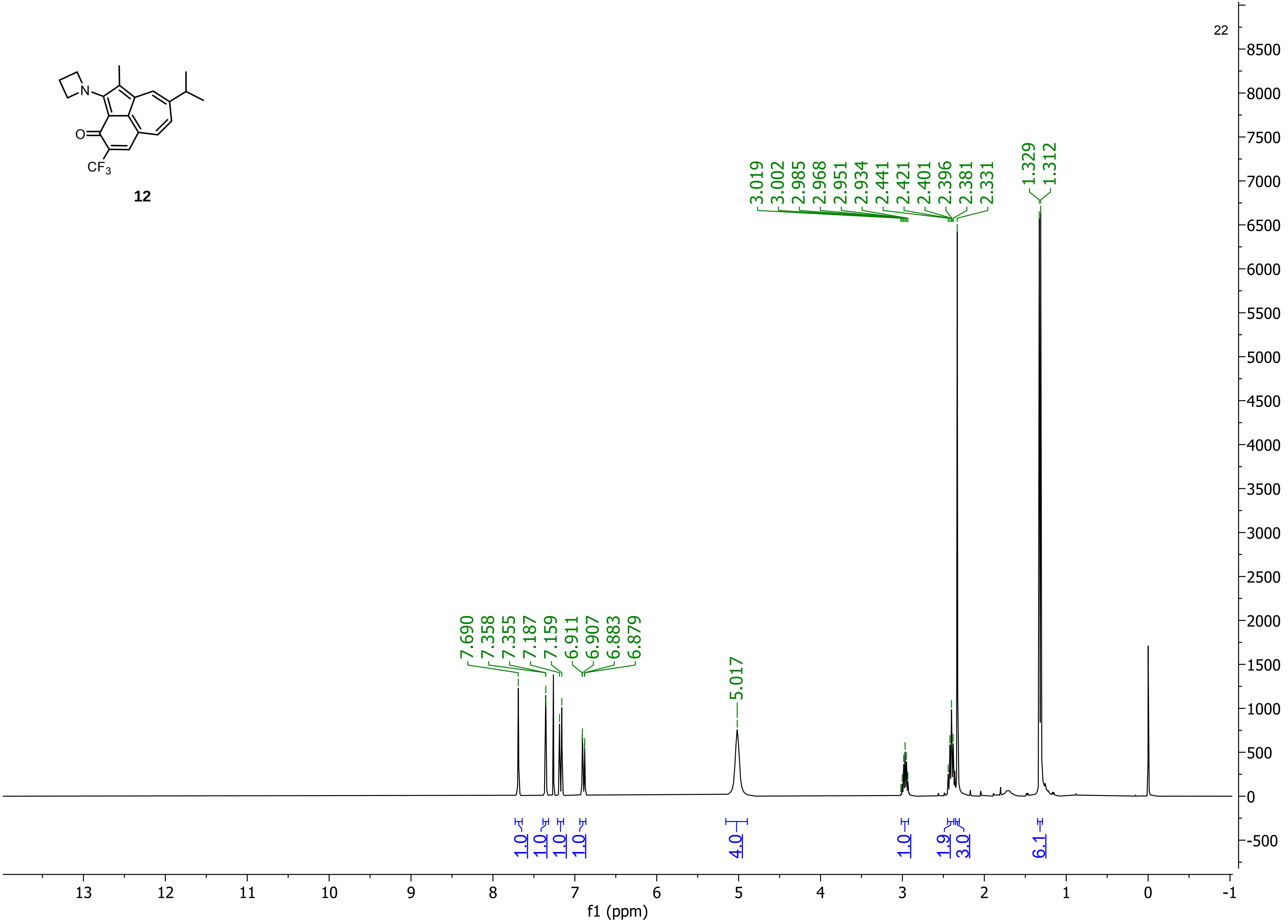
N
O
CF_3
12
7.690
7.358
7.355
7.187
7.159
6.911
6.907
6.883
6.879
5.017
3.019
3.002
2.985
2.968
2.951
2.934
2.441
2.421
2.401
2.396
2.381
2.331
1.329
1.312
1.0
1.0
1.0
1.0
4.0
1.0
1.9
3.0
6.1
f1 (ppm)

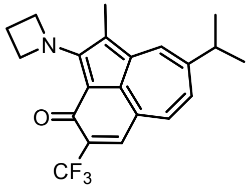
N
O
CF3

12

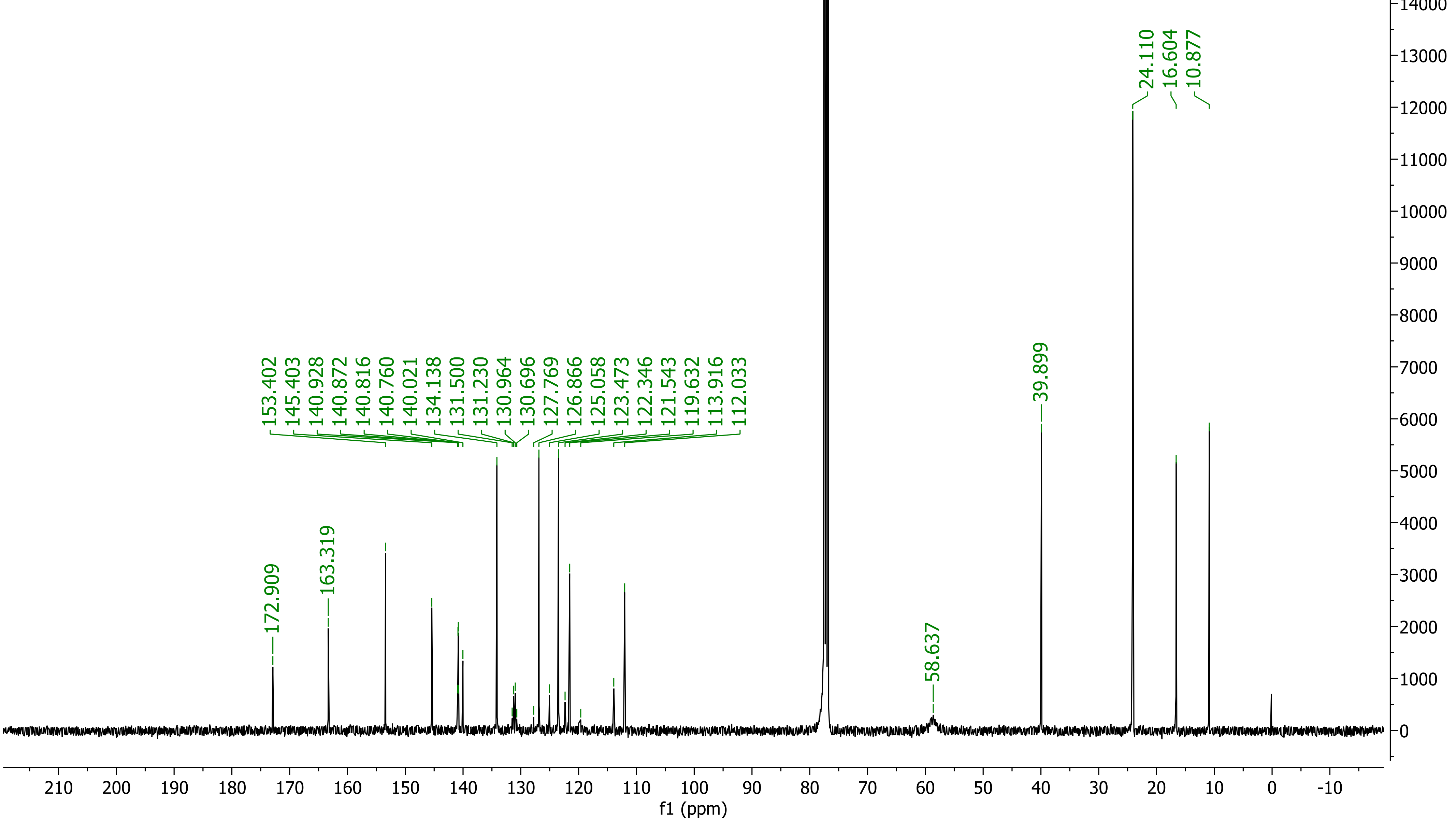
172.909
163.319
153.402
145.403
140.928
140.872
140.816
140.760
140.021
134.138
131.500
131.230
130.964
130.696
127.769
126.866
125.058
123.473
122.346
121.543
119.632
113.916
112.033
58.637
39.899
24.110
16.604
10.877
f1 (ppm)
