# Supplementary material for: A Covalent Calmodulin Inhibitor as a Tool to Study Cellular Mechanisms of K-Ras-Driven Stemness
Source: Front Cell Dev Biol. 2021 Jul 8;9:665673. doi: 10.3389/fcell.2021.665673 (PMC8296985; doi:10.3389/fcell.2021.665673)
Supplement: Supplementary file 2 [file Data_Sheet_2.PDF]

Supplementary Material

1 Supplementary Figures

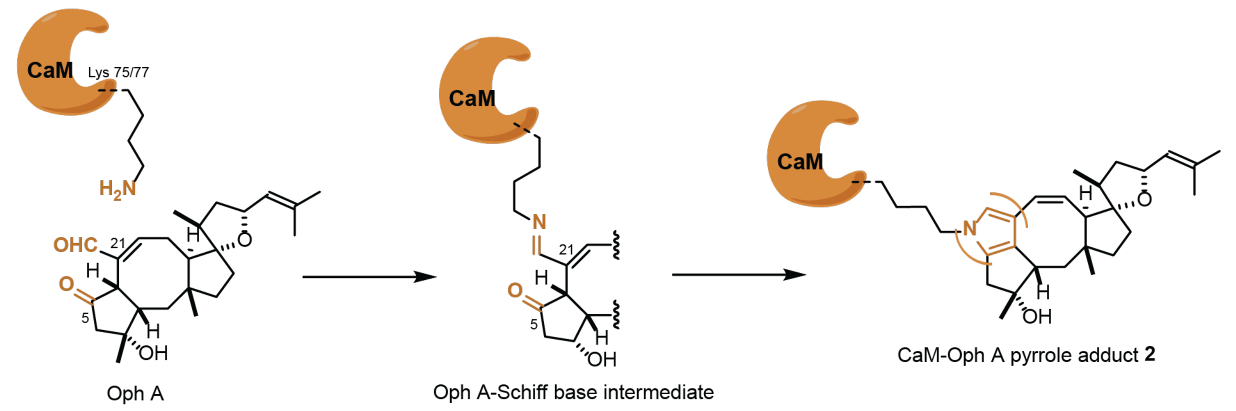

Scheme S1. Schematic drawing of covalent reaction of OphA with lysines of CaM. The irreversible pyrrole adduct **2** is formed from the Schiff base intermediate.

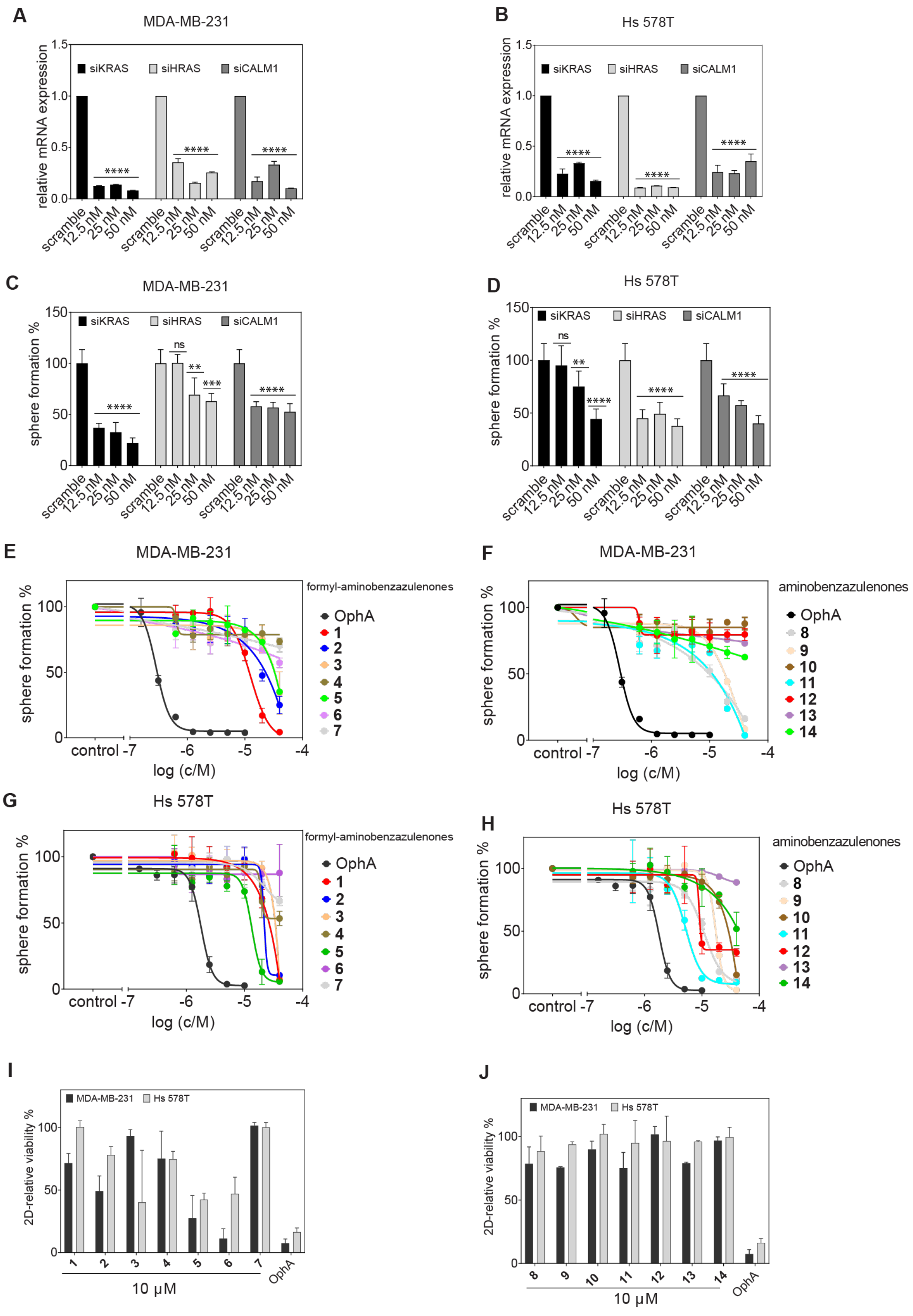

Figure S1. 3D spheroid formation data and knockdown controls. (A,B) RT-qPCR based evaluation of the relative mRNA expression of KRAS, HRAS or CALM1 upon siRNA mediated knockdown in MDA-MB-231 (A) and Hs 578T (B) cells. The siRNA against the KRAS gene (siKRAS), HRAS (siHRAS) and a mix of four siRNAs Targeting the CALM1 gene (siCALM1)

were used for this experiment. Data represent mean values ± SD, n = 2. **(C,D)** Effect of the knockdown of KRAS, HRAS or CALM1 genes on the 3D spheroid formation of MDA-MB-231 (C) and Hs 578T (D) cells in low attachment plates without serum. Data represent mean values ± SD, n = 2. **(E-H)** Dose response curves showing the effect of formyl aminobenzazulenones (0.6 – 40 µM), aminobenzazulenones (0.6 – 40 µM) and OphA (0.2 – 10 µM) on 3D spheroid formation of MDA-MB-231 (E, F) and Hs 578T (G, H) cells, as indicated. Cells were grown as 3D spheroids under low attachment and serum free conditions and then treated 3 days with compounds. The data were fit to log (inhibitor) vs response – variable slope (four parameters) equation using the Prism (GraphPad) software. Note that the actual curve fitting for DSS calculations was done on the breeze-site (https://breeze.fimm.fi/). Data represent mean values ± SD, n ≥ 3. **(I,J)** The relative viability of the compounds were assessed in alamarBlue assay. Cells were grown as 2D adherent monolayers overnight and then treated for 72 h with 1 µM OphA or 10 µM of the indicated benzazulenones. Data represent mean values ± SD, n ≥ 2.

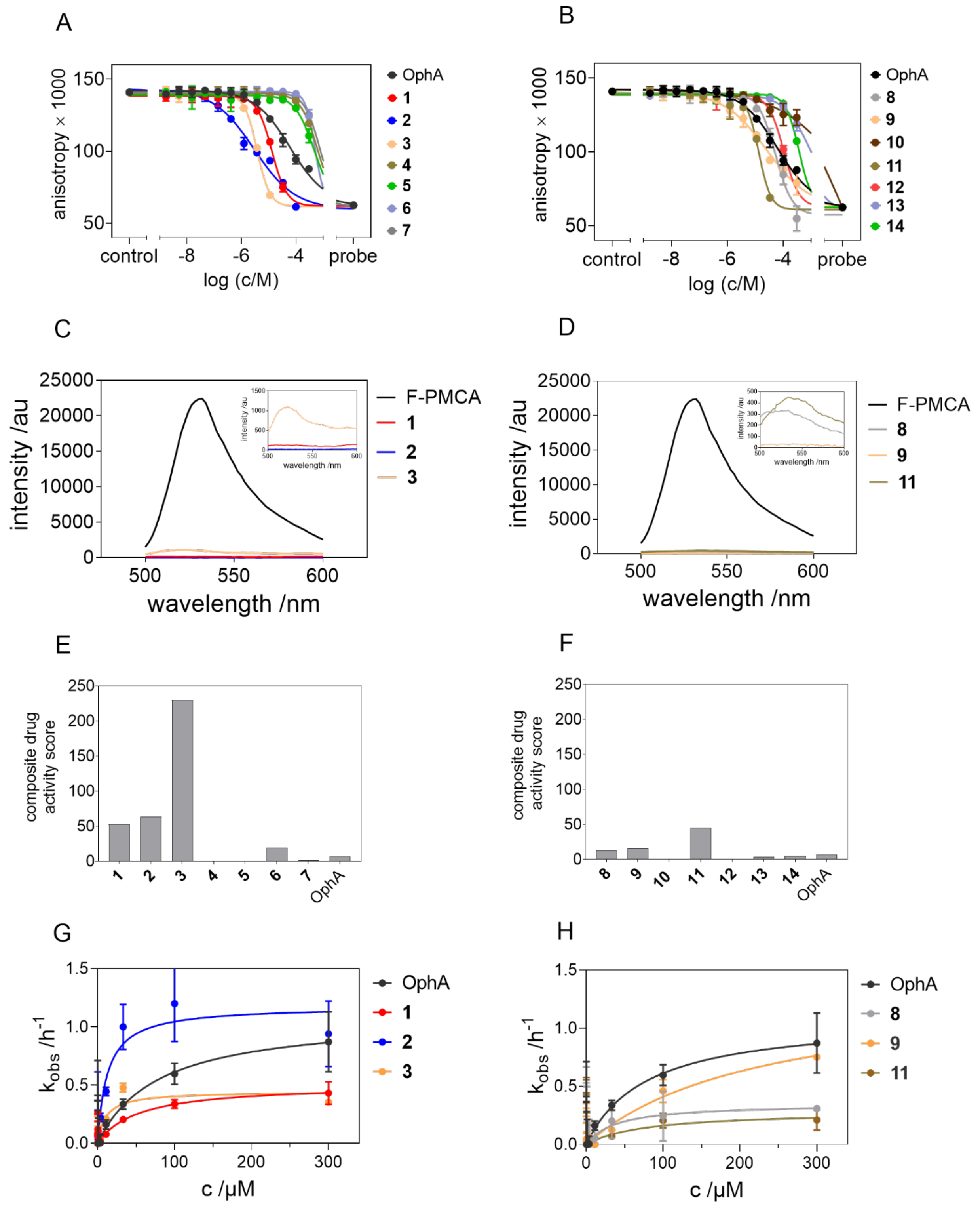

Figure S2. Assessment of binding affinity of benzazulenones to CaM by a fluorescence polarization assay and composite drug activity score. (**A,B**) Displacement of complex of 100 nM

CaM and 10 nM F-PMCA peptide by formyl aminobenzazulenones (A) and aminobenzazulenones (B) after 24 h incubation. Data represent mean values ± SD, n = 2. (**C,D**) Comparison of autofluorescence of compounds with that of F-PMCA. Fluorescence emission spectra were recorded with excitation at 475 ± 10 nm for benzazulenone compounds at 5 mM concentration and 10,000× lower concentration of 0.5 µM for F-PMCA peptide using a Clariostar plate reader. **(E,F)** Visualization of composite drug activity scores for formyl aminobenzazulenones (E) and aminobenzazulenones (F). Note that **3** was chemically not as stable and therefore deprioritized. **(G,H)** Hyperbolic fit to the observed rate constants in order to derive the covalent step rate constant k_2 and the affinity constant for the non-covalent intermediate K_i.

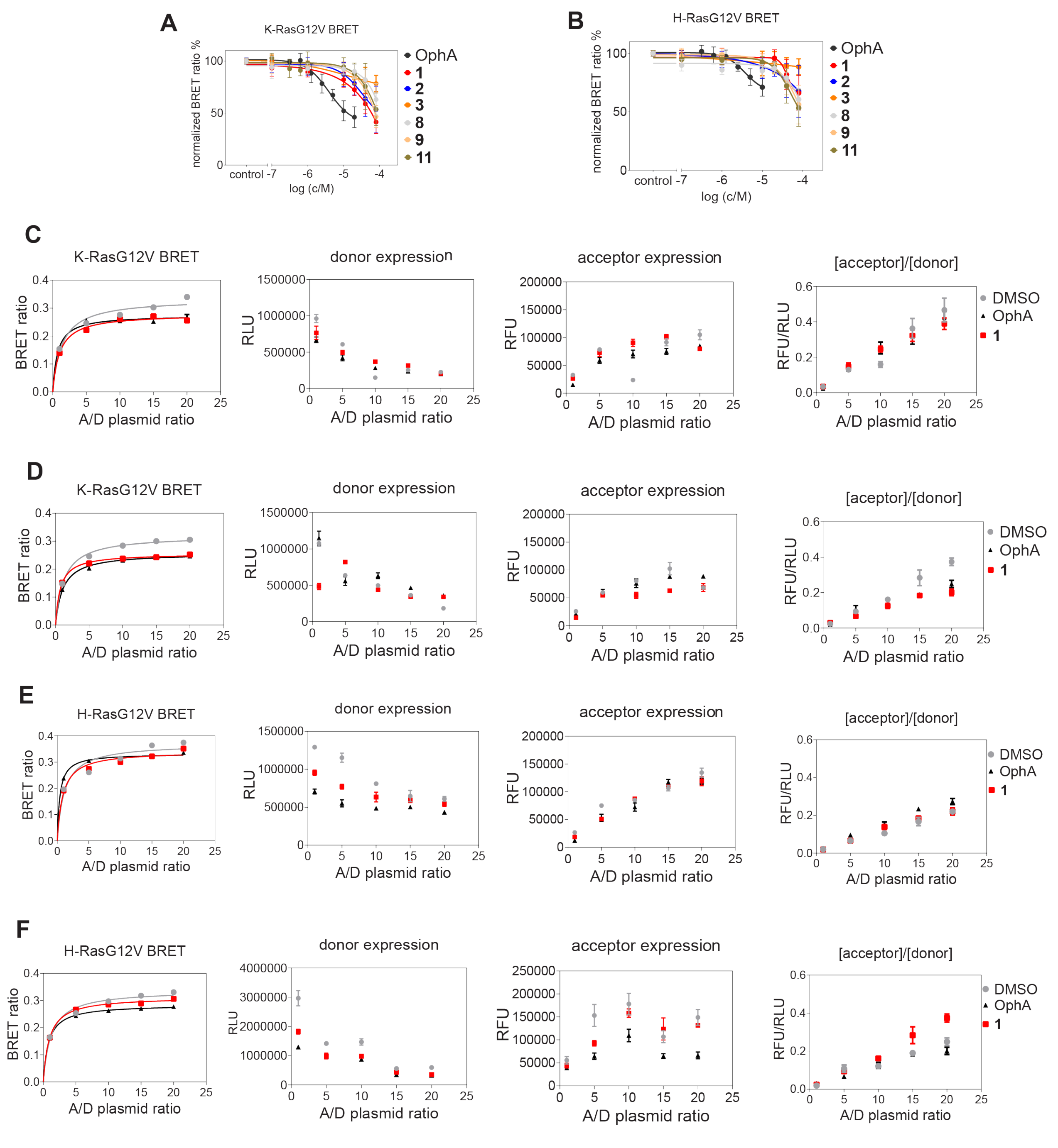

Figure S3. Nanoclustering-BRET assays confirm K-Ras selectivity and faster reactivity of 1 in cells. (**A,B**) Dose response curves of top six benzazulenones (0.1 – 80 µM) and OphA (0.3 – 20 µM) on K-RasG12V (A) and H-RasG12V (B) nanoclustering BRET. The A/D plasmid ratio was 4/1. Data represent mean values ± SD, n ≥ 3. The data were fit into log(inhibitor) vs. variable response (four parameters) function was used in the Prism (GraphPad) software to obtain the dose response curve. The actual curve fitting for DSS_3 calculation was done on the breeze-site (https://breeze.fimm.fi/). (**C-F**) BRET ratio vs. acceptor/donor plasmid ratio (A/D plasmid ratio), donor and acceptor expression data of BRET donor saturation titration experiments. Data from BRET pairs of Rluc8- and GFP2-tagged K-RasG12V (E,F) and corresponding H-RasG12V (G,H)

after 24h treatment with DMSO (0.2% v/v in growth medium), OphA (2.5 μM) or **1** (20 μM). Each figure group represents individual biological repeats with (left to right) BRET ratio plotted against acceptor/donor plasmid ratio (A/D plasmid ratio), and then donor expression (RLU), acceptor expression (RFU) and relative expression of the two (RFU/RLU) plotted against acceptor/donor plasmid ratio (A/D plasmid ratio). Each biological repeat is the mean of four technical replicates (± SD). The BRET ratio vs. relative expression data were fit with a hyperbolic function in Prism to obtain the $BRET_{max}$ and $BRET_{50}$ values.

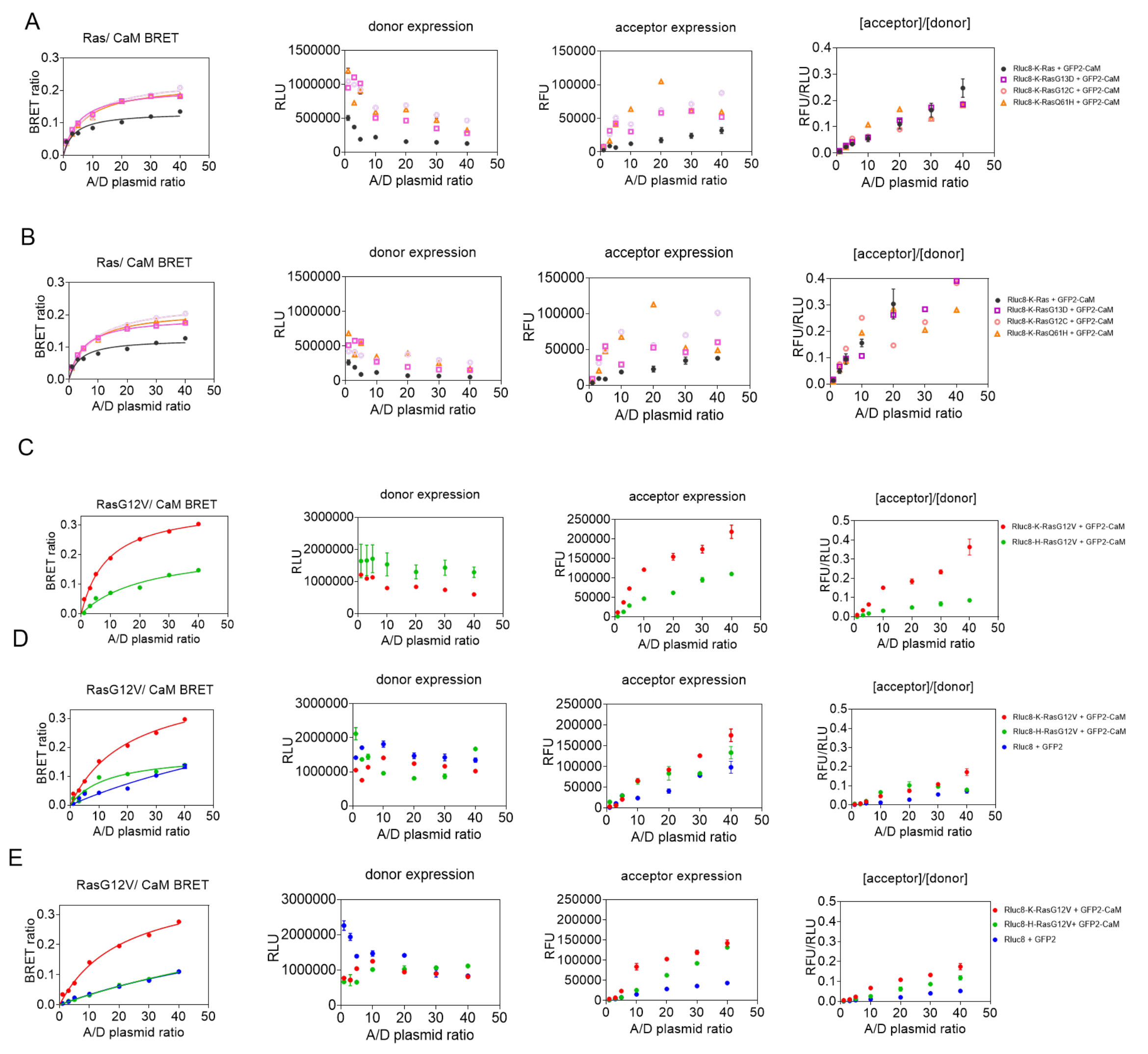

Figure S4. Repeat data of Ras/ CaM BRET experiments. The above figure represents BRET ratio vs. acceptor/donor plasmid ratio (A/D plasmid ratio), donor and acceptor expression data of BRET donor saturation titration experimental repeats for various Ras/ CaM titration curves. **(A,B)** Shows donor saturation titration curves for Rluc8-tagged wild type K-Ras and various G-domain mutants (G12C, G13D, and Q61H) with GFP2-CaM. **(C,D,E)** Shows donor saturation titration curves for Rluc8-K-RasG12V and Rluc8-H-RasG12V with GFP2-CaM. Rluc8 vs GFP2 alone were used to control for unspecific interactions. Each figure group represents individual biological repeats with (left to right) BRET ratio plotted against acceptor/donor plasmid ratio (A/D plasmid ratio), and then donor expression (RLU), acceptor expression (RFU) and relative expression of the two (RFU/RLU) plotted against acceptor/donor plasmid ratio (A/D plasmid ratio). Each biological repeat is the mean of four technical replicates (± SD). The BRET ratio vs. relative expression data were fit with a hyperbolic function in Prism to obtain the $BRET_{max}$ and $BRET_{50}$ values.

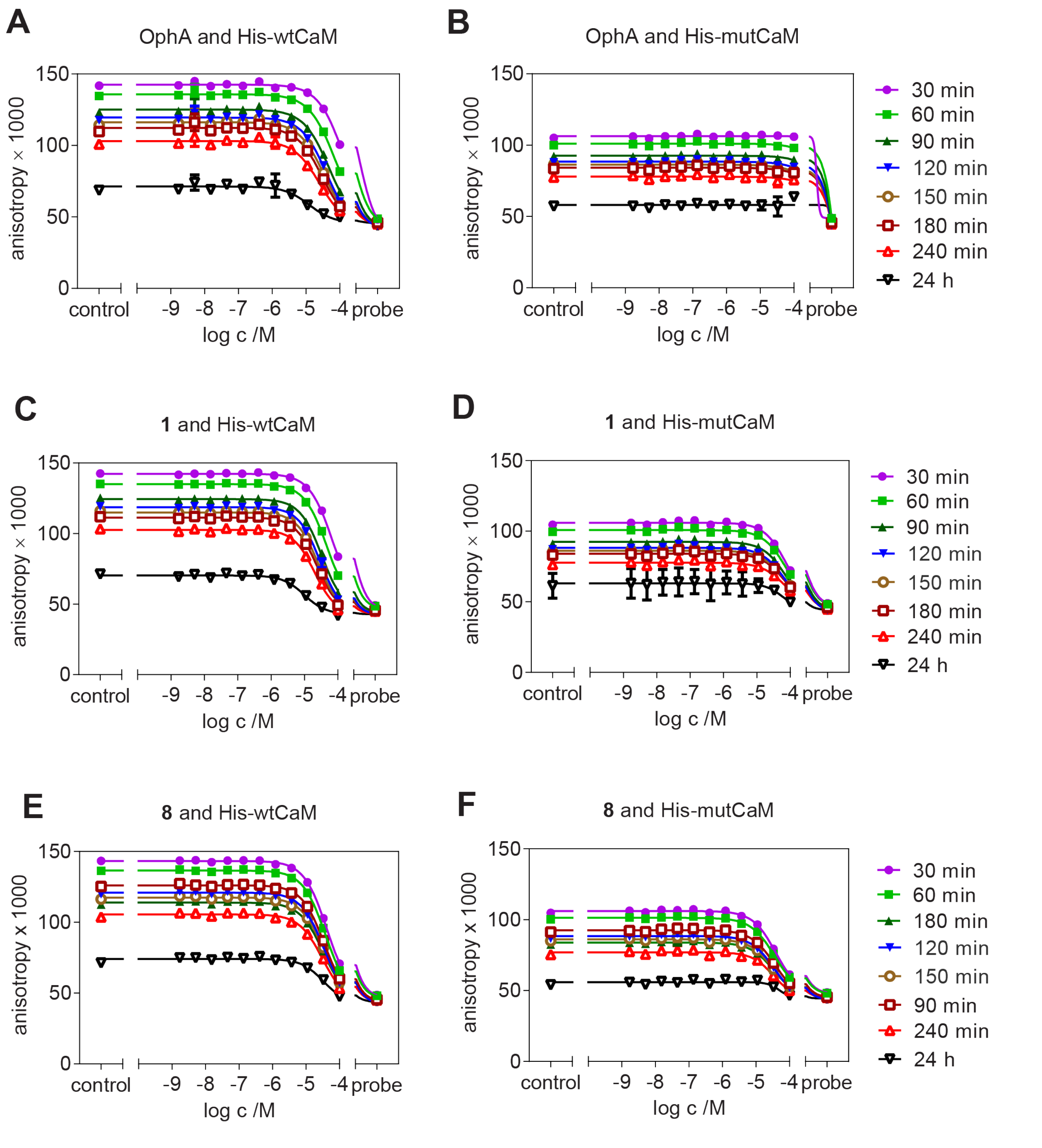

Figure S5. Lysine-dependent CaM-binding activity of OphA, 1 and 8. Displacement of complex of 50 nM His-wtCaM or His-mutCaM and 5 nM F-CaMKII peptide by OphA (**A,B**), **1** (**C,D**) and **8** (**E,F**) at various incubation times. Data represent mean values ± SD, n = 3. The IC_{50} value calculated at each time point was plotted against the time of incubation on **Figure 5**.

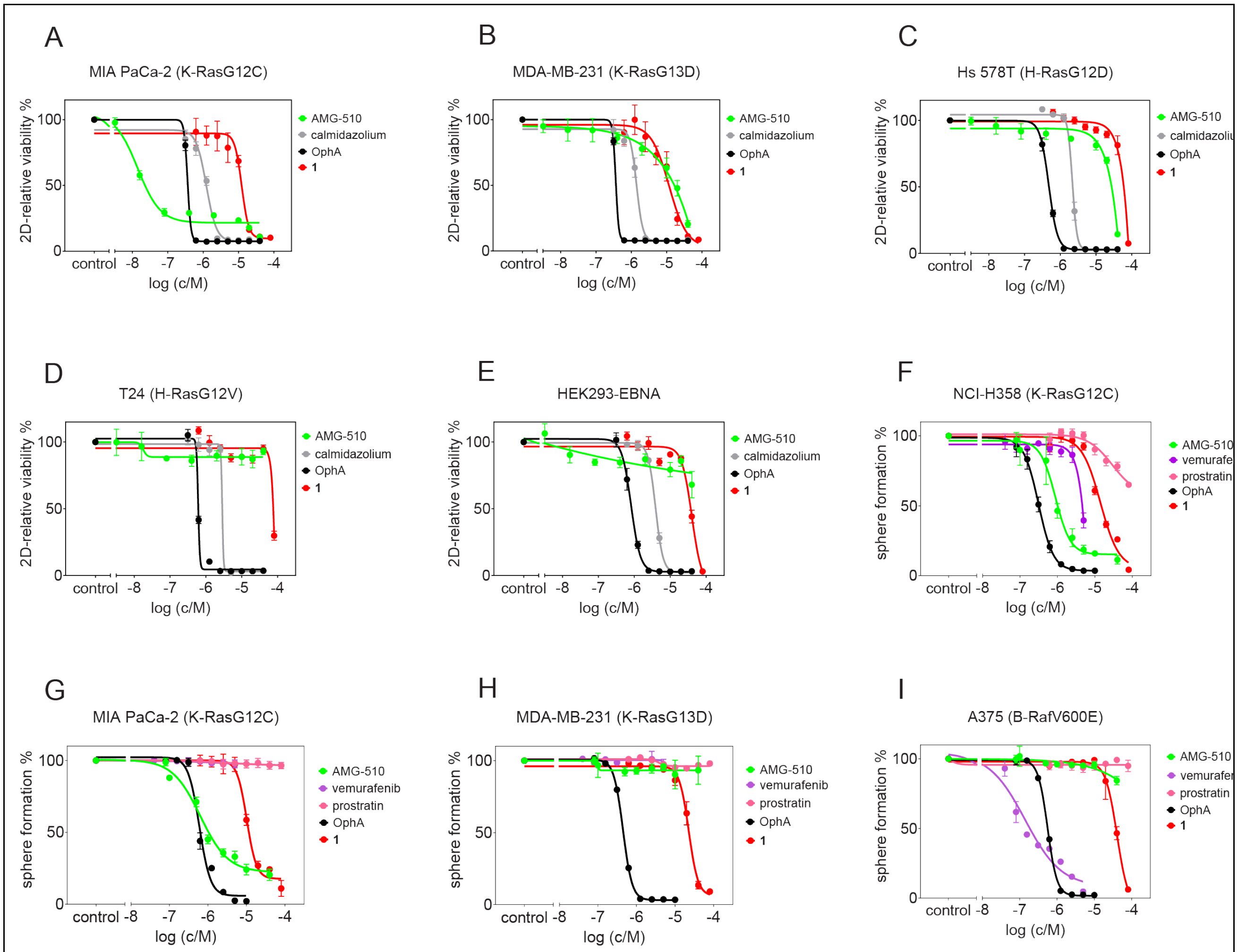

Figure S6. Dose-response curves of benchmarking of 1. (**A-E**) Comparison of effects of compounds on 2D monolayers derived from MDA-MB-231 (A), MIA PaCa-2 (B), Hs 578T (C), T24 (D) and HEK293-EBNA (E). Cells were treated with concentration range of 0.6 µM – 80 µM (**1**), 0.3 µM – 40 µM (calmidazolium and OphA) and 0.003 µM – 40 µM (AMG-510). Data represent mean values ± SD, n = 3. **(F-I)** Assessment of the effects of compound on 3D spheroids derived from NCI-H358 (F), MIA PaCa-2 (G), MDA-MB-231 (H) and A375 (I). Cells were treated with indicated compounds. **1** was tested at 1.3 – 80 µM. K-RasG12C inhibitor AMG-510 was tested at 0.6 – 40 µM. Additional compounds tested were vemurafenib (0.3 – 20 µM), OphA (0.3 – 20 µM) and prostratin (0.6 – 80 µM). The data were fit to log (inhibitor) vs response – variable slope (four parameters) equation using the Prism (GraphPad) software. Note that the actual curve fitting for DSS calculations was done on the breeze-site (https://breeze.fimm.fi/). Data represent mean values ± SD, n ≥ 2.

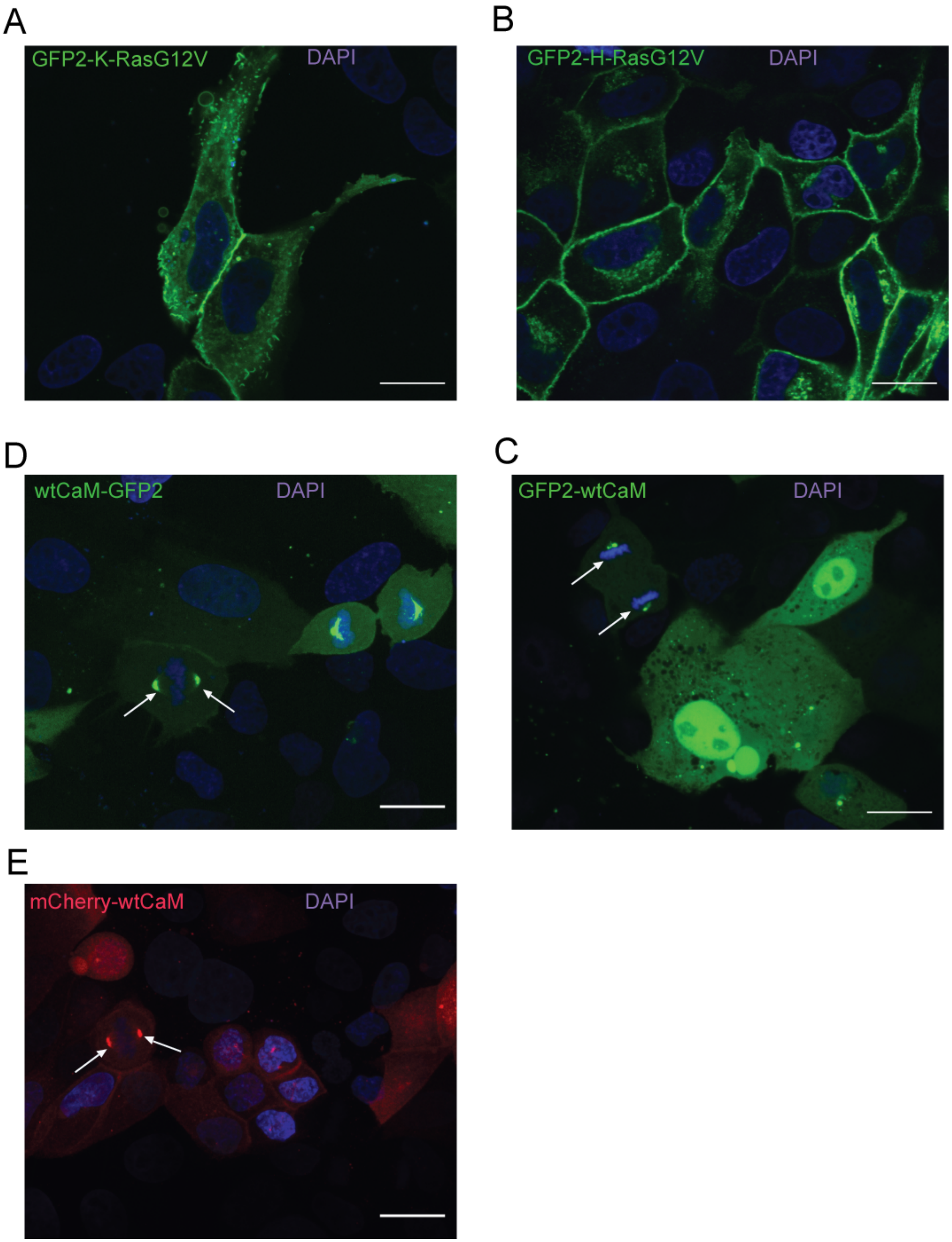

Figure S7. **Validation of cellular localization of tagged Ras- and CaM constructs**. (**A,B**) Predominant plasma membrane localization of GFP2-K-RasG12V (A), GFP2-H-RasG12V (B). (**D-E**) All CaM constructs, GFP2-wtCaM (C), wtCaM-GFP2 (D) and mCherry-wtCaM (E),

showed characteristic localization to the centrosomes (pointed arrows) and spindles in mitotic cells. Cell nuclei were stained with DAPI (blue). Scale bars, 20 µm.

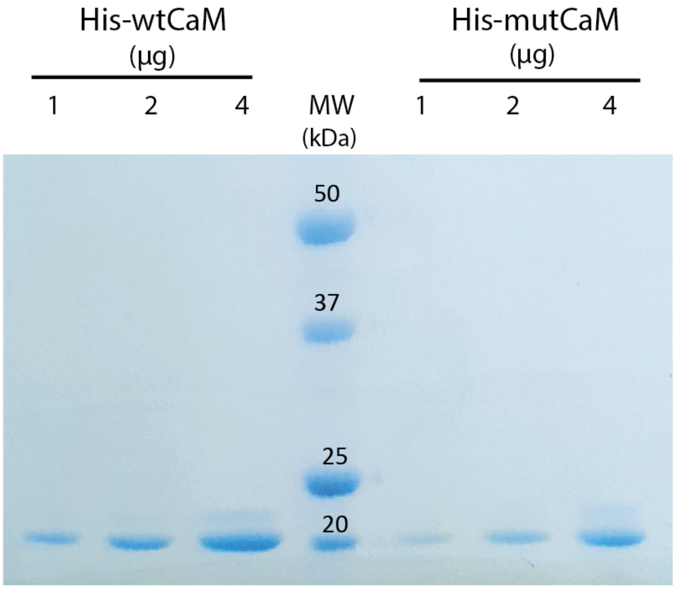

Figure S8. Analysis of purified proteins His-wtCaM and His-mutCaM by SDS PAGE. The gel was stained with ROTI Blue quick. Different amounts (1, 2 and 4 µg) of recombinant proteins were loaded on the gel.

2 Supplementary Tables

Table S1: Expression constructs generated by multi-site Gateway cloning

#	Recombinant plasmid	Entry clone 1	Entry clone 2	Entry clone 3	Destination vector
1	pDest305-CMV-Rluc8-K-RasG12V	CMV51p (C453-E04)	Rluc8 (C511-E03)	Hs. K-RasG12V	pDest-305
2	pDest305-CMV-Rluc8-H-RasG12V	CMV51p (C453-E04)	Rluc8 (C511-E03)	Hs. H-RasG12V	pDest-305
3	pDest305-CMV-GFP2-K-RasG12V	CMV51p (C453-E04)	pDONR235-GFP2-no stop	Hs. K-RasG12V	pDest-305
4	pDest305-CMV-GFP2-H-RasG12V	CMV51p (C453-E04)	pDONR235-GFP2-no stop	Hs. H-RasG12V	pDest-305
5	pDest305-CMV-Rluc8-K-Ras	CMV51p (C453-E04)	Rluc8 (C511-E03)	Hs. K-Ras	pDest-305
6	pDest305-CMV-Rluc8-H-Ras	CMV51p (C453-E04)	Rluc8 (C511-E03)	Hs. H-Ras	pDest-305
7	pDest305-CMV-Rluc8-K-RasG12C	CMV51p (C453-E04)	Rluc8 (C511-E03)	Hs. K-RasG12C	pDest-305
8	pDest305-CMV-Rluc8-K-RasQ61H	CMV51p (C453-E04)	Rluc8 (C511-E03)	Hs. K-RasQ61H	pDest-305
9	pDest305-CMV-Rluc8-K-RasG13D	CMV51p (C453-E04)	Rluc8 (C511-E03)	Hs. K-RasG13D	pDest-305
10	pDest305-CMV-Rluc8	CMV51p (C453-E04)	Rluc8 (C511-E03)	Stuffer (C125-E01)	pDest-305
11	pDest312-CMV-GFP2	CMV51p (C413-E36)	Stuffer (C125-E01)	pDONR257-GFP2-stop	pDest-307
12	pDest527-His-wtCaM	pDONR221-wtCaM	-	-	pDest-527
13	pDest527-His-mtCaM	pDONR221-mutCaM	-	-	pDest-527

Sources:

- All entry clones listed in the table with a code number in parenthesis and destination vectors were obtained from the RAS initiative, FNLCR-NCI, USA
- pDONR257-GFP2-stop (R2-L3) and pDONR235-GFP2-no stop (R5-R1) were commercially cloned at Genewiz Inc., USA. The GFP2 gene was synthesized and cloned into pDONR-235 and pDONR-257 vectors
- The wild type K-Ras4B and H-Ras and the mutant genes were from the RAS mutant collection V2.0 library (kit #1000000089), obtained from Addgene
- pDONR221-wtCaM and pDONR221-mutCaM were commercially obtained from Genecust, France. The wtCaM and mutCaM (containing K75Q, K77Q, and 148Q mutations) were gene synthesized and cloned into pDONR-221 vector using 5’ and 3’ BsrGI sites

Sequences of proteins encoded by plasmids used in the BRET experiments

Legend: Rluc8, GFP2, RAS, CaM

1. pDest305-CMV-Rluc8-K-RasG12V

MASKVYDPEQRKRMITGPQWWARCKQMNVLDSFINYYDSEKHAENAVIFLHGNATSSYLW
RHVVPHIEPVARCIIPDLIGMGKSGKSGNGSYRLLDHYKYLTAWFELLNLPKKIIFVGHDWG
AALAFHYAYEHQDRIKAIVHMESVVDVIESWDEWPDIEEDIALIKSEEGEKMVLENNFFVET
VLPSKIMRKLEPEEFAAYLEPFKEKGEVRRPTLSWPREIPLVKGGKPDVVQIVRNYNAYLRA
SDDLPKLFIESDPGFFSNAIVEGAKKFPNTEFVKVKGLHFLQEDAPDEMGKYIKSFVERVLKN
EQTTLYKKVGTMTEYKLVVVGAVGVGKSALTIQLIQNHFVDEYDPTIEDSYRKQVVIDGET
CLLDILDTAGQEEYSAMRDQYMRTGEGFLCVFAINNTKSFEDIHHYREQIKRVKDSEDVPM
VLVGNKCDLPSRTVDTKQAQDLARSYGIPFIETSAKTRQGVDDAFYTLVREIRKHKEKMSK
DGKKKKKKSKTKCVIM

2. pDest305-CMV-Rluc8-H-RasG12V

MASKVYDPEQRKRMITGPQWWARCKQMNVLDSFINYYDSEKHAENAVIFLHGNATSSYLW
RHVVPHIEPVARCIIPDLIGMGKSGKSGNGSYRLLDHYKYLTAWFELLNLPKKIIFVGHDWG
AALAFHYAYEHQDRIKAIVHMESVVDVIESWDEWPDIEEDIALIKSEEGEKMVLENNFFVET
VLPSKIMRKLEPEEFAAYLEPFKEKGEVRRPTLSWPREIPLVKGGKPDVVQIVRNYNAYLRA
SDDLPKLFIESDPGFFSNAIVEGAKKFPNTEFVKVKGLHFLQEDAPDEMGKYIKSFVERVLKN
EQTTLYKKVGMTEYKLVVVGAVGVGKSALTIQLIQNHFVDEYDPTIEDSYRKQVVIDGETC
LLDILDTAGQEEYSAMRDQYMRTGEGFLCVFAINNTKSFEDIHQYREQIKRVKDSDDVPMV
LVGNKCDLAARTVESRQAQDLARSYGIPYIETSAKTRQGVEDAFYTLVREIRQHKLRKLNPP
DESGPGCMSCKCVLS

3. pDest305-GFP2-K-RasG12V

MVSKGEELFTGVVPILVELDGDVNGHKFSVSGEGEGDATYGKLTLKFICTTGKLPVPWPTLV
TTLSYGVQCFSRYPDHMKQHDFFKSAMPEGYVQERTIFFKDDGNYKTRAEVKFEGDTLVNR
IELKGIDFKEDGNILGHKLEYNYNSHNVYIMADKQKNGIKVNFKIRHNIEDGSVQLADHYQQ
NTPIGDGPVLLPDNHYLSTQSALSKDPNEKRDHMVLLEFVTAAGITLGMDELYKTSLYKKV
GTMTEYKLVVVGAVGVGKSALTIQLIQNHFVDEYDPTIEDSYRKQVVIDGETCLLDILDTAG
QEEYSAMRDQYMRTGEGFLCVFAINNTKSFEDIHHYREQIKRVKDSEDVPMVLVGNKCDLP
SRTVDTKQAQDLARSYGIPFIETSAKTRQGVDDAFYTLVREIRKHKEKMSKDGKKKKKKSK
TKCVIM

4. pDest305-GFP2-H-RasG12V

MVSKGEELFTGVVPILVELDGDVNGHKFSVSGEGEGDATYGKLTLKFICTTGKLPVPWPTLV
TTLSYGVQCFSRYPDHMKQHDFFKSAMPEGYVQERTIFFKDDGNYKTRAEVKFEGDTLVNR
IELKGIDFKEDGNILGHKLEYNYNSHNVYIMADKQKNGIKVNFKIRHNIEDGSVQLADHYQQ
NTPIGDGPVLLPDNHYLSTQSALSKDPNEKRDHMVLLEFVTAAGITLGMDELYKTSLYKKV
GMTEYKLVVVGAVGVGKSALTIQLIQNHFVDEYDPTIEDSYRKQVVIDGETCLLDILDTAGQ
EEYSAMRDQYMRTGEGFLCVFAINNTKSFEDIHQYREQIKRVKDSDDVPMVLVGNKCDLA
ARTVESRQAQDLARSYGIPYIETSAKTRQGVEDAFYTLVREIRQHKLRKLNPPDESGPGCMS
CKCVLS

5. pDest305-CMV-Rluc8-K-Ras

MASKVYDPEQRKRMITGPQWWARCKQMNVLDSFINYYDSEKHAENAVIFLHGNATSSYLW
RHVVPHIEPVARCIIPDLIGMGKSGKSGNGSYRLLDHYKYLTAWFELLNLPKKIIFVGHDWG
AALAFHYAYEHQDRIKAIVHMESVVDVIESWDEWPDIEEDIALIKSEEGEKMVLENNFFVET
VLPSKIMRKLEPEEFAAYLEPFKEKGEVRRPTLSWPREIPLVKGGKPDVVQIVRNYNAYLRA
SDDLPKLFIESDPGFFSNAIVEGAKKFPNTEFVKVKGLHFLQEDAPDEMGKYIKSFVERVLKN
EQTTLYKKVGTMTEYKLVVVGAGGVGKSALTIQLIQNHFVDEYDPTIEDSYRKQVVIDGET
CLLDILDTAGQEEYSAMRDQYMRTGEGFLCVFAINNTKSFEDIHHYREQIKRVKDSEDVPM
VLVGNKCDLPSRTVDTKQAQDLARSYGIPFIETSAKTRQGVDDAFYTLVREIRKHKEKMSK
DGKKKKKKSKTKCVIM

6. pDest305-CMV-Rluc8-H-Ras

MASKVYDPEQRKRMITGPQWWARCKQMNVLDSFINYYDSEKHAENAVIFLHGNATSSYLW
RHVVPHIEPVARCIIPDLIGMGKSGKSGNGSYRLLDHYKYLTAWFELLNLPKKIIFVGHDWG
AALAFHYAYEHQDRIKAIVHMESVVDVIESWDEWPDIEEDIALIKSEEGEKMVLENNFFVET
VLPSKIMRKLEPEEFAAYLEPFKEKGEVRRPTLSWPREIPLVKGGKPDVVQIVRNYNAYLRA
SDDLPKLFIESDPGFFSNAIVEGAKKFPNTEFVKVKGLHFLQEDAPDEMGKYIKSFVERVLKN
EQTTLYKKVGMTEYKLVVVGAGGVGKSALTIQLIQNHFVDEYDPTIEDSYRKQVVIDGETC
LLDILDTAGQEEYSAMRDQYMRTGEGFLCVFAINNTKSFEDIHQYREQIKRVKDSDDVPMV
LVGNKCDLAARTVESRQAQDLARSYGIPYIETSAKTRQGVEDAFYTLVREIRQHKLRKLNPP
DESGPGCMSCKCVLS

7. pDest305-CMV-Rluc8-K-RasG12C

MASKVYDPEQRKRMITGPQWWARCKQMNVLDSFINYYDSEKHAENAVIFLHGNATSSYLW
RHVVPHIEPVARCIIPDLIGMGKSGKSGNGSYRLLDHYKYLTAWFELLNLPKKIIFVGHDWG
AALAFHYAYEHQDRIKAIVHMESVVDVIESWDEWPDIEEDIALIKSEEGEKMVLENNFFVET
VLPSKIMRKLEPEEFAAYLEPFKEKGEVRRPTLSWPREIPLVKGGKPDVVQIVRNYNAYLRA
SDDLPKLFIESDPGFFSNAIVEGAKKFPNTEFVKVKGLHFLQEDAPDEMGKYIKSFVERVLKN
EQTTLYKKVGTMTEYKLVVVGACGVGKSALTIQLIQNHFVDEYDPTIEDSYRKQVVIDGET
CLLDILDTAGQEEYSAMRDQYMRTGEGFLCVFAINNTKSFEDIHHYREQIKRVKDSEDVPM
VLVGNKCDLPSRTVDTKQAQDLARSYGIPFIETSAKTRQGVDDAFYTLVREIRKHKEKMSK
DGKKKKKKSKTKCVIM

8. pDest305-CMV-Rluc8-K-RasQ61H

MASKVYDPEQRKRMITGPQWWARCKQMNVLDSFINYYDSEKHAENAVIFLHGNATSSYLW
RHVVPHIEPVARCIIPDLIGMGKSGKSGNGSYRLLDHYKYLTAWFELLNLPKKIIFVGHDWG
AALAFHYAYEHQDRIKAIVHMESVVDVIESWDEWPDIEEDIALIKSEEGEKMVLENNFFVET
VLPSKIMRKLEPEEFAAYLEPFKEKGEVRRPTLSWPREIPLVKGGKPDVVQIVRNYNAYLRA
SDDLPKLFIESDPGFFSNAIVEGAKKFPNTEFVKVKGLHFLQEDAPDEMGKYIKSFVERVLKN
EQTTLYKKVGTMTEYKLVVVGAGGVGKSALTIQLIQNHFVDEYDPTIEDSYRKQVVIDGET
CLLDILDTAGHEEYSAMRDQYMRTGEGFLCVFAINNTKSFEDIHHYREQIKRVKDSEDVPM
VLVGNKCDLPSRTVDTKQAQDLARSYGIPFIETSAKTRQGVDDAFYTLVREIRKHKEKMSK
DGKKKKKKSKTKCVIM

9. pDest305-CMV-Rluc8-K-RasG13D

MASKVYDPEQRKRMITGPQWWARCKQMNVLDSFINYYDSEKHAENAVIFLHGNATSSYLW
RHVVPHIEPVARCIIPDLIGMGKSGKSGNGSYRLLDHYKYLTAWFELLNLPKKIIFVGHDWG
AALAFHYAYEHQDRIKAIVHMESVVDVIESWDEWPDIEEDIALIKSEEGEKMVLENNFFVET
VLPSKIMRKLEPEEFAAYLEPFKEKGEVRRPTLSWPREIPLVKGGKPDVVQIVRNYNAYLRA
SDDLPKLFIESDPGFFSNAIVEGAKKFPNTEFVKVKGLHFLQEDAPDEMGKYIKSFVERVLKN
EQTTLYKKVGTMTEYKLVVVGAGDVGKSALTIQLIQNHFVDEYDPTIEDSYRKQVVIDGET
CLLDILDTAGQEEYSAMRDQYMRTGEGFLCVFAINNTKSFEDIHHYREQIKRVKDSEDVPM
VLVGNKCDLPSRTVDTKQAQDLARSYGIPFIETSAKTRQGVDDAFYTLVREIRKHKEKMSK
DGKKKKKKSKTKCVIM

10. pDest305-CMV-Rluc8

MASKVYDPEQRKRMITGPQWWARCKQMNVLDSFINYYDSEKHAENAVIFLHGNATSSYLW
RHVVPHIEPVARCIIPDLIGMGKSGKSGNGSYRLLDHYKYLTAWFELLNLPKKIIFVGHDWG
AALAFHYAYEHQDRIKAIVHMESVVDVIESWDEWPDIEEDIALIKSEEGEKMVLENNFFVET
VLPSKIMRKLEPEEFAAYLEPFKEKGEVRRPTLSWPREIPLVKGGKPDVVQIVRNYNAYLRA
SDDLPKLFIESDPGFFSNAIVEGAKKFPNTEFVKVKGLHFLQEDAPDEMGKYIKSFVERVLKN
EQTTLYKKVG

11. pDest312-CMV-GFP2

MVSKGEELFTGVVPILVELDGDVNGHKFSVSGEGEGDATYGKLTLKFICTTGKLPVPWPTLV
TTLSYGVQCFSRYPDHMKQHDFFKSAMPEGYVQERTIFFKDDGNYKTRAEVKFEGDTLVNR
IELKGIDFKEDGNILGHKLEYNYNSHNVYIMADKQKNGIKVNFKIRHNIEDGSVQLADHYQQ
NTPIGDGPVLLPDNHYLSTQSALSKDPNEKRDHMVLLEFVTAAGITLGMDELYK

12. pDest305-CMV-GFP2-CaM

MVSKGEELFTGVVPILVELDGDVNGHKFSVSGEGEGDATYGKLTLKFICTTGKLPVPWPTLV
TTLSYGVQCFSRYPDHMKQHDFFKSAMPEGYVQERTIFFKDDGNYKTRAEVKFEGDTLVNR
IELKGIDFKEDGNILGHKLEYNYNSHNVYIMADKQKNGIKVNFKIRHNIEDGSVQLADHYQQ
NTPIGDGPVLLPDNHYLSTQSALSKDPNEKRDHMVLLEFVTAAGITLGMDELYKKVGMADQ
LTEEQIAEFKEAFSLFDKDGDGTITTKELGTVMRSLGQNPTEAELQDMINEVDADGNGTIDFP
EFLTMMARKMKDTDSEEEIREAFRVFDKDGNGYISAAELRHVMTNLGEKLTDEEVDEMIRE
ADIDGDGQVNYEEFVQMMTAK

Sequences of purified proteins

Legend: His6, CaM

1. pDest527-His-wtCaM

MRSGSHHHHHHRSDITSLYKKVGMADQLTEEQIAEFKEAFSLFDKDGDGTITTKELGTVMR
SLGQNPTEAELQDMINEVDADGNGTIDFPEFLTMMARKMKDTDSEEEIREAFRVFDKDGNG
YISAAELRHVMTNLGEKLTDEEVDEMIREADIDGDGQVNYEEFVQMMTAK

2. pDest527-His-mutCaM

MRSGSHHHHHHRSDITSLYKKVGMADQLTEEQIAEFKEAFSLFDKDGDGTITTKELGTVMR
SLGQNPTEAELQDMINEVDADGNGTIDFPEFLTMMARQMQDTDSEEEIREAFRVFDKDGNG
YISAAELRHVMTNLGEKLTDEEVDEMIREADIDGDGQVNYEEFVQMMTAQ
